# Structural determinants for FAT10 activation and transfer from UBA6 to E2 enzymes

**DOI:** 10.1038/s41467-026-76603-3

**Published:** 2026-08-13

**Authors:** Cara J. Ellison, Carlos Riechmann, Evmorfia V. Dalietou, Michael D. R. Simmons, Emma C. Dodd, Paul R. Elliott

**Affiliations:** Department of Biochemistry, University of Oxford, South Parks Road, Oxford, OX1 3QU UK

**Keywords:** Ubiquitins, Cryoelectron microscopy, Enzymes

## Abstract

Attachment of the ubiquitin-like protein (UBL) FAT10 onto substrates targets them for proteasomal degradation. Like ubiquitin, FAT10 is activated by the E1 enzyme UBA6 then transferred to E2 enzymes, but mechanisms controlling ubiquitin versus FAT10 activation by UBA6 and FAT10 transfer onto E2s remain unclear. Using cryo-EM, we visualise all stages of FAT10 E1-E2 handover: adenylation, thiolation and transthiolation. We find that FAT10 monopolises UBA6 by out-competing ubiquitin for thiolation and blocking the adenylation domain, preventing further UBL recruitment and promoting FAT10 signalling. We profiled UBA6-compatible E2 enzymes and found FAT10 transfer is restricted to a select subset associated with specific cellular pathways. UBE2Z (USE1) showed highest activity followed by UBE2D2, UBE2J2 and UBE2S. Capturing FAT10 or ubiquitin transfer from UBA6 to UBE2Z reveals UBE2Z is highly specialised for FAT10 transfer. It simultaneously engages both FAT10 domains (UBL1 and UBL2) and co-ordinates the metabolite inositol hexakisphosphate (InsP_6_) bound within the UBA6 catalytic domain. This InsP_6_ co-ordination extends to other FAT10 compatible E2s. Together, our structural and biochemical analyses reveal regulatory mechanisms underpinning FAT10 activation and transfer. We define principles governing selective FAT10 transfer, highlighting favourable interactions with FAT10 C-terminal domain (UBL2) and stable UBA6 binding, ensuring controlled conjugation onto substrates.

## Abstract (web summary)

Cryo-EM captures FAT10 activation and transfer from UBA6 to E2 UBE2Z, revealing FAT10 monopolises UBA6. Efficient transfer requires E2 engagement of both FAT10 UBL domains, particularly UBL2, and co-ordination of InsP₆ in the UBA6 catalytic domain.

## Introduction

Targeted protein degradation pathways are essential for the regulation of eukaryotic cell processes and the maintenance of a healthy cell environment. For example, timely degradation of proteins drives transitions between key stages of the cell cycle^1^ whilst inability to clear misfolded, aggregated or surplus proteins is causative of several diseases including cancers, thalassemias and neurodegeneration^2–4^. Eukaryotic cells heavily employ the ubiquitination system as one such strategy to target proteins for degradation through the proteasome or autophagosome. In mammals, however, an additional ubiquitin-like protein (UBL) targets substrates for proteasomal degradation – FAT10 (HLA-F-adjacent transcript 10)^5–7^ – with roles in cell cycle control^8–10^, autophagy^11^ and recently metabolism^12^.

FAT10 expression profiles vary across cell types. FAT10 is constitutively expressed in certain immune cells^13^, but expression can be driven in all cell types that respond to the pro-inflammatory cytokines TNFα and IFNγ^9,13,14^. Furthermore, multiple cancers display elevated levels of FAT10, correlating with worsened prognosis^15,16^. FAT10 comprises two UBL domains (the N-terminal UBL1 and C-terminal UBL2) joined by a conserved, six-residue flexible linker^17^ (Supplementary Fig. 1) akin to the second cytokine-regulated UBL ISG15. However, distinct from ISG15, FAT10 is one of the most recently evolved UBLs, being only found in placental mammals and specifically the Boreoeutheria clade (Supplementary Fig. 1).

UBLs differ markedly in sequence from ubiquitin but share the conserved ubiquitin β-grasp fold. Similarly to ubiquitin, UBL conjugation onto substrates is achieved through a cascade of three enzyme families required for activation (E1), conjugation (E2) and ligation (E3)^18,19^. Transformation of the UBL’s inert C-terminus into an active macromolecule is required to initiate all UBL cascades. This is achieved by UBL-specific E1-activating enzymes that first adenylate the C-terminal Gly using ATP, creating UBL-AMP, then transfer the UBL onto its catalytic Cys to produce a high-energy thioester bond in a thioesterification reaction, also known as thiolation. Subsequent recruitment of E2s to the thioester-loaded E1 facilitates transfer of the UBL from the E1 active site Cys to that of the E2 in a transthioesterification reaction, more commonly referred to as transthiolation.

**Fig. 1:**
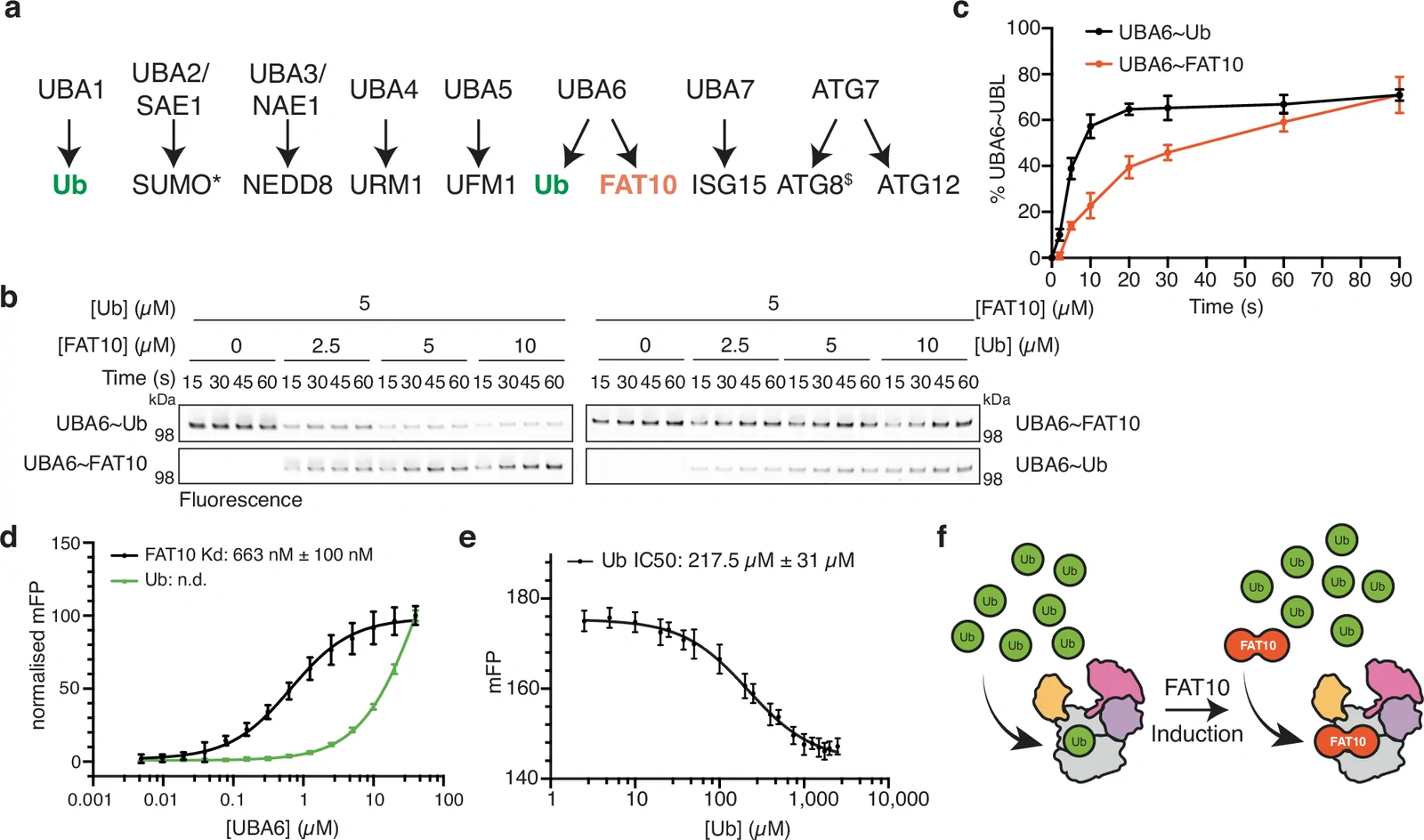
FAT10 out-competes ubiquitin for activation by UBA6 through high-affinity binding. **a** Most UBLs are activated by a dedicated E1 activating enzyme. UBA6 and ATG7 are the exceptions to this, each activating more than one distinct UBL. Mammalian E1 enzymes are shown. * denotes the four variants of SUMO. $ denotes the seven ATG8 family members (LC3A, LC3B, LC3B2, LC3C, GABARAP, GABARAP-L1, GATE-16). **b** FAT10 out-competes ubiquitin for loading onto UBA6 even when at sub-stoichiometric ratios in in vitro loading assays using Cy3-labelled FAT10 and Cy5-labelled ubiquitin. Gel is representative of three independent experiments. **c** FAT10 loads more slowly than ubiquitin onto UBA6 in time course in vitro loading assays. The graph represents the mean ± S.D. of three independent experiments. **d** FAT10 binds tighter to UBA6 than ubiquitin as measured by fluorescence polarisation (FP). n.d., non-determined. Graph represents mean ± S.D. of three independent experiments, each performed in technical triplicate. **e** Titrating ubiquitin to displace FAT10 bound to UBA6 (as measured by fluorescence polarisation) results in an IC50 of ~200 μM. Graph represents mean ± S.D. of three independent experiments, each performed in technical triplicate. **f** Despite lower cellular FAT10 levels compared to ubiquitin, FAT10 gains access to UBA6 through a high-affinity interaction.

Consequently, E1 enzymes are responsible for the specificity of UBL activation and E2 recruitment, thereby creating dedicated UBL cascades, ultimately bringing about UBL-distinct cellular events. Although some UBL-conjugating E2s display mild cross-reactivity with ubiquitin^20^, most UBLs are activated by dedicated E1s distinct from the E1s responsible for ubiquitin activation (UBA1 and UBA6) (Fig. 1a). FAT10, however, must compete with ubiquitin for activation by the bi-specific E1, UBA6. Furthermore, while UBA6 transfers ubiquitin to 15 E2s^21,22^, it is not known whether all E2s that partner with UBA6 can also accept FAT10, hindering identification and understanding of FAT10-controlled pathways.

Using cryo-EM visualisation across all stages of FAT10 activation and transfer, we reveal the mechanisms by which UBA6 balances the activation of both ubiquitin and FAT10, and we determine the principles governing optimal FAT10 transfer to E2 enzymes. Further to this, in vitro profiling of E2 enzymes identifies only a handful of UBA6 ubiquitin-active E2s that robustly receive FAT10, indicating FAT10 operates through specialised circuits governed by E2-UBL specificity.

## Results

### FAT10 out-competes ubiquitin for loading onto UBA6

UBA6 activates both ubiquitin and FAT10, but FAT10 levels are normally very low in most cell types^9,13,14^. However, upon IFNγ/TNFα stimulation, FAT10 levels increase^9,13,14^. We first investigated how FAT10 levels affect ubiquitin loading and sought to compare ubiquitin and FAT10 loading characteristics. When presented with a choice between ubiquitin and FAT10, UBA6 preferentially loaded FAT10 in in vitro loading assays (Fig. 1b, left panel). Even half the molar amount of FAT10 outcompeted ubiquitin loading, whereas titrating ubiquitin did not markedly decrease FAT10 loading (Fig. 1b, right panel). We observed FAT10 loads slower than ubiquitin, with maximum loading achieved by ~ 90 s compared to within 30 s for ubiquitin (Fig. 1c), and a previous study determined FAT10 adenylation occurs 1.6× slower than ubiquitin^23^. This suggests FAT10 does not use a kinetic advantage for preferential loading onto UBA6, raising the question of how FAT10 out-competition is achieved. We assessed whether this was due to a binding effect. Using fluorescence polarisation assays, we measured a high-affinity interaction between UBA6 and FAT10 (Kd 663 nM ± 100 nM) whilst weak binding to ubiquitin was observed with no determinable Kd within the concentration range of UBA6 tested (Fig. 1d), in line with earlier reports^23^.

We then determined the ubiquitin concentration required to overcome FAT10 binding (Fig. 1e) and recorded an IC50 of approximately 200 µM. In a cellular context, the ubiquitin concentration is ~85 µM^24^ and so although FAT10 is not expressed in most cell types, we speculate that upon FAT10 induction its higher affinity would allow FAT10 to gain access over ubiquitin (Fig. 1f). Together, our data shows FAT10 utilises a high affinity binding mode to out-compete ubiquitin, necessary to compensate for its lower expression levels and slower loading onto UBA6.

### FAT10 thiolation blocks activation of a second UBL

E1 enzymes perform complex choreography to bring the E1 catalytic Cys, found within the second catalytic cysteine half domain (SCCH), into proximity with the adenyl-UBL intermediate within the active adenylation domain (AAD) for covalent attachment (thiolation). This requires a dramatic rotation of the SCCH domain by ~130°, triggered by pyrophosphate release^25,26^. The mechanism of ubiquitin thiolation onto its two cognate E1 enzymes, UBA1 and UBA6, has been well characterised^21,26–29^, which has shown that a second ubiquitin is adenylated to form a ‘doubly loaded’ E1 complex prior to E1-E2 transthiolation^21,29,30^. While a crystal structure of a chimeric UBA6^UBA1SCCH^ non-covalently bound to FAT10 in the absence of ATP has been obtained^31^, it is unknown how FAT10 activation and loading onto UBA6 is achieved and how this compares, or even impacts, the activation and loading cycle of ubiquitin.

**Fig. 2:**
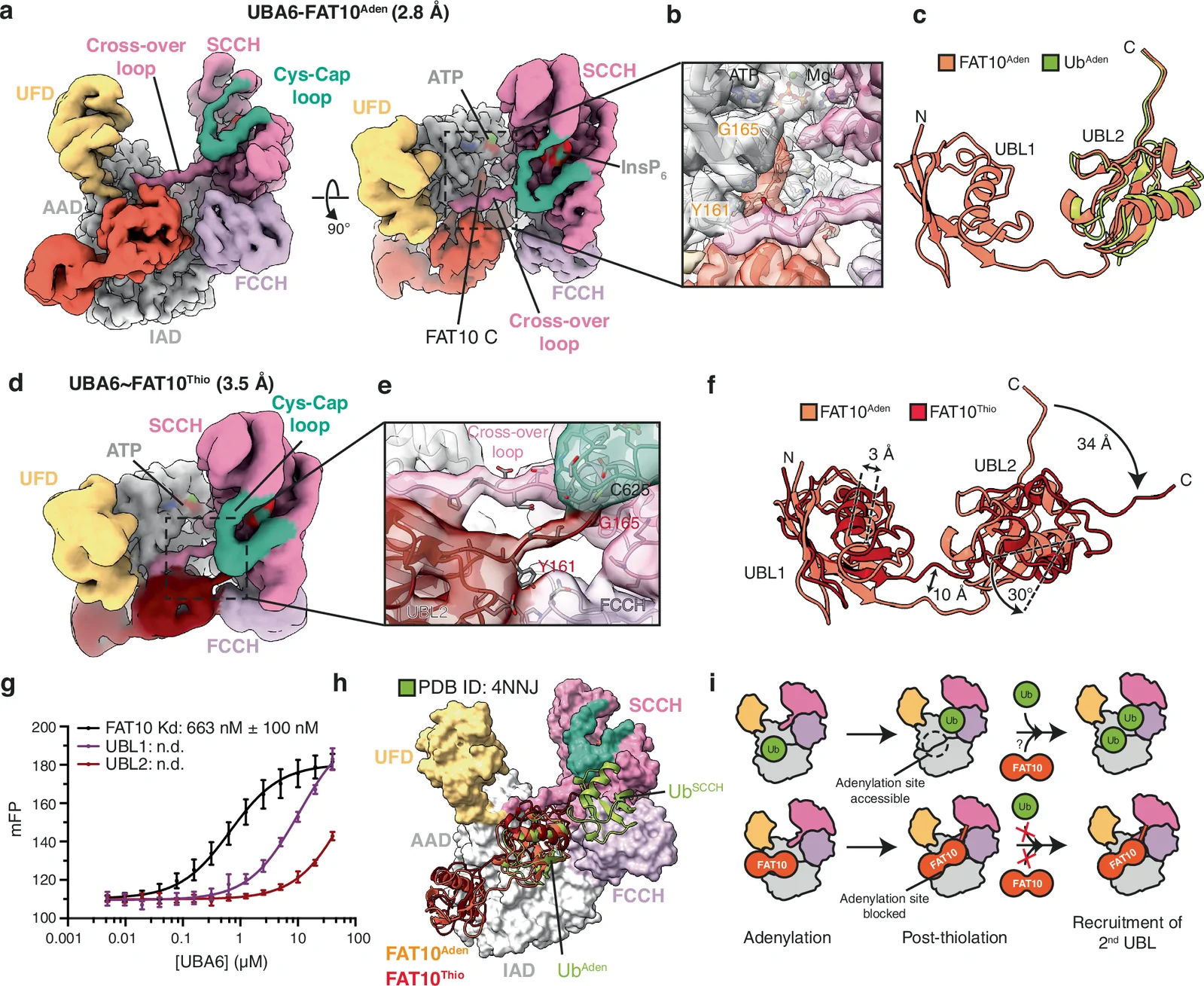
FAT10 thiolation is achieved through concomitant UBL2 rotation and anchoring by UBL1. **a** Cryo-EM density map of the adenylation-competent state (UBA6-FAT10^Aden^), visualising FAT10 bound across both the inactive adenylation domain (IAD) and active adenylation domain (AAD). **b** In this adenylation-competent state, the FAT10 C-terminal tail reaches under the cross-over loop into the ATP-binding site. **c** FAT10 UBL2 adopts the same position as ubiquitin (PDB ID 9QHI^21^) when in the adenylation state. **d** Cryo-EM density map of the post-thiolation state (UBA6~FAT10^Thio^) visualising FAT10 loaded onto UBA6 catalytic Cys625 in the SCCH domain with the FAT10 tail no longer occupying the ATP-binding site. **e** Electron density corresponding to the FAT10 C-terminal tail was observed reaching towards the UBA6 catalytic Cys625 in the SCCH domain. **f** The post-thiolation FAT10 state is achieved by a substantial rotation of UBL2, supported by UBL1 anchoring at the IAD. **g** UBL1 displays stronger binding to UBA6 than UBL2, but each individual UBL domain binds weaker than full-length FAT10 as determined by fluorescence polarisation (FP). n.d., non-determined. Graph represents mean ± S.D. of three independent experiments, each performed in technical triplicate. **h** FAT10 occupies the AAD in both adenylation and post-thiolation states in contrast to ubiquitin, which moves from SCCH to FCCH post-thiolation, allowing a second ubiquitin to be adenylated^28^. **i** FAT10 monopolises UBA6 through blockading the AAD after thiolation, preventing activation of a second FAT10 or ubiquitin until FAT10 is transferred onto an E2. This contrasts with the ubiquitin post-thiolation position, in which a second ubiquitin, or potentially a FAT10, can be activated in the AAD.

To address these questions, we captured FAT10 adenylation and thiolation by cryo-electron microscopy (cryo-EM) of UBA6 reacted with FAT10 and ATP (Table 1 and Supplementary Fig. 2). Particles could be classified into two states, corresponding to a pre-adenylation non-covalent complex comprising UBA6, FAT10 and ATP, and a UBA6 ~ FAT10 thioester-linked product. The adenylation state (UBA6–FAT10^Aden^), determined to an overall resolution of 2.8 Å (Fig. 2a and Supplementary Fig. 2a), shows FAT10 bound in the AAD with continuous density corresponding to the FAT10 tail protruding towards an ATP.Mg^II^ molecule in the ATP-binding site (Fig. 2b and Supplementary Fig. 3a). In line with other reported E1–UBL structures^29,32–35^, we do not observe FAT10-AMP owing to the reversibility of the adenylation reaction^36^. Superimposition of UBA6 from our UBA6–FAT10^Aden^ structure onto the crystal structure of UBA6–FAT10 in the absence of ATP^31^ revealed very small differences of the two FAT10 UBL moieties with an overall root mean square deviation (RMSD) of 1.65 Å (Supplementary Fig. 3b). In addition, UBL2 of FAT10 adopts a near-identical conformation to that of ubiquitin in our previously determined UBA6–Ub–BIRC6 structure^21^ (Fig. 2c). Three-dimensional variability analysis (3DVA) of the particle stacks revealed notable conformational heterogeneity, which could be attributed to distinct domain movements within the UFD and SCCH domains of UBA6 (Supplementary Fig. 2a–c) consistent with domain dynamics observed in previous UBA1 and UBA6 structures^21,29^.

**Table 1:** Summary of data collection and model refinement parameters for maps generated of UBA6-FAT10^Aden^, UBA6~FAT10^Thio^, UBA6–UbDha–UBE2Z (singly- or doubly-loaded) and UBA6–FAT10Dha–UBE2Z

	UBA6-FAT10^Aden^	UBA6~FAT10^Thio^	UBA6-FAT10Dha-UBE2Z	UBA6-UbDha-UBE2Z doubly loaded	UBA6-UbDha-UBE2Z singly loaded
	EMDB-56614	EMDB-56615	EMDB-56646	EMDB-56645	EMDB-56644
	PDB 28MJ	PDB 28MK	PDB 28MQ	PDB 28MP	PDB 28MO
**Data collection and processing**					
Microscope	Krios (COSMIC)	Krios (COSMIC)	Krios (COSMIC)	Krios (COSMIC)	Krios (COSMIC)
Magnification	105,000x	105,000x	105,000x	105,000x	105,000x
Voltage (kV)	300	300	300	300	300
Detector	Gatan K3 BioQuantum	Gatan K3 BioQuantum	Gatan K3 BioQuantum	Gatan K3 BioQuantum	Gatan K3 BioQuantum
Slid width (eV)	20	20	20	20	20
Electron exposure (e-/Å^2^)	42.2	42.2	42.7	40.1	40.1
Defocus range (μm)	−0.8, −2.4	−0.8, −2.4	−1, −2.5	−0.5, −2.5	−0.5, −2.5
Pixel size (Å)	0.85	0.85	0.85	0.85	0.85
Symmetry imposed	C1	C1	C1	C1	C1
Micrographs (no.)	47,574	47,574	14,526	6,392	6,392
Picking software	crYOLO, cryoSPARC	crYOLO, cryoSPARC	cryoSPARC	SIMPLE	SIMPLE
Initial particle images (no.)	17,888,095	17,888,095	7,743,765	4,791,444	4,791,444
Final particle images (no.)	795,937	223,766	233,302	80,561	125,636
Map resolution (Å)	2.8	3.5	3.0	3.1	3.0
FSC threshold	0.143	0.143	0.143	0.143	0.143
Map resolution range: 25^th^, 75^th^ percentile (Å)	2.7, 5.7	3.8, 6.6	3.1, 7.0	3.4, 7.3	3.2, 7.0
**Refinement (PHENIX v1.21.2)**					
Initial model used (PDB code)	7PYV, 9QIC	7PYV, 9QIC	7PYV, 9QIC, 5A4P	9QHI, AlphaFold prediction	9QHI, AlphaFold prediction
Model resolution (Å)	3.0	4.0	3.2	3.3	3.2
FSC threshold	0.5	0.5	0.5	0.5	0.5
Map sharpening *B* factor (Å^2^)	−148.9	−197.0	−118.6	−98.0	−101.4
Model composition					
Non-hydrogen atoms	9334	9333	11,204	11,186	10,583
Protein residues	1170	1170	1404	1397	1321
Ligands	3	3	1	2	2
*B* factors (Å^2^)					
Protein	164.1	240.7	108.5	168.7	156.4
Ligand	160.6	233.7	158.8	174.7	159.4
R.m.s deviations					
Bond lengths (Å)	0.002	0.002	0.002	0.005	0.004
Bond angles (°)	0.551	0.630	0.511	0.984	0.976
Validation					
MolProbity score	1.20	1.62	1.02	1.21	1.10
Clashscore	2.14	7.55	2.37	4.26	3.13
Poor rotamers (%)	0.87	0.87	0.32	0.16	0.43
Ramachandran plot					
Favoured (%)	96.66	96.74	98.07	98.20	98.25
Allowed (%)	3.34	3.26	1.93	1.80	1.75
Disallowed (%)	0.00	0.00	0.00	0.00	0.00

Secondly, the structure of FAT10 loaded onto the SCCH catalytic Cys (UBA6~FAT10^Thio^) was determined to 3.5 Å (Fig. 2d and Supplementary Fig. 2). Surprisingly, FAT10 continued to occupy the adenylation domain, although offset by ~ 3 Å. However, crucially, no density for the FAT10 C-terminal tail was observed in the AAD; it was instead visible extending towards the UBA6 catalytic Cys (Fig. 2d, e and Supplementary Fig. 2) underneath the protective Cys-Cap loop that is now in a re-ordered state post-thiolation^37^. The UBA6~FAT10^Thio^ structure displayed greater structural heterogeneity than UBA6-FAT10^Aden^ (Supplementary Fig. 2a–c). Whilst ~50% of the original particle stack could be ascribed to FAT10 being loaded, consistent with the proportion of UBA6~FAT10 in the sample (Supplementary Fig. 2a), further rounds of 3DVA and heterogeneous refinement were required to improve the local resolution of FAT10^UBL2^ in the deposited consensus map (Fig. 2d, e and Supplementary Fig. 2a–c).

Comparison of the two states revealed UBA6 is broadly unchanged (RMSD 0.60 Å) (Supplementary Fig. 3c). Instead, FAT10 undergoes the largest movement (Fig. 2f). While UBL1 undergoes a modest 3 Å translation between the FAT10^Aden^ and FAT10^Thio^ positions, UBL2 rotates 30° outwards, breaking the interactions of the UBL2-adenylation interface (Supplementary Fig. 3d). This substantial rotation is accompanied by a 10 Å translation of the UBL1-UBL2 linker, ultimately moving the FAT10 C-terminal tail 34 Å from the adenylation position towards the catalytic Cys of the SCCH domain (Fig. 2f), thus explaining the requirement of the flexible linker for FAT10 activation, transfer to UBE2Z and substrate conjugation^17^.

The distinct requirements for UBL1 rigidity and UBL2 flexibility are reflected in their UBA6 binding characteristics. Analysing individual UBL domains highlighted that UBL1 binds tighter than UBL2 (Fig. 2g), indicating UBL1 drives FAT10 recruitment, consistent with its essential role in FAT10 loading onto UBA6^31^. By acting as a stable anchor on UBA6, UBL1 allows the more flexible UBL2 domain to rotate from adenylation to thiolation, resulting in FAT10 monopolising the adenylation site.

In the case of ubiquitin, a doubly loaded UBA1~ubiquitin^Thio^ structure^28^ demonstrates that, once loaded onto the SCCH catalytic Cys, the first ubiquitin occupies a new site supported by the FCCH domain, thereby freeing the AAD for activation of the second ubiquitin molecule (Fig. 2h)^28^. In contrast to this, our structure shows that SCCH-loaded FAT10 continues to obstruct the adenylation site of the AAD (Fig. 2h). This continued blockade of the AAD by thio-linked FAT10 would prevent a ubiquitin or second FAT10 molecule from being activated, which may sustain FAT10 out-competition of ubiquitin (Fig. 2i).

Using our structural insights into FAT10 adenylation and thiolation choreography, we explored factors causing FAT10 to load more slowly than ubiquitin onto UBA6 (Fig. 1c). Adenylation requires the C-terminal Gly residue to react with ATP accessed through a dedicated channel in the AAD (Supplementary Fig. 4a). Comparing side chain positions of UBA6 residues lining this channel when bound to ubiquitin (PDB ID 9QIA)^21^ or FAT10 (UBA6-FAT10^Aden^) highlighted a notable difference in His614 at the channel entry (Supplementary Fig. 4a). When ubiquitin is bound, His614 is flipped outwards, stabilised by stacking with ubiquitin Arg42. This rotation forms a negatively charged pocket that accommodates ubiquitin tail residue Arg72, which is further coordinated by UBA6 Glu601. In the presence of FAT10, His614 is positioned inwards occluding the equivalent FAT10 residue (Tyr161) from binding to UBA6 Glu601, ultimately preventing FAT10 from burrowing as deeply into this negatively charged pocket (Supplementary Fig. 4b). In agreement with the FAT10 tail being less optimal for engaging UBA6 for adenylation, replacing the FAT10 tail with that of ubiquitin (FAT10^LRLRGG^) enhanced the overall rate of thiolation onto UBA6 to match that of ubiquitin (Supplementary Fig. 4c). Our findings suggest the sub-optimal engagement of FAT10’s tail necessitates UBL1 to stabilise UBL2 for adenylation and reveals how FAT10 achieves continued dominance of UBA6 over ubiquitin by physically blocking the AAD until transferred to an E2.

### UBA6-compatible E2s differ in ubiquitin and FAT10 activity

**Fig. 3:**
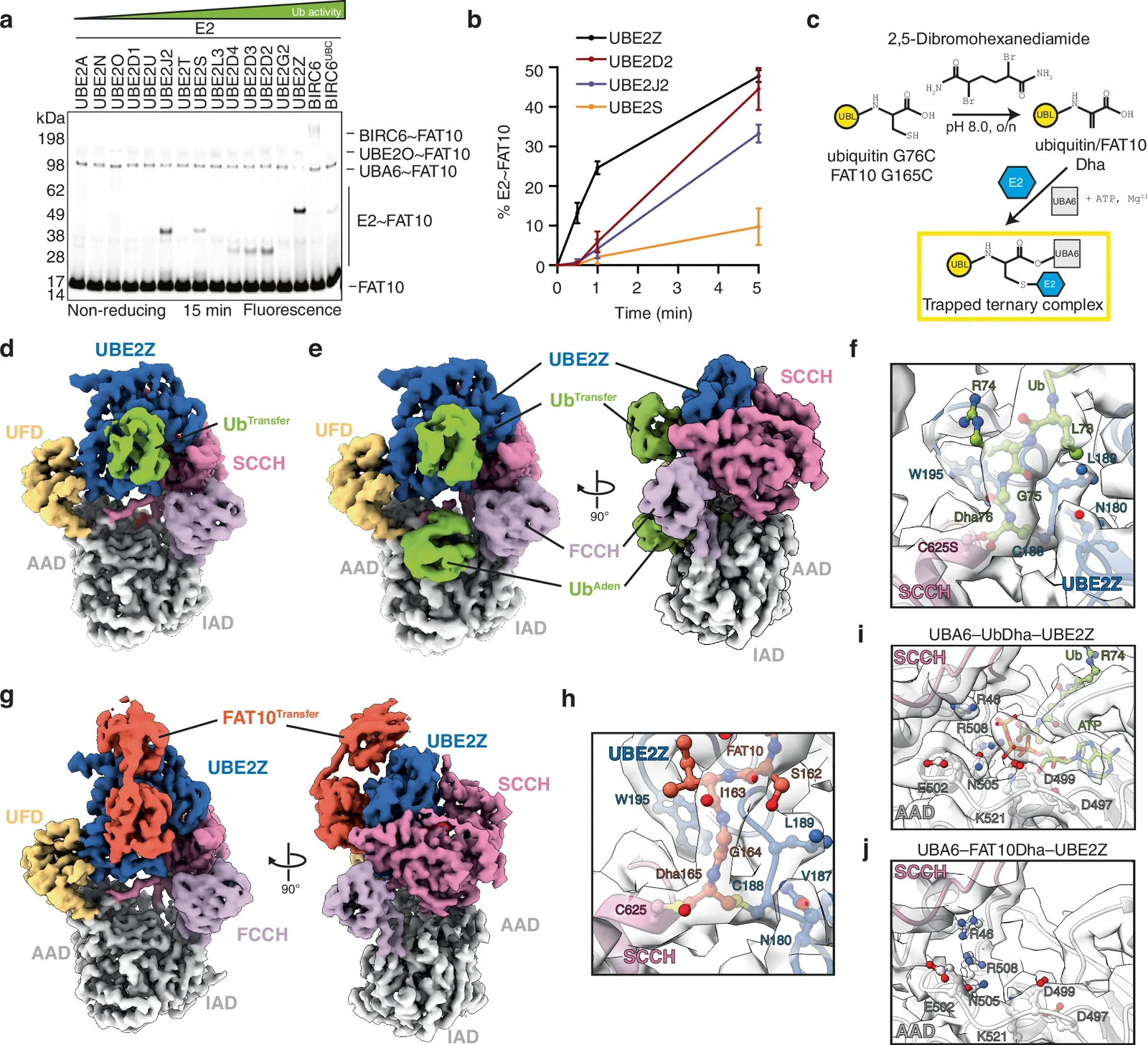
Structural visualisation of ubiquitin and FAT10 transfer to UBE2Z, the most efficient FAT10 E2. **a** A subset of the 15 UBA6-competent ubiquitin-conjugating E2 enzymes receive FAT10. Shown by in vitro FAT10 translocation assays in 5 min using Cy3-labelled FAT10. E2 enzymes are ordered by increasing ubiquitin-transfer activity, highlighting that the ability of an E2 to receive ubiquitin does not directly correlate with the ability to receive FAT10. UBC, ubiquitin-conjugating domain. Gel is representative of three independent experiments. **b** Quantifying FAT10 transfer to the most robust FAT10 E2s in multi-turnover time course in vitro FAT10 transthiolation assays reveals UBE2Z is the most efficient FAT10-active E2 enzyme. The Graph represents mean ± S.D. of three independent experiments. **c** Schematic for the generation of the FAT10Dha probe analogous to UbDha^21^ to trap Ub/FAT10 handover from UBA6 to E2s in a ternary complex that closely resembles the native transient tetrahedral intermediate. **d**, **e** Cryo-EM maps of UBA6 transferring ubiquitin to UBE2Z revealed both singly- (**d**) and doubly- (**e**) loaded states of the complex. **f** Defined three-way density was observed between UBA6 C625S, UbDha76 and UBE2Z C188, confirming ternary complex formation. **g** Cryo-EM map of UBA6 transferring FAT10 to UBE2Z resolved FAT10 in the transfer position but not in the AAD, representative of a singly-loaded state. **h** Clear three-way density was observed between UBA6 C625, FAT10Dha165 and UBE2Z C188, confirming ternary complex formation. **i**, **j** Whilst the ATP-binding pocket was occupied by either ATP or Ub-ATP in singly- and doubly-loaded ubiquitin states, respectively (**i**), no corresponding density was observed in the FAT10 transfer state. This was accompanied by partial unfolding of the ATP-binding site.

Next, we examined the functional consequences of this rigid FAT10 thiolation state on E1-E2 transthiolation activity. While several E2 enzymes have been reported to receive FAT10 from UBA6 (UBE2Z, UBE2D3, UBE2O, UBE2A, UBE2C, UBE2G2)^38,39^, we adopted an unbiased approach following our recent profiling of UBA1/UBA6 ubiquitin E2 specificity^21^ and tested all UBA6-competent ubiquitin E2s for their ability to receive FAT10 in in vitro transthiolation assays. Out of the 15 ubiquitin E2s capable of working with UBA6, six robustly received FAT10 over short and extended timeframes (5 min and 15 min, respectively): UBE2Z, UBE2J2, UBE2D2, UBE2D3, UBE2D4 and UBE2S (Fig. 3a and Supplementary Fig. 5a). Very weak transfer of FAT10 was also observed to BIRC6 and UBE2O at 15 min, consistent with earlier reports^39,40^ (Fig. 3a). Importantly, the ability of E2s to receive FAT10 did not directly correlate with their propensity to receive ubiquitin from UBA6 since robust UBA6 ubiquitin-active E2s such as UBE2L3, UBE2G2 and BIRC6 did not robustly receive FAT10 (Fig. 3a and Supplementary Fig. 5b). We then established a hierarchy of FAT10 E2 activity. Focusing on the E2s most proficient at receiving FAT10 (UBE2Z, UBE2J2, UBE2D2 and UBE2S), we quantified multi-turnover time course transthiolation assays, confirming that UBE2Z (also known as UBA6-specific E2 enzyme 1, USE1) is the best E2 at receiving FAT10 from UBA6 with ~25% loading on UBE2Z detected in 1 min compared to UBE2D2 (~8%), UBE2J2 (~5%) and UBE2S (~2%) (Fig. 3b). Importantly, the observed E2~FAT10 signal corresponds to a thioester-linked species as incubation with reducing agent DTT collapsed the E2~FAT10 adduct with loss of FAT10 signal (Supplementary Fig. 5c). Focussing on UBE2Z, a very weak signal remained at longer time points (15 min) suggesting low level formation of an isopeptide-linked UBE2Z–FAT10 species, consistent with reports that UBE2Z displays auto-FAT10ylation activity^38,41^.

### FAT10 and ubiquitin transfer use shared UBA6-UBE2Z geometry

To understand which E2 features promote FAT10 transthiolation activity, we sought to compare how UBA6 transfers ubiquitin and FAT10 to UBE2Z, the most active E2 for FAT10 (Fig. 3b). To achieve this, we trapped transfer of both FAT10 and ubiquitin from UBA6 to UBE2Z in a near-native conformation. To achieve these trapped complexes, we built on our recent strategy of using a C-terminal Dha probe to trap the transient E1-E2 transthiolation intermediate between UBA6–UbDha–BIRC6^21^. This strategy employs mutation of the UBL’s C-terminal glycine of ubiquitin/FAT10 to cysteine, followed by conversion to dehydroalanine (UbDha / FAT10Dha). Once the E2 catalytic cysteine attacks the Dha moiety, a stable ternary complex is formed, which closely resembles the transient tetrahedral intermediate formed in the transthiolation reaction (Fig. 3c, Supplementary Figs. 6a and 7a). For trapping ubiquitin, we used UBA6 with catalytic Cys625 mutated to Ser to aid stability of the conjugate without impairing UBA6~Ub loading. However, we did not detect robust loading of FAT10Dha onto UBA6^C625S^; therefore, UBA6^WT^ was used for trapping FAT10 transfer (Supplementary Fig. 7a).

Purified complexes were prepared and analysed by cryo-EM, yielding maps of UBA6–UbDha–UBE2Z and UBA6-FAT10Dha–UBE2Z at overall resolutions of ~ 3 Å (Fig. 3d–j, Supplementary Figs. 6, 7 and Table 1). Unambiguous density enabled building of the three-way UBA6–ubiquitinDha/FAT10Dha–UBE2Z covalent bonds (Fig. 3f, h). In both complexes, UBE2Z was positioned in the central E2-binding cavity of UBA6, sandwiched between the UFD and SCCH domain. This is consistent with positional arrangements observed for UBL transfer from other E1 enzymes (Supplementary Fig. 8a–f)^21,27,29,32,42–44^.

Strikingly, both UBL domains of FAT10 were clearly resolved and fully engage with UBE2Z (Fig. 3g). Additionally, FAT10 is positioned on the front face of UBE2Z, in contrast to a cryo-EM structure of UBA7 transferring ISG15 in which density for only one UBL of the transferred ISG15 molecule (UBL2) is observed and on the back face of its cognate E2, UBE2L6^45^ (Supplementary Fig. 8f). In both of our captured UBL transfer structures, the transfer ubiquitin/FAT10 moieties (Ub^Transfer^/FAT10^Transfer^) were well resolved in contrast to other E1-Ub-E2 cryo-EM structures^21,29^, suggesting a more stable E2-UBL^transfer^ association for UBE2Z compared to other ubiquitin E2s. Consistent with UBA6-Ub-BIRC6^21^ and UBA1-Ub-E2 cryo-EM structures^29^ (Supplementary Fig. 8a, d), both UBA6-Ub-UBE2Z were found in both singly- and doubly-loaded states (61% and 39% respectively) (Supplementary Fig. 6b, c).

In contrast to ubiquitin transfer, only one FAT10 moiety was resolved (FAT10^Transfer^); no classes were identified containing a second FAT10 moiety bound in the adenylation domain. Furthermore, the ATP-binding pocket in the AAD was partially ordered, and no ATP was bound, despite a molar excess of both ATP and FAT10Dha over UBA6 and UBE2Z in the reaction (Fig. 3j). The lack of bound ATP and absence of a second FAT10 post-transthiolation, despite the adenylation domain being relieved from the blockade by FAT10^transfer^, suggests that FAT10 transthiolation is coupled to the adenylation domain. Comparison of our ubiquitin transfer (Ub–AMP bound) and FAT10 transfer (no ATP-bound) cryo-EM structures did not reveal any obvious conformational changes responsible for such coupling. However, it is possible that very small differences are masked at the present resolution of our cryo-EM data, and only in much larger datasets would such changes be visible as were observed recently for ubiquitin transfer from *S.pombe* UBA1 to Ubc4^29^. Combined with only one FAT10 moiety being bound onto UBA6 during the first thiolation reaction (Fig. 2h, i), our structures suggest that in contrast to ubiquitin, UBA6 is a single passenger vehicle for FAT10: when UBA6 encounters a FAT10, that FAT10 molecule must complete the full process of activation, thiolation, transthiolation to an E2 and E2~FAT10 release before the next FAT10 or ubiquitin can be activated and transferred.

### UBE2Z engages both UBL domains of FAT10 for maximum activity

**Fig. 4:**
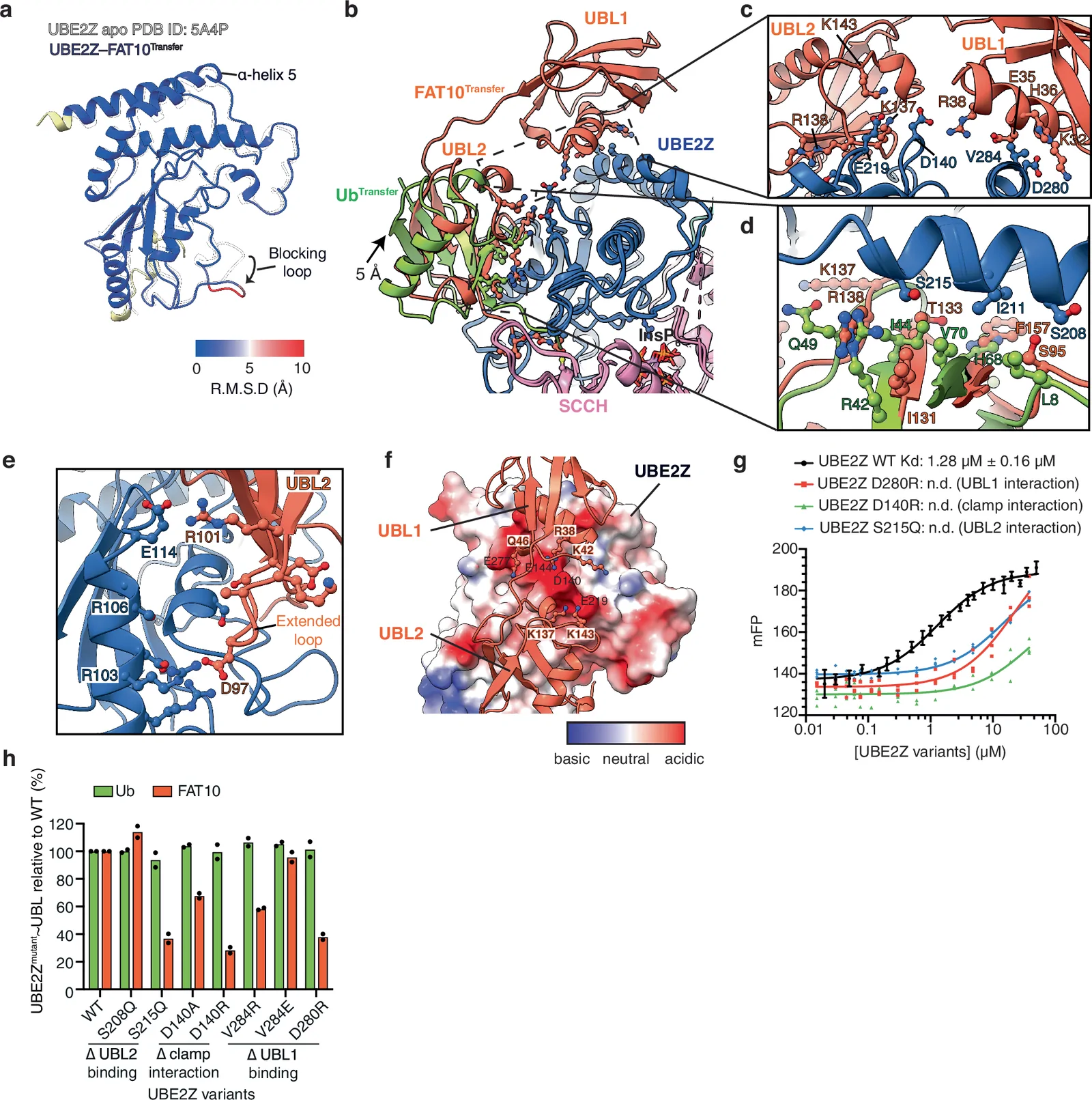
Simultaneous engagement of both FAT10 UBL1 and UBL2 domains is required for robust transthiolation to UBE2Z. **a** Structural rearrangements in UBE2Z for ubiquitin or FAT10 transfer are limited to the blocking loop, as assessed by RMSD between cartoon representations of UBE2Z–Ub/FAT10Dha and apo UBE2Z PDB ID 5A4P^46^. **b** Overlaying cartoon representations of UBA6–UbDha–UBE2Z and UBA6–FAT10Dha–UBE2Z models highlights how FAT10^UBL2^ occupies a similar position to ubiquitin, with a small upwards translation of 5 Å owing to anchoring by UBL1. **c** UBE2Z engages FAT10^UBL1^ through a helix in its C-terminal extension to the UBC domain, defining a distal-binding site. **d** UBE2Z utilises the same residues to engage non-analogous residues between FAT10^UBL2^ and ubiquitin. **e** UBE2Z engages with an extended loop in FAT10^UBL2^. **f** UBL1 and UBL2 clamp around a negatively charged ridge on UBE2Z visualised by electrostatic surface representation of UBE2Z receiving FAT10. **g** Engagement with both UBL1 and UBL2 results in a tight affinity interaction between UBE2Z and FAT10 as measured by fluorescence polarisation. The Graph represents one (mutants) or three (WT) independent experiments, each performed in technical triplicate. The WT data are presented as mean ± S.D. **h** Interaction with UBL1 and UBL2 is required for optimal transfer of FAT10 to UBE2Z in single turnover in vitro transthiolation assays at 5 min. The graph represents the mean of two independent experiments.

Our structures reveal that UBE2Z adopts strikingly similar conformations irrespective of the UBL being transferred (RMSD of 0.36 Å). Further to this, the blocking loop in UBE2Z that occludes the catalytic Cys188^46^ undergoes the same conformational change to accommodate the tail of both ubiquitin and FAT10 (Fig. 4a). Furthermore, UBE2Z uses an almost identical interface to engage with FAT10^UBL2^ and ubiquitin^transfer^, with differences between surface exposed residues on the UBLs being accommodated through a 5 Å translation of FAT10^UBL2^ relative to ubiquitin (Fig. 4b). UBE2Z residues Ile211, Ser215 engage ubiquitin through the canonical ubiquitin Ile44 patch (including Ile44 and Val70) and engage differently positioned hydrophobic residues (Ile131 and Phe157) in FAT10^UBL2^ (Fig. 4b, d). In addition, FAT10^UBL2^ also contains an extended loop between β5-β6 (residues 94-100) (Supplementary Fig. 1a) that positions Asp97 to form electrostatic contacts to Arg103 and Arg106 of UBE2Z and forms electrostatic interactions between residues Arg101 of FAT10^UBL2^ and Glu114 of UBE2Z (Fig. 4e). Finally, Glu219 of UBE2Z interacts with analogously positioned positively charged side chains in ubiquitin (Gln49) and FAT10^UBL2^ (Lys137) (Fig. 4c).

FAT10^UBL2^ is rigidly anchored by UBL1 moulding over α-helix 5 of UBE2Z, particularly residues Asp280 and Val284 (Fig. 4c), forming a distal binding site. Further to this, UBL1 Arg38 and UBL2 Lys137, Lys143 function in concert to clamp a negatively charged ridge on UBE2Z (including Asp140 and Glu219), enabling FAT10 to lock onto UBE2Z at two distinct interfaces (Fig. 4c, f).

Given the apparent stability of both UBL domains in our cryo-EM map, we asked if this manifests in a measurable E2-UBL affinity. Indeed, fluorescence polarisation assays revealed a tight affinity of 1.28 μM ± 0.16 μM (Fig. 4g), which is markedly higher than other E2-UBL interactions^47^. Introducing single point mutations in UBE2Z at the observed interfaces with UBL1 (Asp280), UBL1-UBL2 clamp (Asp140) or UBL2 (Ser215) all compromised binding to UBE2Z (Fig. 4g) and impaired transfer of FAT10 without affecting ubiquitin transfer (Fig. 4h). This suggests that complete engagement of FAT10 by the E2 is required for optimal FAT10 transfer.

Identification of a UBE2Z mutation in the FAT10^UBL2^ binding site (S215Q) that prevents FAT10 from loading whilst not affecting ubiquitin was surprising, given that UBE2Z engages both through the same interface (Fig. 4b, d). We rationalise this difference through the extension of UBE2Z Ser215 into a larger side chain, which now clashes with FAT10 Arg138 held across the FAT10-UBE2Z interface. While ubiquitin does interact with UBE2Z Ser215, mediated by ubiquitin Arg42 from an adjacent strand reaching within hydrogen-bond distance (Fig. 4d), the equivalent residue of FAT10 Arg138 is ubiquitin Gln49 that can likely hydrogen-bond to the mutated UBE2Z site, retaining ubiquitin binding capability (Fig. 4d). Further probing the importance of the UBE2Z-UBL2 interface highlighted that certain mutations can be tolerated. For example, S208Q results in a minor increase in FAT10 transfer (Fig. 4h) by potentially promoting an interaction with FAT10 Ser95, whilst remaining compatible with ubiquitin Leu8 (Fig. 4d). This highlights the structural plasticity occurring within the E2-UBL2 binding site.

Together, our structural and biochemical data highlight that both FAT10 UBL domains and the linker are not only required for loading onto UBA6 (Fig. 2) but are also critical for transfer to UBE2Z. This is distinct from ISG15, as only UBL2 engages with the E1 and E2^45,48^ (Supplementary Fig. 8f). This suggests the functional requirement for UBL1 is a FAT10-specific phenomenon, providing an additional interface for recognition by the FAT10 machinery.

### E2 interaction with FAT10^UBL2^ promotes transfer

**Fig. 5:**
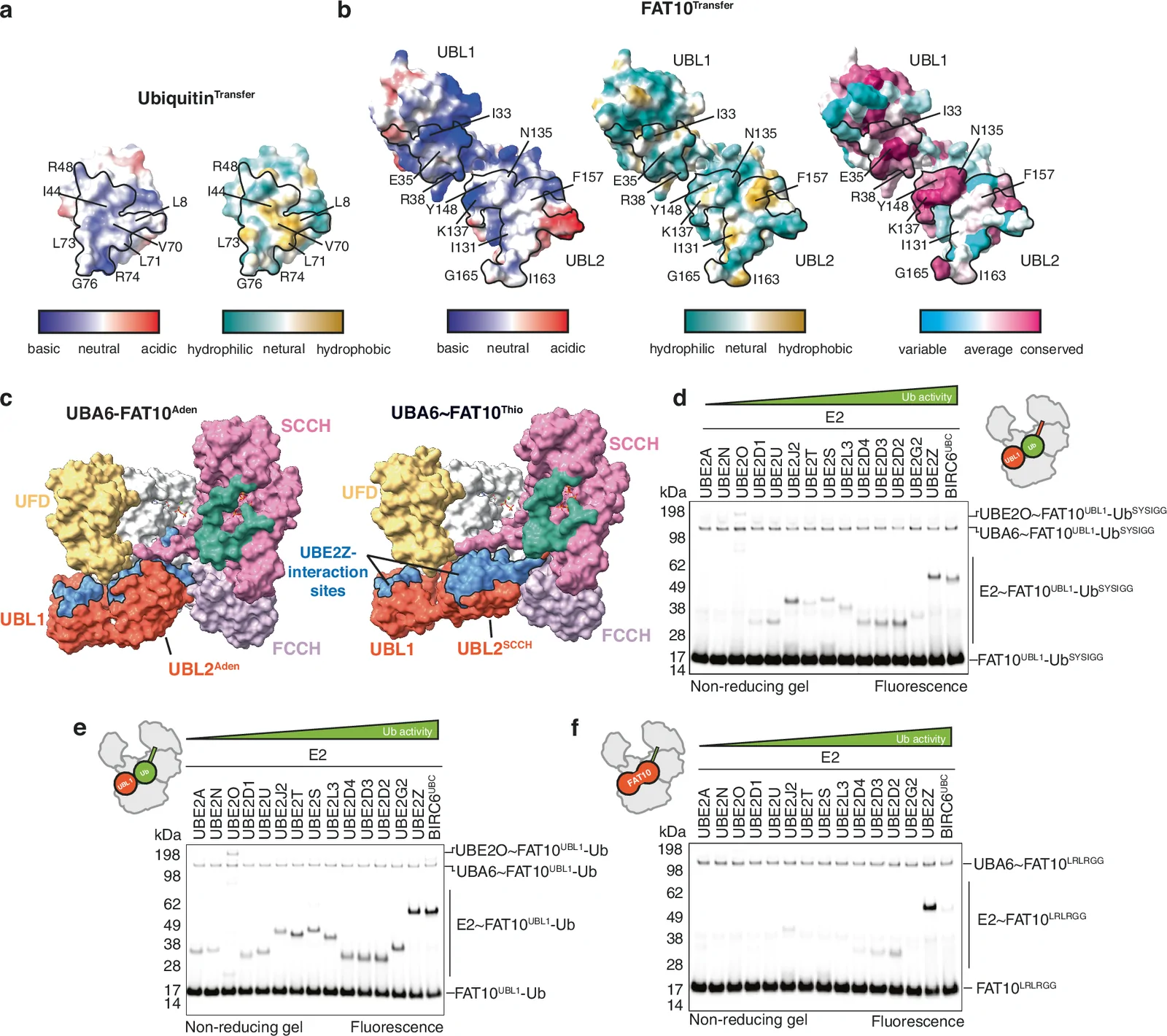
E2 compatibility with the surface of FAT10UBL2 domain is a driver for FAT10 transfer. **a**, **b** Surface representation of ubiquitin (**a**) and FAT10 (**b**) highlights that the FAT10 surface engaged by UBE2Z is more hydrophilic than the classical ubiquitin Ile44 patch used for E2-binding. UBE2Z engagement is outlined in black. **c** FAT10 surfaces engaged by UBE2Z become exposed to the UBA6 E2-binding cavity when FAT10 transitions from the adenylation site to the post-thiolation state. **d**–**f** E2 enzymes receive a FAT10 with either UBL2 substituted for ubiquitin but retaining the FAT10 C-terminal tail (**d**) or replacing the entire UBL2 region with ubiquitin (**e**). However, they display lower activity with a FAT10 in which only the C-terminal tail is replaced with the C-terminal tail of ubiquitin (**f**). Determined by multi-turnover in vitro transthiolation assays using Cy3-labelled FAT10-ubiquitin hybrids over 5 min. E2 enzymes are ordered by increasing ubiquitin-transfer activity. UBC ubiquitin-conjugating domain. Gels are representative of three independent experiments.

Our structure of UBA6 transferring FAT10 to UBE2Z defines an E2-interacting region on FAT10 that covers approximately 1404 Å^2^, representing 14% of its total surface area. UBE2Z engages ubiquitin at the canonical Ile44 hydrophobic patch, whilst the FAT10 engaged surface is mostly hydrophilic but includes two hydrophobic residues, Ile131 and Phe157, that are offset from the ubiquitin Ile44 patch (Fig. 5a, b). Additionally, as described earlier, the UBE2Z-engaged interface of FAT10 extends to UBL1 with the conserved UBL1 residues Glu35 and Arg38 (Fig. 5b and Supplementary Fig. 1a) interacting with UBE2Z at the distal binding site (Fig. 4f).

Our UBA6 ~ FAT10 structures show that FAT10, once loaded onto the UBA6 SCCH domain, remains bound across the adenylation site, which limits the interfaces available for E2 engagement (Fig. 2a). We assessed whether the interfaces engaged by UBE2Z during E1-E2 transfer would be accessible during FAT10 loading onto UBA6. Mapping of these interfaces onto our UBA6-FAT10^Aden^ structure (Fig. 2a) showed the UBE2Z-interacting surfaces are mutually exclusive from those engaged by UBA6 but are initially occluded from an incoming E2 (Fig. 5c, left). However, the rotation of UBL2 required for FAT10 loading onto the SCCH domain (Fig. 2d) concomitantly exposes the UBL2 interface engaged by UBE2Z (Fig. 5c, right). The UBL1 interface continues to point towards the UFD and is relatively obstructed from the E2-binding cavity in UBA6~FAT10^Thio^. Thus, we propose a two-stage process by which initial UBE2Z engagement with UBL2 destabilises FAT10 held in the adenylation position, ultimately freeing UBL1 to bind to the distal UBE2Z site.

This led us to assess whether engagement with UBL2 is a determinant for E2s to receive FAT10. In support of this, exchanging FAT10^UBL2^ to ubiquitin but retaining the FAT10 C-terminal tail residues enabled transfer to most UBA6 ubiquitin-active E2s (Fig. 5d), suggesting that UBL2 contributes to E2 selectivity. Substituting UBL2 in its entirety for ubiquitin resulted in robust transfer to all UBA6 ubiquitin-active E2s, closely mirroring ubiquitin transfer (Fig. 5e and Supplementary Fig. 5b). This suggests that the FAT10 C-terminal tail contributes to E2 compatibility, but the contribution is minor. Consistent with this, exchanging only the FAT10 C-terminal tail with that of ubiquitin failed to promote transfer to E2s that normally do not receive FAT10 (Fig. 5f). Together, these findings suggest a hierarchy of interactions for selectivity, with the UBL2-E2 interaction interface being the primary driver.

### Efficiency of E2 recruitment to UBA6 impacts FAT10 transfer

Our UBL1-ubiquitin hybrids revealed that a compatible UBL2 binding interface is required for E2 FAT10 activity (Fig. 5d, e). Curiously, the UBE2D family, which share 95–98% homology (Supplementary Fig. 9a), stood out as displaying varying degrees of FAT10 activity with UBE2D2 most robustly receiving FAT10 (Fig. 3a). Modelling all UBE2D members receiving FAT10 suggests no obvious differences in their UBL2 interfaces (Supplementary Fig. 9b), indicating that a factor other than UBL2 binding contributes to their ability to receive FAT10.

**Fig. 6:**
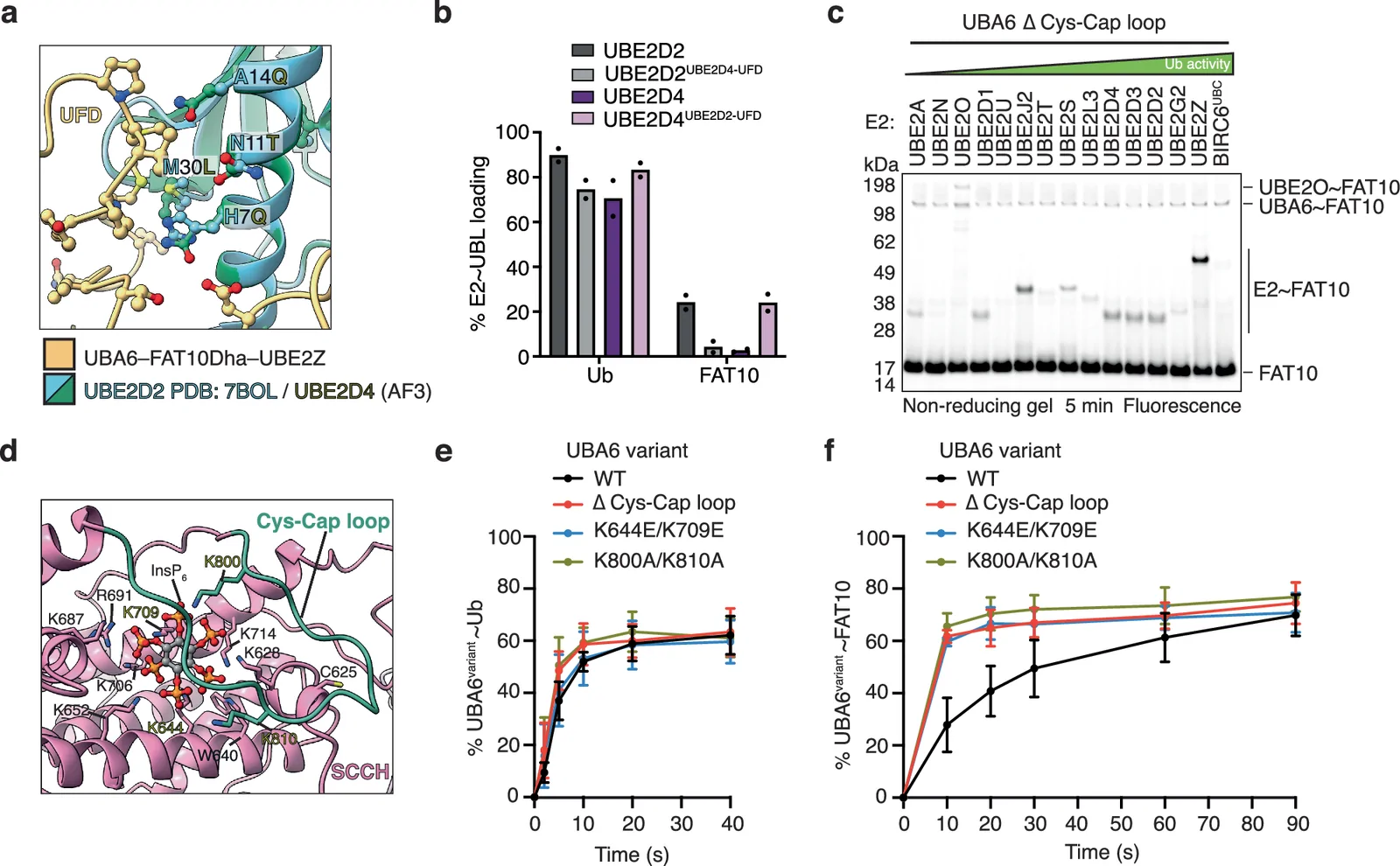
Mechanisms of E2 recruitment to UBA6 affect level of FAT10 transfer. **a** Modeling UBE2D2 PDB ID 7BOL^77^ and UBE2D4 (AF3^78^ model) bound to UBA6, based on our UBA6–FAT10Dha–UBE2Z structure, highlights sequence differences in the UFD-interacting region. **b** substitution of UBE2D2 UFD-interacting residues into UBE2D4 boosts FAT10 transfer assessed through multi-turnover in vitro transthiolation assays in 5 min. The graph represents the mean of two independent experiments. **c** In comparison to wild-type UBA6 (Fig. 3a), removal of the Cys-Cap loop in UBA6 promotes Cy3-FAT10 transfer to E2s assessed through multi-turnover in vitro transthiolation assays in 5 min. Gel is representative of three independent experiments. **d** Residues of UBA6 SCCH and Cys-Cap loop (green) that engage InsP_6_. **e**, **f** Removal of InsP_6_ binding (K644E/K709E), Cys-Cap loop engagement with InsP_6_ (K800A/K810A), or the Cys-Cap loop entirely, dramatically enhances FAT10 loading onto UBA6 while having a minor increase in ubiquitin loading. The Graph represents mean ± S.D. of three independent experiments.

Variations between the UBE2D family centre around the UFD and SCCH interacting regions (Fig. 6a and Supplementary Fig. 9a, b), key interfaces, which determine E2 recruitment to UBA6 over UBA1^21^. Exchanging the UFD-interacting region of UBE2D4 with that of UBE2D2 (Supplementary Fig. 9a, dashed line) dramatically increased FAT10 transfer (Fig. 6b). Conversely, inserting the UBE2D4 UFD-interacting sequence into UBE2D2, decreased FAT10 transfer, suggesting that the ability of the UBE2D family to be recruited to UBA6 has a bearing on FAT10 transfer (Fig. 6b). Curiously, ubiquitin transfer was only marginally affected, suggesting FAT10 is more sensitive to weakening of UBA6 - E2 interactions (Fig. 6b).

We extended our analyses further to test whether improved recruitment of all UBA6-compatible E2s promotes FAT10 transfer. We recently identified that the UBA6 Cys-Cap loop dampens BIRC6 recruitment to UBA6, with its removal resulting in an increased E1-E2 affinity^21^. Using this as a general way to assist E2 recruitment, we observed increased levels of FAT10 transfer to UBE2A and UBE2O, and detected low-level transfer to UBE2L3 and UBE2G2 (Fig. 6c). Consistent with our finding that the UBE2D family recruitment to UBA6 affects their FAT10 reactivity, loss of the Cys-Cap loop equalised FAT10 transfer to the four UBE2D family members. No additional E2s began to robustly receive FAT10 to the level of UBE2Z, supporting that compatibility with UBL2 is a main governor. However, our findings reveal an underlying requirement for a favourable recruitment mechanism to UBA6.

### E2 coordination of InsP_6_ regulates FAT10 tansthiolation

**Fig. 7:**
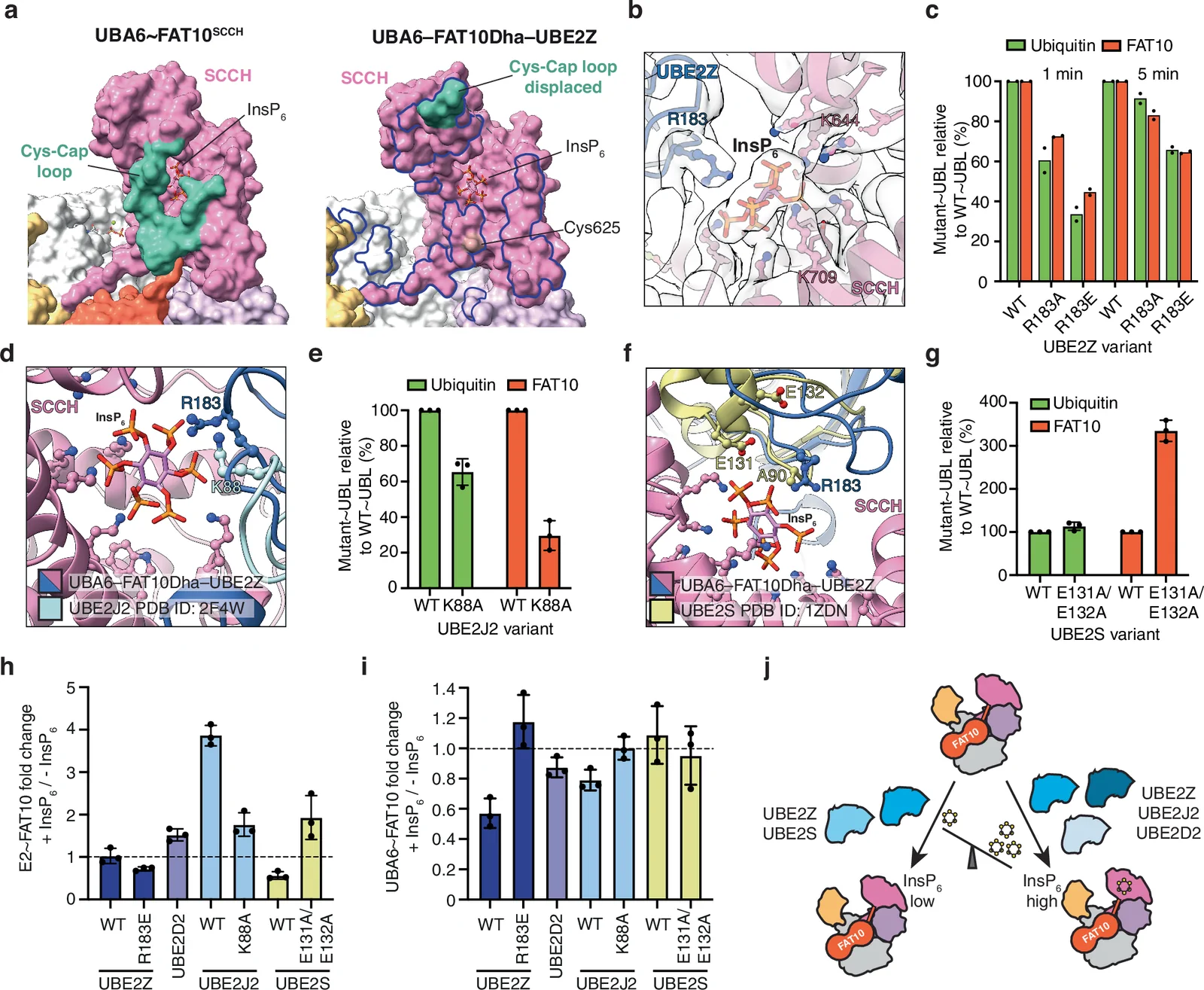
The metabolite InsP_6_ modulates FAT10 transfer to E2 enzymes. **a** UBE2Z recruitment to UBA6~FAT10 displaces the Cys-Cap loop, unmasking the catalytic Cys~FAT10 for transfer to UBE2Z. This displacement exposes SCCH-bound InsP_6_ to the E2-binding cavity where UBE2Z is positioned. Residues of the UBA6 SCCH domain that interact with UBE2Z are outlined in blue. **b** R183 from UBE2Z engages InsP_6_ bound in the SCCH pocket. **c** Loss of InsP_6_ coordination reduces ubiquitin and FAT10 transfer to UBE2Z, measured by single turnover in vitro transthiolation assays. The graph represents the mean of two independent experiments. **d** Modelling UBE2J2 bound to UBA6 identifies a positively charged residue at an analogous position to UBE2Z R183 that could coordinate InsP_6_. **e** Loss of proposed UBE2J2 coordination of InsP_6_ decreases ubiquitin and FAT10 transfer measured by multi-turnover in vitro transthiolation assays in 5 min. The graph represents the mean ± S.D. of three independent experiments. **f** Modelling UBE2S (PDB ID:1ZDN^79^) bound to UBA6 highlights two consecutive negatively charged residues (E131 and E132) that may repel InsP_6_ bound in the SCCH. **g** Removal of potential InsP_6_ repulsive residues in UBE2S promotes FAT10 transfer in multi-turnover in vitro transthiolation assays in 5 min. Ubiquitin transfer was not notably affected. The graph represents the mean ± S.D. of three independent experiments. **h**, **i** Addition of exogenous InsP_6_ to UBA6 purified from *E. coli* lacking inositol phosphates promotes FAT10 transfer to UBE2Z, UBE2D2 and UBE2J2 but limits transfer to UBE2S in single-turnover in vitro transthiolation assays. Altering E2 compatibility with InsP_6_ results in corresponding effects on FAT10 transfer. Exogenous InsP_6_ inhibits transfer to InsP_6_-repelling mutant UBE2Z R183E and no longer markedly improves FAT10 transfer to InsP_6_-coordination-defective UBE2J2 K88A. Conversely, InsP_6_ promotes FAT10 transfer to InsP_6_-compatible UBE2S E131A/E132A. Graphs represent mean ± S.D. Cy3-FAT10 transferred onto E2 enzymes (**h**) or remaining on UBA6 (**i**) in 1 min (UBE2Z) or 15 min (UBE2D2, UBE2J2, UBE2S) from three independent experiments. **j** Differential co-ordination of InsP_6_ by FAT10-compatible E2s offers an opportunity for fine-tuning FAT10-controlled pathways in response to fluctuating cellular InsP_6_ levels.

In our UBA6~FAT10^Thio^ structure, we observed the Cys-Cap loop resting over the SCCH domain catalytic cysteine, even in the presence of thio-linked FAT10 (Fig. 2a, d, e). A previous study noted that Cys-Cap loop stabilisation, enhanced by the metabolite InsP_6_ bound in the SCCH domain, impedes UBL loading^37^. InsP_6_ is a highly abundant eukaryotic cellular metabolite, thus was present in our UBA6 structures obtained using insect cell (Sf9)-derived recombinant UBA6, and was engaged to the Cys-Cap loop in the absence of E2 (Fig. 6d). Removing InsP_6_ binding (K644E/K709E), disrupting InsP_6_-Cys-Cap loop interactions (K800A/K810A), or removing the Cys-Cap loop entirely (replacing residues 799-816 with a GGSGG linker) boosts FAT10 loading whilst having a lesser effect on ubiquitin loading (Fig. 6e, f), aligning with previous findings^37^. In our UBL transfer structures, the Cys-Cap loop is displaced when UBE2Z is bound, exposing the InsP_6_ molecule held in the SCCH domain (Fig. 7a). Strikingly, we observed UBE2Z coordinating this InsP_6_ through Arg183, which is positioned identically in both FAT10Dha and UbDha-linked structures (Fig. 7b). Mutation of Arg183 to either remove the charge (R183A) or invert the charge (R183E) decreased both ubiquitin and FAT10 transfer, showing this interaction with UBA6 is functionally important (Fig. 7c).

We then investigated if InsP_6_ co-ordination is a characteristic of other FAT10-active E2s. Indeed, aligning other FAT10-compatible E2s with UBE2Z highlighted a positively charged residue in UBE2J2 at an analogous position (Lys88) (Fig. 7d). Mutating Lys88 of UBE2J2 to remove the charge also reduced ubiquitin and FAT10 transfer (Fig. 7e). Aligning UBE2Z with UBE2S highlighted two consecutive negatively charged residues facing the InsP_6_ pocket (Glu131, Glu132) which may be unfavourable for InsP_6_ co-ordination, limiting UBL transfer (Fig. 7f). Consistent with this, removal of this negative charge noticeably enhanced FAT10 transfer (Fig. 7g). Notably, altering UBA6-E2 interfaces either via the UFD (Fig. 6a, b) or InsP_6_ (Fig. 7e, f) affected FAT10 transfer more dramatically than ubiquitin highlighting the requirement of an optimal UBA6-E2 interaction for FAT10 transfer.

To further test whether E2-mediated co-ordination of InsP₆ within the UBA6 SCCH domain influences FAT10 transfer, we examined FAT10 transfer following exogenous InsP₆ addition. For this, we used UBA6 purified from *E. coli*, an expression system lacking inositol phosphates, and performed single turnover discharge assays (Fig. 7h, i), quantifying the levels of FAT10 discharged onto the E2 and remaining on UBA6. Addition of InsP_6_ markedly promoted loading onto UBE2J2 concurrent with decreased UBA6~FAT10, which was not observed upon loss of the InsP_6_ co-ordinating residue, K88A. Only a minor increase in transfer to UBE2D2 was observed, supporting that efficient FAT10 transfer to the UBE2D family depends largely on interactions with the UBA6 UFD (Fig. 6a, b and Supplementary Fig. 9b). In agreement with a sub-optimal interaction between UBE2S and InsP_6_, exogenous InsP_6_ reduced FAT10 transfer to wild-type UBE2S but enhanced FAT10 transfer to UBE2S E131A/E132A, in which the predicted repulsive interactions are removed. Focussing on UBE2Z, addition of InsP_6_ enhanced FAT10 transfer, as a ~two-fold decrease in UBA6~FAT10 was observed. This was not mirrored in FAT10 loading onto wild-type UBE2Z, likely due to spontaneous hydrolysis of the UBE2Z~FAT10 thioester bond. However, addition of InsP_6_ reduced FAT10 transfer to UBE2Z R183E and increased UBA6~FAT10 levels, together supporting Arg183-mediated co-ordination of InsP_6_, which promotes FAT10 transfer from UBA6 to UBE2Z (Fig. 7h,i). Overall, these findings identify InsP_6_ as a direct regulator of FAT10 transfer.

## Discussion

Our findings reveal the complete process of how FAT10 is loaded onto UBA6 and subsequently transferred onto E2-conjugating enzymes, the first critical stages of the FAT10 conjugation pathway. Mechanistically, we find that FAT10 is a sum of all parts, requiring the entirety of its flexibly linked double UBL structure for function, from gaining access to UBA6 over the more abundant ubiquitin, to optimal recognition and transfer to E2 conjugating enzymes. Furthermore, we define the E2s that are capable of robustly receiving FAT10 and identify the principal governors of E2 FAT10 compatibility as a combination of UBL2 recognition and mode of interaction with UBA6, identifying a direct role for the metabolite InsP_6_ within the catalytic domain. By identifying distinct FAT10-compatible E2s, we broaden the horizon of potential FAT10 E3 ligases and FAT10 substrates, which is required to ultimately illuminate the role of FAT10-controlled cellular pathways.

### FAT10 loading cycle

UBA6 is an unusual E1-activating enzyme as it activates two different UBLs. Given the abundance of cellular ubiquitin, FAT10 must employ mechanisms to gain access to UBA6. We identify a competitive relationship between FAT10 and ubiquitin, whereby FAT10 binds tighter to UBA6, gaining priority, consistent with earlier reports^23^. The mode of FAT10 binding has functional consequences for UBA6 dual UBL activity, as we observe that FAT10 achieves dominance over UBA6 through UBL1 anchoring FAT10 during adenylation and remaining bound post-thiolation. In contrast, ubiquitin SCCH-loading breaks its weak intermolecular interactions to the AAD and forms new interactions to the FCCH domain, freeing the AAD for a second incoming ubiquitin (Supplementary Fig. 10)^28^. This FAT10 AAD blockade guarantees ubiquitin does not become activated until transfer of FAT10 onto an E2 is complete, separating the local signals of FAT10 and ubiquitin even at the expense of allowing a second FAT10 to become activated. The requirement of FAT10 UBL1 for adenylation through to post-thiolation contrasts with ISG15, where UBL1 is highly flexible during adenylation^45,48^. This is potentially reflected by ISG15 possessing the ubiquitin C-terminal tail sequence (LRLRGG), thus likely optimally engaging with the ATP-binding pocket, coupled with ISG15 being activated by a dedicated E1, UBA7, negating the need for a competition mechanism.

### Rationale for solo FAT10 cycling?

Our cryo-EM data point towards FAT10 enforcing a single loading and transfer mechanism onto UBA6, resulting in only one FAT10 molecule progressing at a time; only once the E2~FAT10 conjugate is released can the cycle begin anew. This low processivity indicates FAT10 transfer is a carefully controlled process, which may be reflected in the potency of the FAT10 degradation signal in comparison to ubiquitin: conjugation of a single FAT10 molecule onto a substrate is sufficient for recognition by NUB1L and subsequent degradation by the 26S proteasome^7^. This contrasts with ubiquitin, for which, with the exception of short polypeptides that can be degraded through a single ubiquitin attachment^49^, a minimum of four ubiquitin molecules as a Lys48-linked tetra ubiquitin chain are required for degradation of most substrates^50^.

### Governance of FAT10 transfer to E2 conjugating enzymes

The FAT10 loading mechanism also has consequences for which UBA6-compatible E2s can receive FAT10. We profiled all known 15 UBA6-compatible E2 ubiquitin-conjugating enzymes in vitro, building upon previous studies^38,39^, and we identified six E2s that robustly receive FAT10: UBE2Z, UBE2D2, UBE2D3, UBE2D4, UBE2J2 and UBE2S. Although only a small subset of E2s receive FAT10 from UBA6, the E2s in question operate within distinct cellular pathways, suggesting potential roles of FAT10 in these pathways too. For example, whilst UBE2D2 and UBE2D3 regulate multiple pathways through promiscuity with E3 ubiquitin ligases^51^, and UBE2Z functions are unclear, UBE2J2 is ER-membrane associated and involved in ER protein quality control^52,53^ and sterol biosynthesis^54,55^, and UBE2S has roles within cell-cycle regulation^56–58^ and DNA damage response^59^, pointing to FAT10-regulation within these pathways.

The predominant E2 for receiving FAT10 is UBE2Z, which has been identified in other studies^38,46^. Our structural trapping of FAT10 transfer from UBA6 onto UBE2Z explains how UBE2Z is so adept at receiving FAT10 through its simultaneous engagement of both UBL domains, resulting in an unusually high affinity for an E2-UBL pair; for example, a Kd of ~300 μM was determined between UbcH5c (yeast orthologue of UBE2D3) and ubiquitin^47^ compared to the Kd of ~1 μM for UBE2Z-FAT10. During our manuscript review process, a complementary study investigating UBA6 transfer of FAT10 reported the cryo-EM structure of UBA6–UBE2Z–FAT10. There, density corresponding to UBL1 of FAT10^Transfer^ was not observed^60^ possibly attributable to different trapping strategies. In Nayak et al., FAT10 was covalently linked onto a Lys in a mutant UBE2Z rather than the catalytic Cys, likely altering UBL2 positioning and hence preventing a favourable UBL1 interaction.

The pathways UBE2Z controls are unclear. However, our structural analyses identify point mutations that decouple UBE2Z transfer of FAT10 from ubiquitin, now enabling a direct study of the roles of UBE2Z-mediated FAT10 transfer. Visualising FAT10 transfer enabled us to define the E2-binding surface on FAT10, which, importantly, is presented to incoming E2s when FAT10 is loaded onto the catalytic Cys of UBA6 (Fig. 5c). This interface is more hydrophilic than the corresponding E2-binding interface on ubiquitin, and we identify that E2 compatibility with this UBL2 interface governs the ability of FAT10 transfer.

A striking feature of our ubiquitin/FAT10 transfer-trapped structures is that UBE2Z directly engages InsP_6_ exposed in the UBA6 SCCH pocket. This interaction is functionally important for optimal transfer of both ubiquitin and FAT10 to UBE2Z, and mechanistically explains our earlier finding that UBE2Z’s exclusive activity with UBA6 is driven by the SCCH domain^21^. Our structures now reveal that UBE2Z achieves this through binding InsP_6_, which is not present in UBA1. InsP_6_ has been previously reported to hinder ubiquitin and FAT10 loading onto UBA6^37^. However, while we observe this effect in UBA6~ubiquitin/FAT10 thiolation reactions, our findings now describe a complex regulatory role of InsP_6_ in transthiolation to E2s. We now propose that InsP_6_ co-ordination is a regulatory feature across FAT10-compatible E2s. Positive regulation by InsP_6_ is evident for UBE2Z, UBE2J2 and UBE2D2 (Fig. 7h–j) whilst limiting FAT10 transfer to UBE2S. Modulation could be achieved through altered affinity to UBA6, thus acting as a molecular glue as observed in other ubiquitin pathways^61–64^, and/or through more optimal positioning of the E2 for transthiolation. Delineating such mechanisms requires further investigation. Taken together, our data offer a model whereby variation in InsP_6_ levels dictates which cellular pathways are regulated by FAT10.

With our findings explaining the mechanistic basis for how FAT10 gains supremacy over ubiquitin for activation by UBA6, it will be important to identify FAT10-regulated substrates and processes at the endogenous level rather than through overexpression, which risks forcing FAT10 flux through non-cognate FAT10-compatible E2s, ultimately onto non-physiological substrates. A greater understanding of the substrates and cellular processes regulated by FAT10 will illuminate how the cell uses two distinct UBL-conjugation pathways for proteasome-mediated degradation.

## Methods

### Protein purification

UBA6 WT and variants, BIRC6 and UBE2O were cloned into a modified pACE-Bac vector containing an N-terminal Twin-Strep™-mScarlet tag with a 3C protease site and expressed in Sf9 cells (ThermoFisher Scientific Cat. No. 11496015), as described previously^21,40^. Briefly, 1 L Sf9 pellets were lysed by gentle sonication in 20 mM HEPES, pH 8.5, 200 mM NaCl, 4 mM DTT, 10% (v/v) glycerol supplemented with 0.5% (v/v) Tween-20, Complete protease inhibitor tablets (Roche) and DNase (SIGMA). Clarified lysates were applied to Streptactin 4-Flow (IBA) resin and eluted with 3C protease. Proteins were further purified by ion exchange chromatography (Resource Q, Cytiva) and size exclusion chromatography (Superdex 200, Cytiva) in 20 mM HEPES, pH 7.5, 200 mM NaCl, 4 mM DTT. BIRC6^UBC^ domain (4498–4820) was expressed in pOPIN S vector, and all remaining E2 conjugating enzymes (WT or mutants) were cloned into pOPIN B vector as full-length constructs, except for UB2E2J2 that lacked the transmembrane domain (1–230) and UBE2U (1–150), and expressed in Rosetta (DE3) *E. coli*. Purifications of E2 enzymes were performed as previously described^21^. In brief, *E. coli* pellets were lysed in 20 mM Tris, pH 7.5–8.5, 500 mM NaCl, 20 mM imidazole, 2 mM β-mercaptoethanol, supplemented with lysozyme, DNase, 2 μM pepstatin, 4 μM leupeptin and 1 mM PMSF and clarified lysates were applied to a Nickel Hi-Trap column (Cytiva). Recombinant proteins were eluted by 20 mM Tris, pH 7.5–8.5, 500 mM NaCl, 500 mM imidazole, 2 mM β-mercaptoethanol, followed by incubation with 3C protease to remove the N-terminal His_6_ tag. For some E2s, further purification was achieved by anion exchange chromatography - UBE2A, UBE2N, UBE2U, UBE2G2, UBE2Z, BIRC6^UBC^ (Resource Q, Cytiva) - or cation exchange chromatography - UBE2S, UBE2L3 (Resource S, Cytiva) – followed by size exclusion chromatography (Superdex 75, Cytiva) in 20 mM HEPES pH 7.5, 200 mM NaCl, 4 mM DTT. For the UBE2D family, UBE2J2 and UBE2T, an ion exchange chromatography purification step was not employed. FAT10 without cysteines (FAT10^ΔCys^), FAT10^ΔCys G165C^ and FAT10 UBL1 (1-84) were cloned into the pOPIN B vector, and FAT10 UBL2 (84-165) was cloned into the pOPIN S vector and were all expressed in Rosetta (DE3) *E. coli*. FAT10 constructs were purified as for E2 enzymes, and full-length constructs were purified by cation exchange chromatography (Resource S, Cytiva) in 20 mM MES pH 6.5, 20 mM NaCl, 4 mM DTT and eluted against a linear gradient of 1 M NaCl followed by size exclusion chromatography (Superdex 75, Cytiva) in 20 mM HEPES pH 7.5, 200 mM NaCl, 4 mM DTT. Ion exchange chromatography was not performed for individual UBL domains. Ubiquitin^WT^ and ubiquitin^G76C^ were cloned into a modified pOPIN B vector with no N-terminal tag and were expressed in Rosetta (DE3) *E. coli* and purified as described previously^65^. In brief, purification was achieved by addition of perchloric acid to pH 4.5, followed by cation exchange chromatography in 50 mM NaOAc, pH 4.5, 20 mM NaCl (SP-column, Cytiva) eluted against a linear gradient of 1 M NaCl followed by size exclusion chromatography (Superdex 75, Cytiva) in 20 mM HEPES, pH 7.5, 200 mM NaCl. For purification of ubiquitin^G76C^, 4 mM DTT was added to the buffers. For purification of UBA6 without co-purification of InsP_6_, UBA6 (residues 32-1052) was cloned into the pOPIN S vector and expressed in Rosetta (DE3) *E. coli* as described in^37^. Briefly, cultures were cooled to 4 °C and incubated with 1.5% (v/v) ethanol followed by induction with 200 μM IPTG at 18 °C overnight. Pellets were lysed and applied to a Nickel Hi-Trap column as described above for E2 enzymes. N-terminal His_6_-SUMO tag was removed by incubation with SENP1, followed by anion exchange chromatography (Resource Q, Cytiva) and size exclusion chromatography (Superdex 200, Cytiva) in 20 mM HEPES, pH 7.5, 200 mM NaCl, 4 mM DTT, followed by a second round of anion exchange chromatography (Mono Q, Cytiva) and buffer exchanging into 20 mM HEPES, pH 7.5, 200 mM NaCl, 4 mM DTT. All concentrated proteins were flash frozen and stored in single-use aliquots at −75 °C.

### In vitro transthiolation assays

For multi-turnover transthiolation assays, E1s, E2s and UBLs were mixed at respective final concentrations of 0.5 µM, 3 µM and 5 µM in transthiolation buffer: 20 mM HEPES pH 7.5, 0.1 mM TCEP, 10 mM MgCl_2_. Reactions were initiated by the addition of ATP at a final concentration of 1 mM. For single turnover transthiolation assays, E1 (0.5 µM) was pre-loaded with Ub/FAT10 (5 µM) in transthiolation buffer by addition of 1 mM ATP for 10 min. Loading was then quenched by incubation with 50 mM EDTA for 10 min followed by addition of E2 (3 µM). For testing the effect of exogenously added InsP_6_, UBL~loaded UBA6 purified from *E.coli* was incubated with 10-fold molar excess InsP_6_ concurrent with EDTA quenching. Multi-turnover and single-turnover samples were quenched by the addition of LDS sample buffer (Invitrogen) with no reducing agent and analysed by SDS-PAGE gel electrophoresis and visualised by Coomassie. Quantification was performed using ImageJ version 1.54 f and plotted in GraphPad Prism 10.

### UbDha and FAT10^ΔCys^ Dha probe production

UbDha probe was generated as described previously^21^. FAT10^ΔCys^Dha probe was generated by overnight room temperature incubation of 40 µM FAT10^ΔCys,G165C^ with 60-fold molar excess of 2,5-dibromohexanediamide (DBHDA) in 50 mM Na_3_PO_4_, pH 8, 150 mM NaCl. Separation of converted FAT10^ΔCys^Dha from unreacted FAT10^ΔCys,G165C^ was achieved by size exclusion chromatography (Superdex 75, Cytiva) in 50 mM Na_3_PO_4_, pH 8, 150 mM NaCl.

### Purification of UBA6 complexes and grid preparation

UBA6–UbDha–UBE2Z trapped complex was produced by overnight room temperature incubation of 4 μM UBA6^C625S^ with 10 μM UBE2Z and 40 μM UbDha in 20 mM HEPES pH 7.5, 200 mM NaCl, 5 mM MgCl_2_ with 10 mM ATP. The ternary complex was then purified by size exclusion chromatography (Tricorn Superdex 200, Cytiva) in 20 mM HEPES, pH 7.5, 200 mM NaCl, 0.6 mM DTT and concentrated to ~50 µM. UBA6–FAT10^ΔCys^Dha–UBE2Z trapped complex was produced by incubation of 1 µM UBA6^WT^, 3 µM UBE2Z and 210 µM FAT10^ΔCys^Dha in 20 mM HEPES, pH 7.5, 5 mM MgCl_2_, 0.6 mM DTT with 10 mM ATP. Complex formation proceeded overnight at room temperature before purification and a final concentration as for UBA6–UbDha–UBE2Z complex. 0.04% (v/v) fluorinated octyl maltoside was added immediately before application to Cu 300 R1.2/1.3 grids. The sample was then vitrified in liquid ethane by an automated plunge-freezing device (Vitrobot IV, Thermo Fisher Scientific) at 4 °C, 100% humidity and a blot time of 7 s. UBA6 ~ FAT10^ΔCys^ complex sample was generated by incubating 20 µM UBA6^WT^ with 50 µM FAT10^ΔCys^ and 1 mM ATP for 15 min at room temperature, followed by addition of FOM and grid application as for UBA6-Ub/FAT10Dha-UBE2Z complexes.

### Cryo-EM data collection

Grid quality was assessed by preliminary screening using a Talos Arctica microscope (Thermo Fisher Scientific, Central Oxford Structural Molecular Imaging Centre (COSMIC) facility). High-resolution data collections of selected grids followed on a Titan Krios G3 instrument (COSMIC facility) with a Gatan K3 direct electron detector with a bioquantum energy filter. Additional data collection parameters are described in Table 1.

### Cryo-EM data processing

#### UBA6-FAT10^Aden^ and UBA6~FAT10^Thio^

47,574 micrographs were processed using cryoSPARC version 4.7.1^66^ implementation of patch motion correction and patch contrast transfer function (CTF) estimation. Particles were picked using a combination of template-based picking, reference-free blob picking in cryoSPARC, and the deep learning-based particle picker crYOLO^67^. Duplicate particle picks were removed within 40 Å, yielding 17,888,095 unique particles. Extracted particles were subjected to three rounds of two-dimensional (2D) classification, followed by two rounds of ab initio reconstruction and two rounds of heterogeneous refinement^66^, resulting in a particle stack of 2,647,100 particles. Particle CTF parameters were refined (fitting sequentially for tilt, trefoil, anisotropic magnification, and spherical aberration), followed by local CTF refinement and polishing by reference-based motion correction^68^ in cryoSPARC. The polished particle stack was further cleaned by two rounds of ab initio refinement, yielding a final particle stack (1,393,859 particles) from which non-uniform refinement^69^ yielded a 2.8 Å reconstruction of the UBA6-FAT10 complex. Structural heterogeneity was further delineated by three rounds of focused 3D classification using a mask around FAT10^UBL2^ and UBA6^SCCH^. This classification revealed two distinct states of the FAT10 C-terminal tail: 639,990 particles with the FAT10 tail extending toward the ATP binding site (UBA6-FAT10^Aden^) and 753,869 particles with the FAT10 tail extending toward the SCCH domain of UBA6 (UBA6 ~ FAT10^Thio^). Residual heterogeneity within these classes was analysed by multiple rounds of three-dimensional variability analysis (3DVA)^70^ using three principal components. This revealed dynamic motions, including breathing movement of UBA6 domains UFD and SCCH as previously described^21^, flexibility of the FCCH domain moving away from the SCCH domain and the UBA6 core, and shuttling of the FAT10 C-terminal tail between the adenylation and loaded states. Particles were clustered along the principal component dominated by FAT10 tail motion. After multiple rounds of 3DVA, 155,947 particles from the UBA6~FAT10^Thio^ class were reassigned to the adenylation state, redistributing particles into final classes UBA6-FAT10^Aden^ (795,937 particles, 57% of final particle stack) and UBA6 ~ FAT10^Thio^ (223,766 particles, 16% of final particle stack). The remaining 374,156 particles represented transient intermediate states and were not assigned to either class. The two final classes were refined independently using non-uniform refinement, yielding volume reconstructions of UBA6-FAT10^Aden^ at 2.77 Å and of UBA6 ~ FAT10^Thio^ at 3.5 Å.

#### Trapped near-native UBL transthiolation from UBA6 to UBE2Z

##### UBA6-UbDha-UBE2Z dataset

6392 micrographs were pre-processed (motion correction, CTF estimation) on-the-fly using SIMPLE^71^. Particles were picked using template-based picking in SIMPLE, yielding 4,791,444 initial particles. Extracted particles were imported to cryoSPARC version 4.7.1^66^ and were subjected to two rounds of two-dimensional (2D) classification followed by ab initio reconstruction and heterogeneous refinement, resulting in a particle stack of 250,888 particles from which a volume at 3.1 Å could be produced by non-uniform refinement^69^. The cleaned-up particles were then imported into RELION for an additional round of 3D classification and CTF refinements, followed by Bayesian polishing^68^. The polished particles were reimported to cryoSPARC, where non-uniform refinement^69^ now resulted in a consensus volume at a resolution of 2.9 Å. A mask was then manually generated in Chimera X v.1.9 covering the adenylation site of UBA6, which was then used for 3D variability analysis^70^ in cryoSPARC (3 components, mask 0.01 threshold, dilation radius 4 px). In one principal component, the adenylation site occupation varied. Clustering of this component into two clusters allowed for separation of particles into singly-loaded (125,636 particles, 61% of parental particle stack) and doubly-loaded (80,561 particles, 39% of parental particle stack) states, which were then separately refined to consensus maps of 3.0 Å and 3.1 Å, respectively.

### UBA6-FAT10Dha-UBE2Z dataset

14,526 micrographs were pre-processed (motion correction, CTF estimation) in cryoSPARC version 4.7.1 as for the UBA6 ~ FAT10 dataset. Particles were picked using template-based picking in cryoSPARC, yielding 941,556 initial particles. Extracted particles were subjected to two rounds of two-dimensional (2D) classification, followed by ab initio reconstruction and multiple rounds of heterogeneous refinement, yielding a particle stack of 454,329 particles (48% of particles). A further round of heterogeneous refinement to remove additional junk particles gave a particle stack of 312,091 particles from which a volume at 4.8 Å could be produced by non-uniform refinement^69^. These particles were re-extracted using a 384 pixel box size followed by further rounds of heterogeneous and non-uniform refinement. CTF parameters for particles were then refined using global CTF refinement and local CTF refinement, followed by particle polishing performed using reference-based motion correction in cryoSPARC. Non-uniform refinement on the polished particle stack (233,302 particles) gave a 3.04 Å consensus map. The handedness of all maps (consensus and half maps) was then corrected in ChimeraX v.1.9.

### Model building and refinement

FAT10 (PDB: 7PYV])^31^, ubiquitin (PDB: 9B5D)^29^, UBE2Z (PDB: 5A4P)^46^, UBA6 (PDB: 9QIC)^21^ models were initially fitted into the reconstituted EM maps using ChimeraX v.1.10.1^72^. Atomic models were refined by multiple iterative rounds of manual editing in Coot v.0.9.8.96^73^, followed by real-space refinement in PHENIX v.1.21.2^74^. The geometry of all models was validated using Phenix_MolProbity. For the UBA6-FAT10 models, hydrogen atoms were added using phenix.reduce^75^ and covalent-link restraints between the FAT10 Gly167 and UBA6 Cys625 were generated using AceDRG^76^.

### Fluorescent labelling of proteins

Ubiquitin and FAT10 variants were fluorescently labelled with BDP-FL (BDP), sulfo-cyanine-3 (Cy3), or sulfo-cyanine-5 (Cy5) (Lumiprobe) through maleimide labelling of a free cysteine introduced at the N-terminus of each protein as previously described^21,40^.

### Fluorescence polarisation

Serial dilutions of UBA6 and UBE2Z variants were made in 20 mM HEPES pH 7.4, 150 mM NaCl, 4 mM DTT, 0.04 mg/ml BSA and 0.05% (v/v) CHAPS. 30 nM N-terminally Cyanine3 (Cy3) labelled FAT10^ΔCys^ variants or BDP-labelled ubiquitin was added to negative control and sample wells of a 384-well black low binding plate (Corning) followed by addition of the respective UBA6/UBE2Z serial dilution in technical triplicate to give a final FAT10^ΔCys^/ubiquitin concentration of 15 nM. Measurements were performed on a CLARIOstar Plus (BMG LABTECH) using excitation and emission settings of 540 nm and 590 nm (Cy3-FAT10^ΔCys^) or 485 nm and 535 nm (BDP-Ub), with a 20 nm slit-width. All data analysis was performed in GraphPad Prism 10 using non-linear regression (curve fit) assuming single-site binding.

### Reporting summary

Further information on research design is available in the Nature Portfolio Reporting Summary linked to this article.

## Supplementary information

Supplementary Information

Reporting Summary

Transparent Peer Review file

## Source data

Source data

## Data Availability

Cryo-EM maps are deposited in the Electron Microscopy Data Bank (EMDB) under accession numbers: EMD-56614, 56615, 56646, 56645 and 56644. Atomic coordinates were deposited in the PDB under accession numbers: 28MJ, 28MK, 28MQ, 28MP and 28MO. PDB codes of previously published structures used in this study are 9QIA, 5A4P, 7BOL, 1ZDN, 7PYV, 9B5D, 9QHI and 9QIC. Source data are provided as a Source Data file. Source data are provided with this paper.
