# Supplementary material for: Structural determinants for FAT10 activation and transfer from UBA6 to E2 enzymes
Source: Nat Commun. 2026 Aug 13;17:9751. doi: 10.1038/s41467-026-76603-3 (PMC13572539; doi:10.1038/s41467-026-76603-3)
Supplement: Supplementary file 1 [file 41467_2026_76603_MOESM1_ESM.pdf]

Structural determinants for FAT10 activation and transfer from UBA6 to E2 enzymes

Cara J. Ellison[1], Carlos Riechmann[1,2], Evmorfia V. Dalietou[1,2], Michael D. R. Simmons[1], Emma C. Dodd[1] and Paul R. Elliott[1]*

[1]Department of Biochemistry, University of Oxford, South Parks Road, OX1 3QU, UK

[2]Equal contribution

*Correspondence to Paul R. Elliott: paul.elliott@bioch.ox.ac.uk

Supplementary Figures

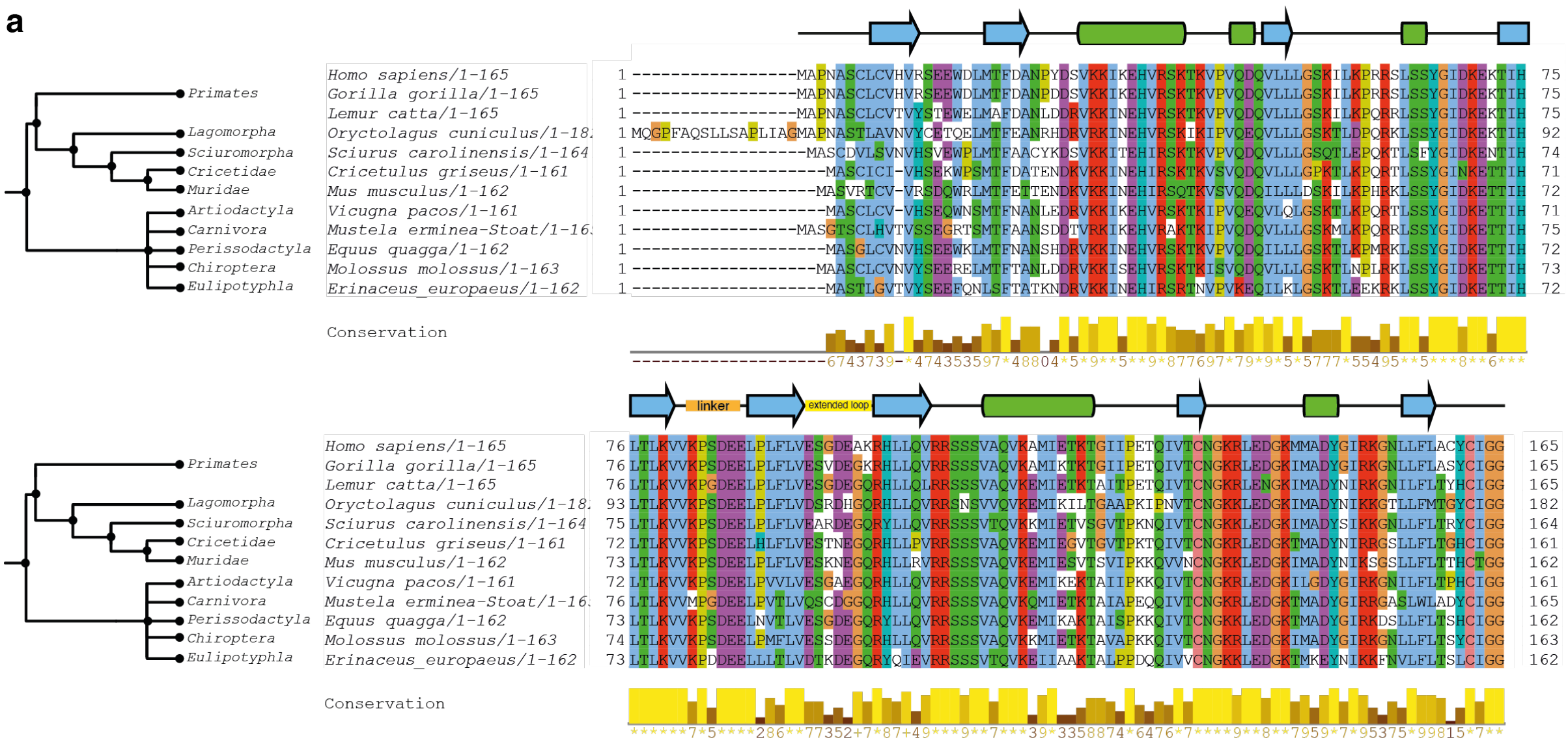

Supplementary Figure 1

a. FAT10 is present in the Boreoeutheria clade of placental mammals. Sequence alignments coloured by residue property (Clustal) highlight high conservation between orthologues. Blue triangle represents β-sheet, green cylinder represents α-helix.

Supplementary Figure 2

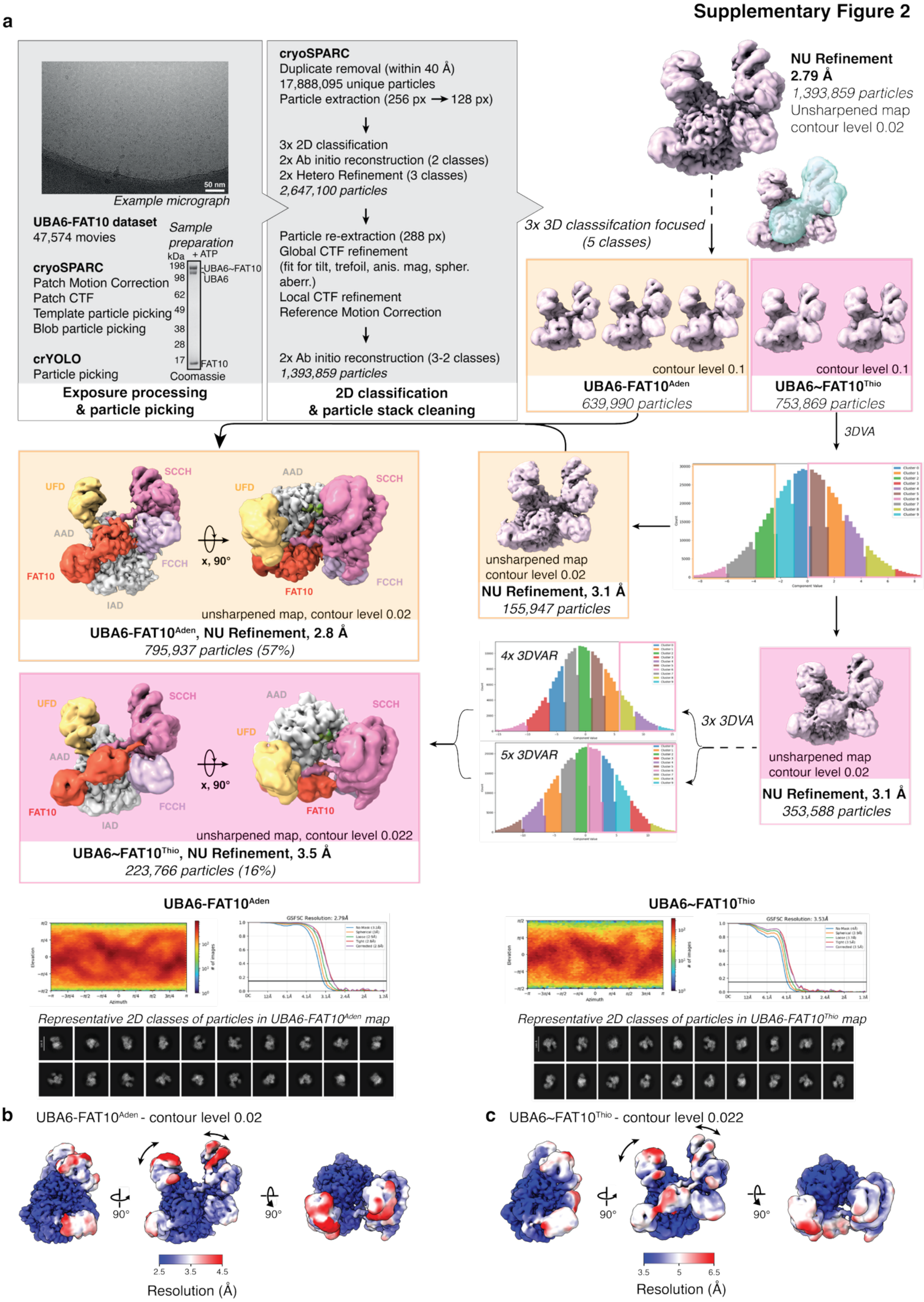

Supplementary Figure 2
Data processing workflow of UBA6 activation (adenylation) and loading (thiolation) of FAT10

a. Processing pipeline performed to generate consensus maps of UBA6-FAT10^{Aden} and UBA6~FAT10^{Thio}.
b. & c. Local resolution of UBA6-FAT10^{Aden} and UBA6~FAT10^{Thio} consensus maps highlights that FAT10^{UBL2} is more rigid in the adenylation state (b) than in the thiolation state (c).

Supplementary Figure 3

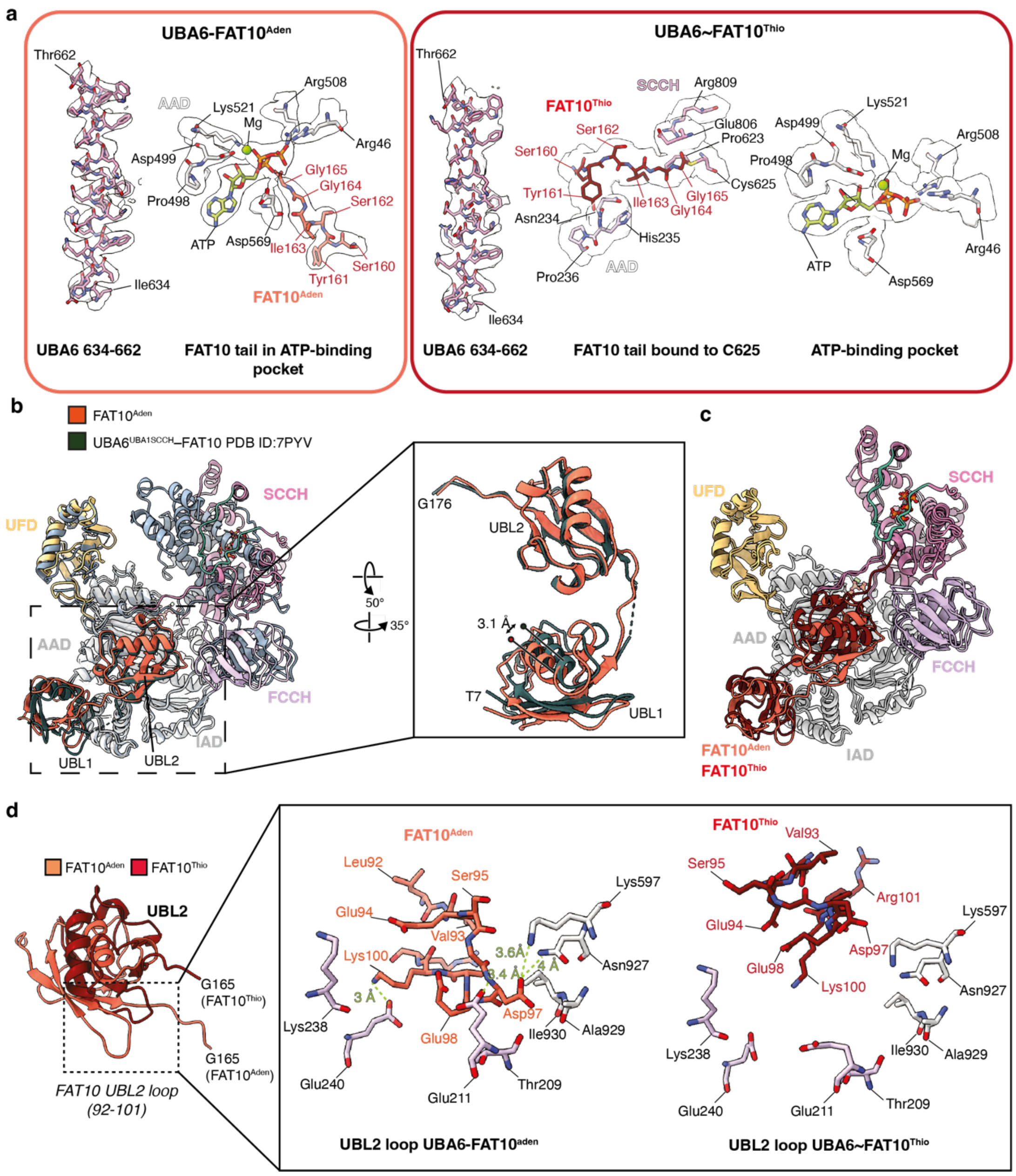

Supplementary Figure 3

a. Cryo-EM density and associated model for selected regions in the UBA6-FAT10^{Aden} and UBA6~FAT10^{Thio} maps: UBA6 SCCH domain (residues 634-662) and FAT10 tail (residues 161-165).
b. Overlaying our UBA6-FAT10^{Aden} model with a model of FAT10 bound to a chimeric UBA6$^{UBA1-SCCH}$ without ATP derived by X-ray crystallography (PDB ID 7PYV)[31] highlighted a minor positional shift in UBL1, whilst UBL2 is positioned identically.
c. Despite the FAT10 C-terminal tail shuttling out of the ATP-pocket to reach the SCCH domain in the post-thiolation state, FAT10^{UBL2} continues to occlude the AAD, highlighted by superimposition of our UBA6-FAT10^{Aden} and UBA6~FAT10^{Thio} models (alignment on UBA6).
d. Rotation of UBL2 between adenylation and post-thiolation breaks interactions between a UBL2 loop (residues 92-101) and the AAD core.

Supplementary Figure 4

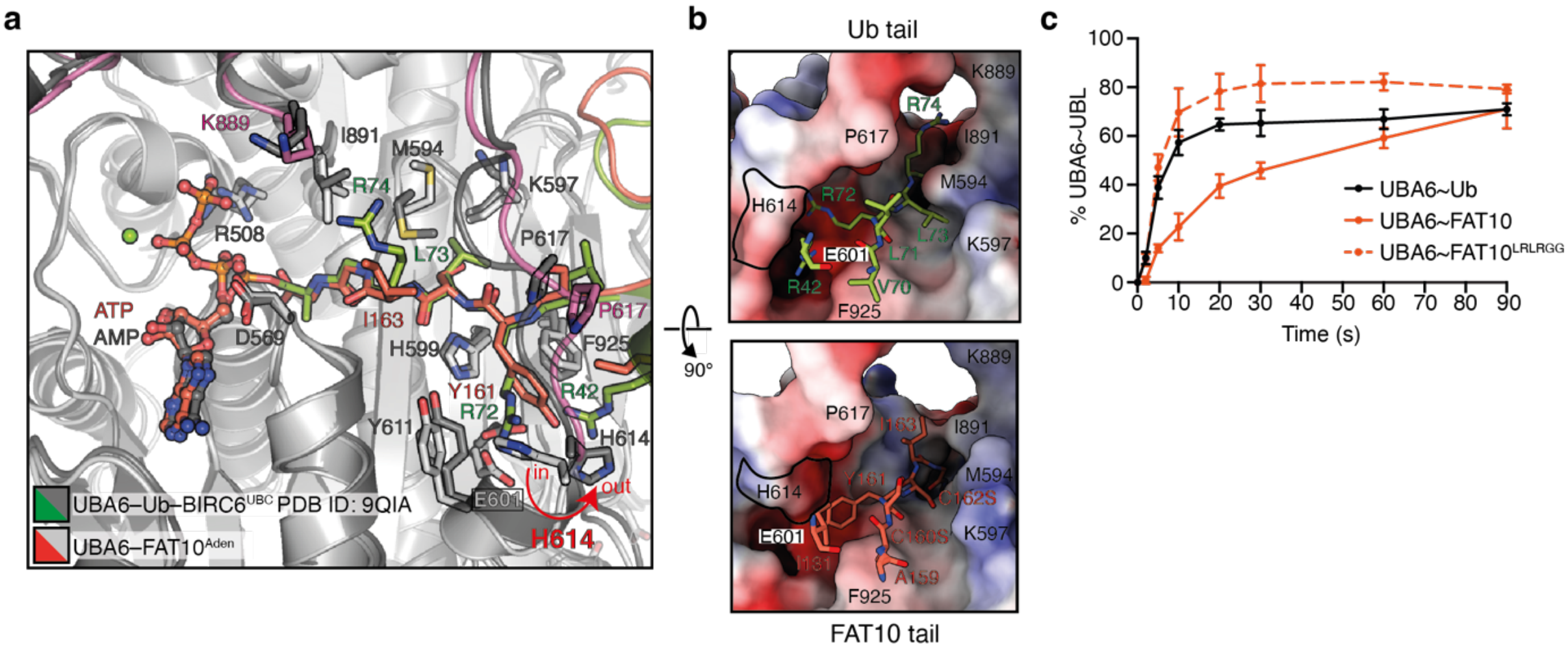

Supplementary Figure 4

The tail of FAT10 is responsible for slower loading onto UBA6

a. Comparing UBA6 residues lining the entry to the ATP-binding pocket in ubiquitin and FAT10 adenylation highlights a differential position of UBA6 His614: in an outward position in the presence of ubiquitin in UBA6–UbDha–BIRC6 (PDB ID 9QIA)[21] but in an inward position in the presence of FAT10 in UBA6-FAT10^{Aden}. The inward position may prevent FAT10 Tyr161 from forming an analogous interaction with UBA6 Glu601 observed with ubiquitin Arg72.
b. Ubiquitin Arg72 reaches into a negatively charged burrow created by His614 movement. In contrast, FAT10 Tyr161 is occluded by the inward position of His614.
c. Exchanging the FAT10 tail with that of ubiquitin mirrors ubiquitin loading as determined in time course in vitro loading assays. Graph represents mean ± S.D. of three independent experiments.

Supplementary Figure 5

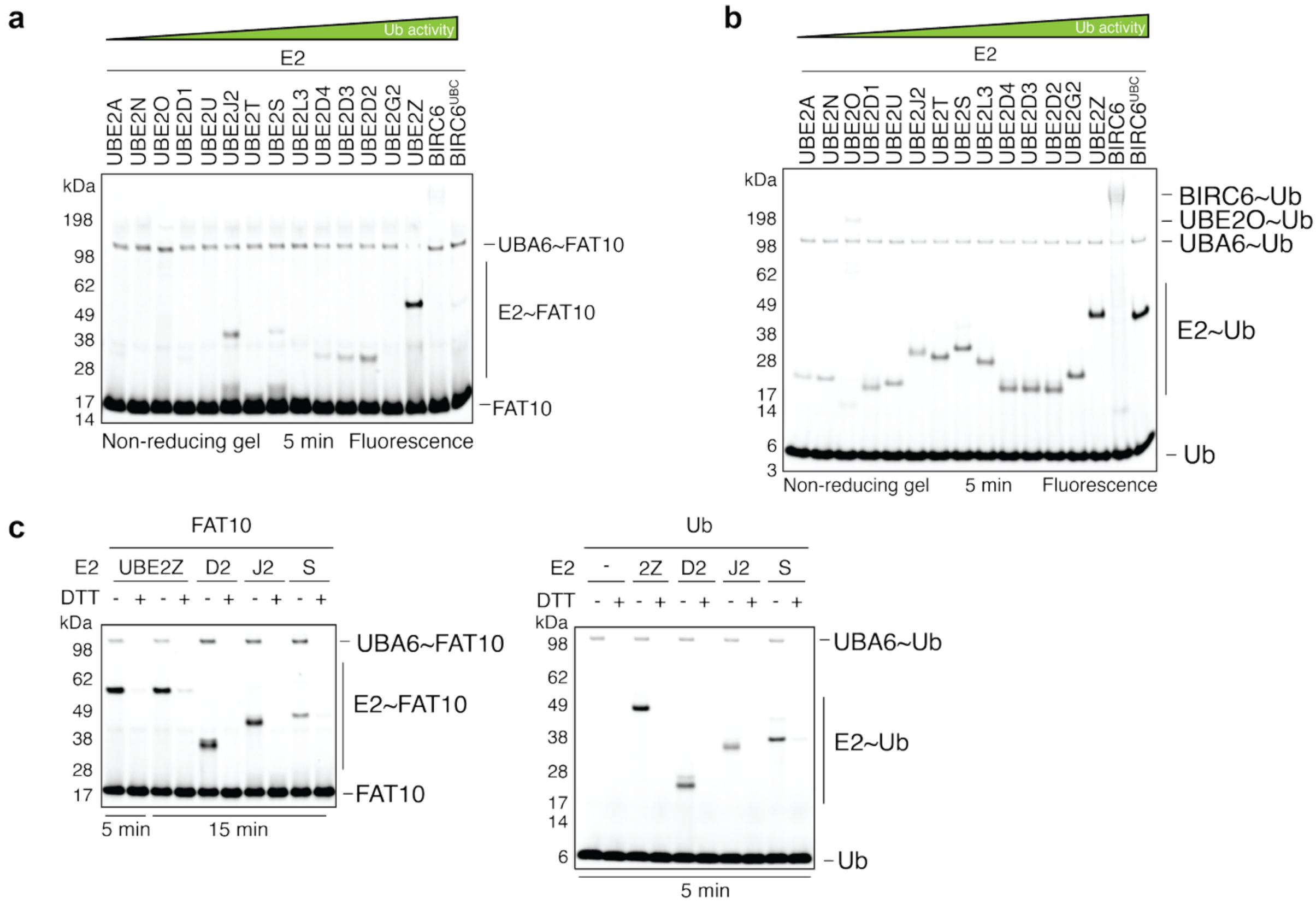

Supplementary Figure 5

A subset of UBA6-compatible E2-ubiquitin conjugating enzymes receive FAT10

a. Of these 15 E2 enzymes, six (UBE2Z, UBE2D2, UBE2D3, UBE2D4, UBE2J2, UBE2S) robustly receive Cy3-FAT10 in multi-turnover in vitro transthiolation assays at 5 minutes. E2 enzymes are ordered by increasing ubiquitin transfer activity highlighting that capability of FAT10 transfer does not directly correlate with ubiquitin transfer activity. Gel is representative of three independent experiments. UBC, ubiquitin-conjugating domain.
b. 15 human E2 ubiquitin-conjugating enzymes receive BDP-ubiquitin from UBA6 shown by multi-turnover in vitro transthiolation assays at 5 minutes. E2 enzymes are ordered by increasing ubiquitin-transfer activity. Gel is representative of three independent experiments.
c. FAT10 and ubiquitin are transferred onto E2 enzymes through a thioester linkage. E2~Cy3FAT10 or E2~BDPUb species formed in multi-turnover in vitro transthiolation reactions were incubated with 200 mM DTT collapsing the E2~UBL thioester bond. Gel is representative of three independent experiments.

Supplementary Figure 6

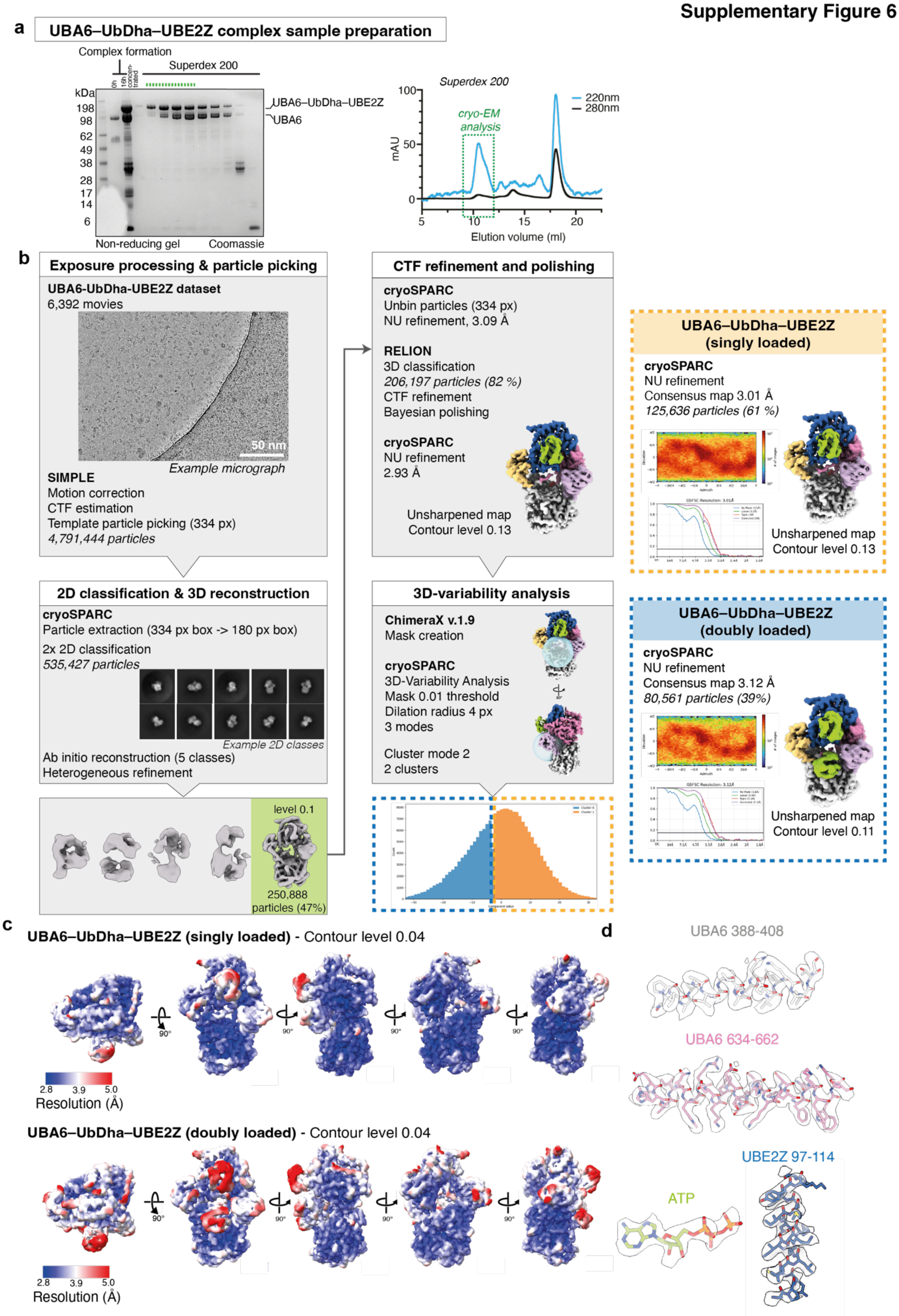

Supplementary Figure 6
Data processing workflow to structurally visualise UBA6 transferring ubiquitin to UBE2Z

a. Outline of UBA6–UbDha–UBE2Z complex preparation.
b. Processing pipeline performed in cryoSPARC to generate consensus maps of UBA6–UbDha–UBE2Z singly- and doubly- ubiquitin loaded.
c. Mapping local resolution onto singly- and doubly-loaded UBA6–UbDha–UBE2Z consensus maps highlights that whilst sufficiently rigid to enable 3D reconstruction, the ubiquitins display a degree of flexibility.
d. Examples of cryo-EM map to model fitting including regions of UBA6 AAD (388-408) and SCCH (634-662) domains, UBE2Z UFD-interacting helix (97-114) and ATP.

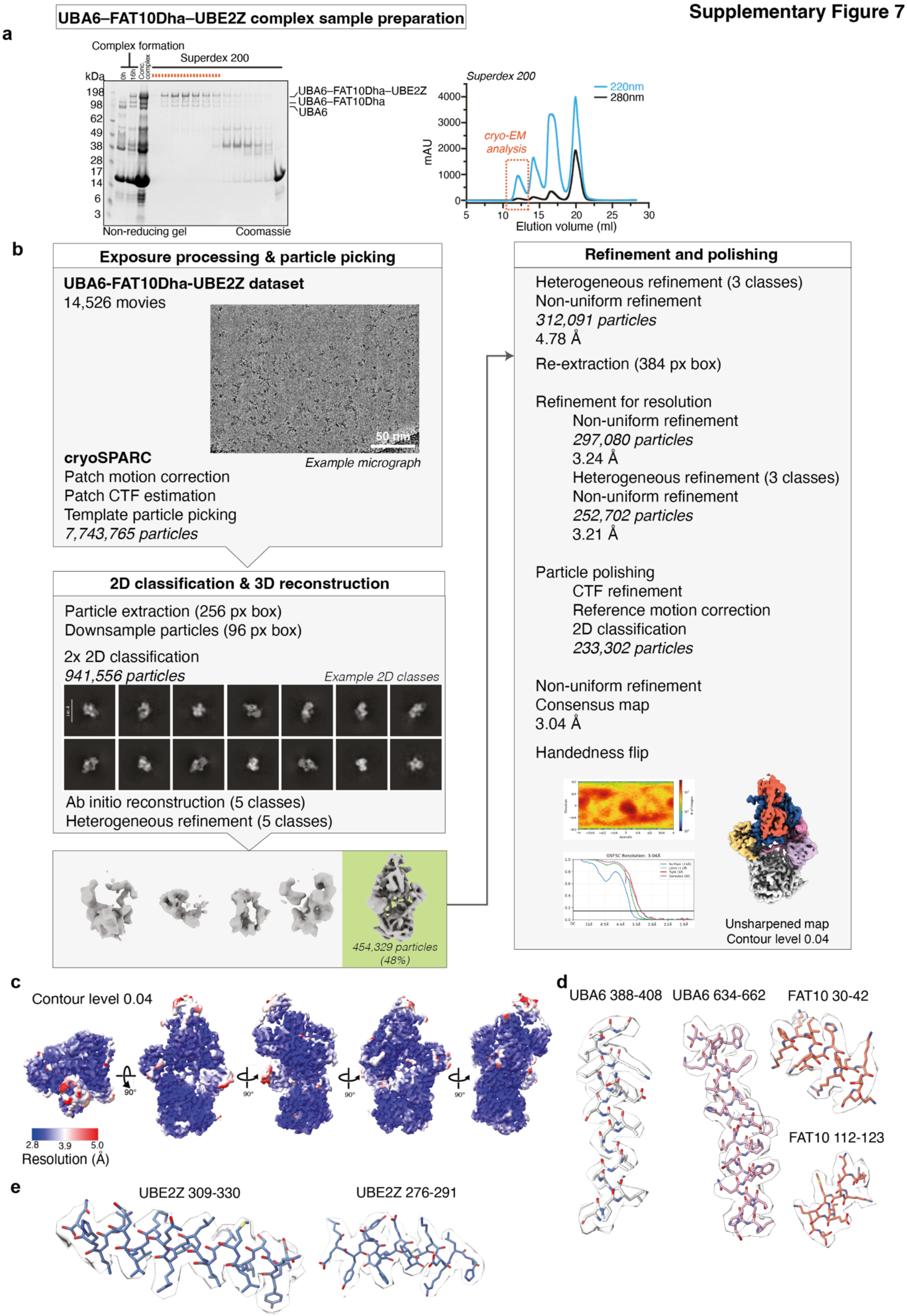

Supplementary Figure 7

Data processing workflow to structurally reveal FAT10 transfer from UBA6 to UBE2Z

a. Outline of UBA6–FAT10Dha–UBE2Z complex preparation.

b. Processing pipeline performed in cryoSPARC to generate a consensus map of UBA6–FAT10Dha–UBE2Z.
c. Mapping local resolution onto UBA6–FAT10Dha–UBE2Z consensus map highlights UBL2 is more rigidly held than UBL1 and more rigid than ubiquitin observed in either UBA6–UbDha–UBE2Z map (**Supplementary Figure 6b and c**).
d. Cryo-EM map to model fitting of select regions of UBA6 AAD (388-408) and SCCH (634-662) domains and $FAT10^{UBL1}$ (30-42) and $FAT10^{UBL2}$ (112-123).
e. Cryo-EM map to model fitting of select regions of UBE2Z-UBL1 interacting helices (309-330 and 276-291).

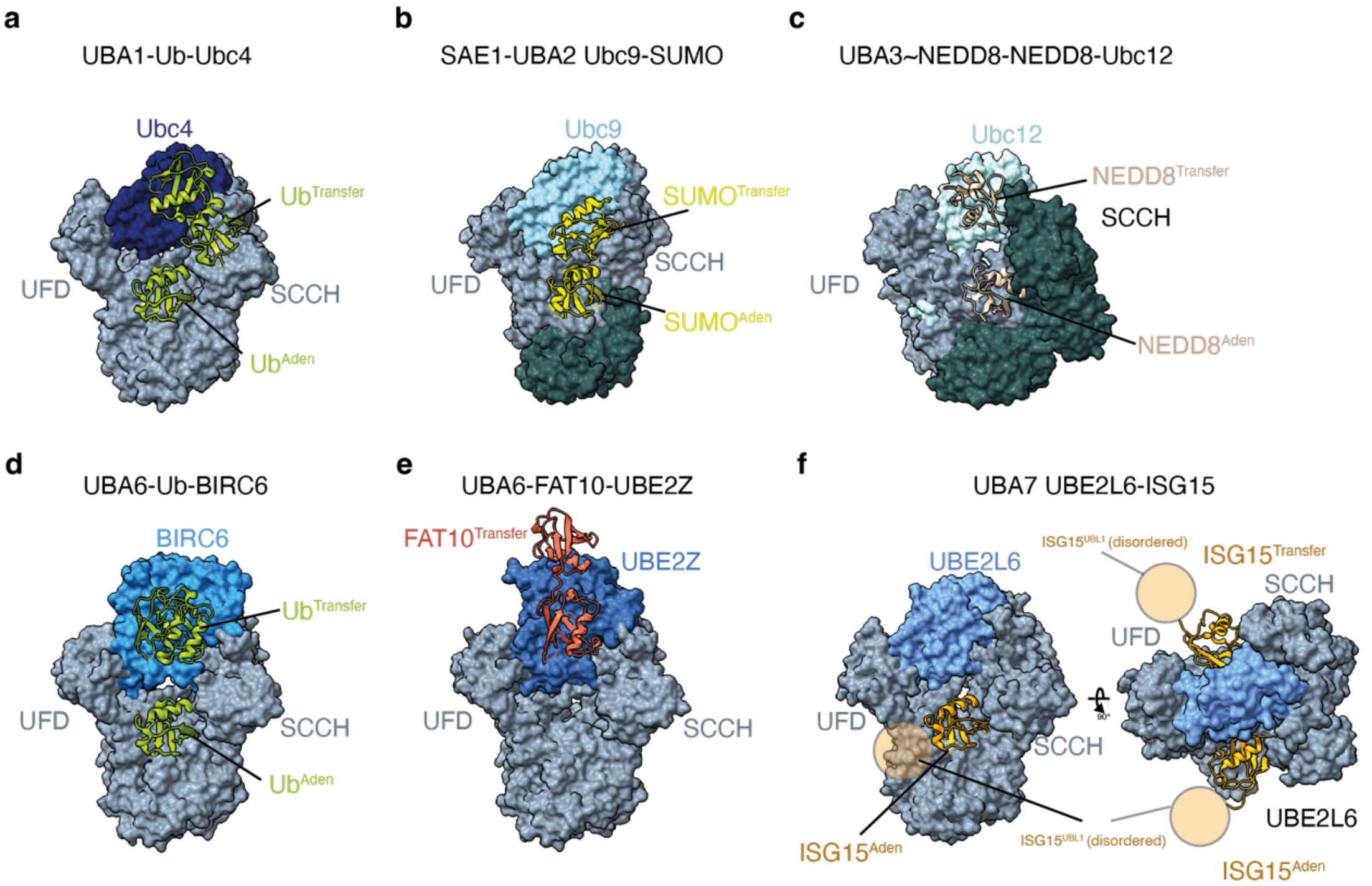

Supplementary Figure 8

E1s and E2s adopt a conserved conformational arrangement for UBL handover

a. – f. Comparison of structures visualising ubiquitin and UBL transfer from E1s to E2s highlights E2s are consistently sandwiched between UFD and SCCH domains of the corresponding E1. Furthermore, with the exception of ISG15, the UBL is transferred onto the analogous ubiquitin-binding patch of the E2.

  a. Composite of ubiquitin transfer from UBA1 to Ubc4 (PDB ID 9B5D, 9B5E)[29] .
  b. SAE1-UBA2 transferring SUMO to Ubc9 (PDB ID 9DQB)[44] .
  c. Nedd8~UBA3 transferring NEDD8 to Ubc12 (PDB ID 2NVU)[32] .
  d. Composite of ubiquitin transfer from UBA6 to BIRC6 (PDB ID 9QHI, 9QIA)[21] .
  e. UBA6 transferring FAT10 to UBE2Z determined in this study.
  f. UBA7 transferring ISG15 to UBE2L6 (PDB ID 8SE9)[45] .

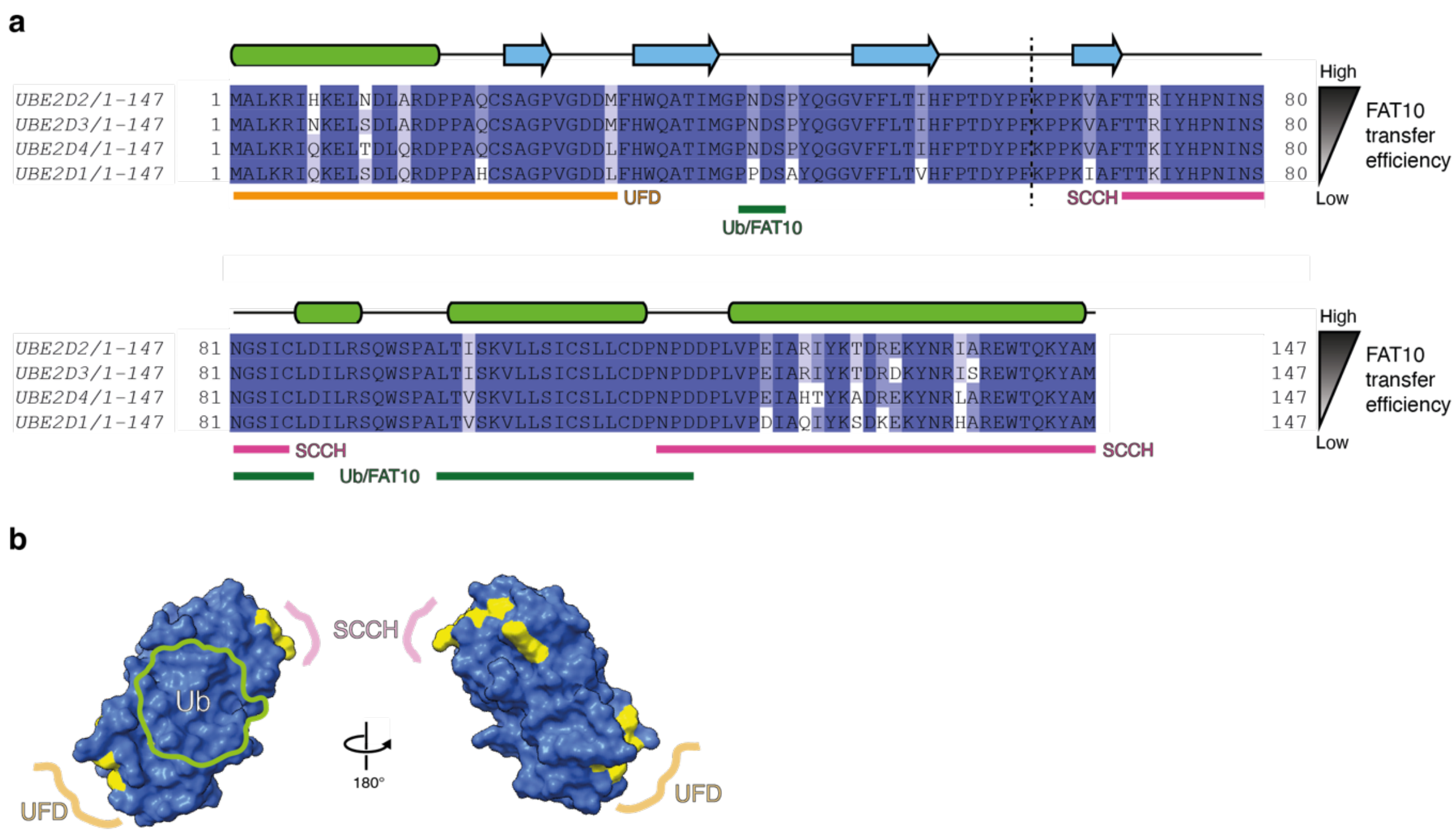

Supplementary Figure 9

The four UBE2D family members display differences in UBA6-interacting regions

a. Sequence alignments of the four UBE2D family members coloured by similarity identifies differences concentrated around the regions interacting with UBA6 UFD and SCCH domains.

b. Structural mapping of the sequence differences (yellow) between UBE2D2 and UBE2D4 highlights an identical interface for ubiquitin^{Transfer}, thus an identical predicted FAT10^{UBL2}-binding site.

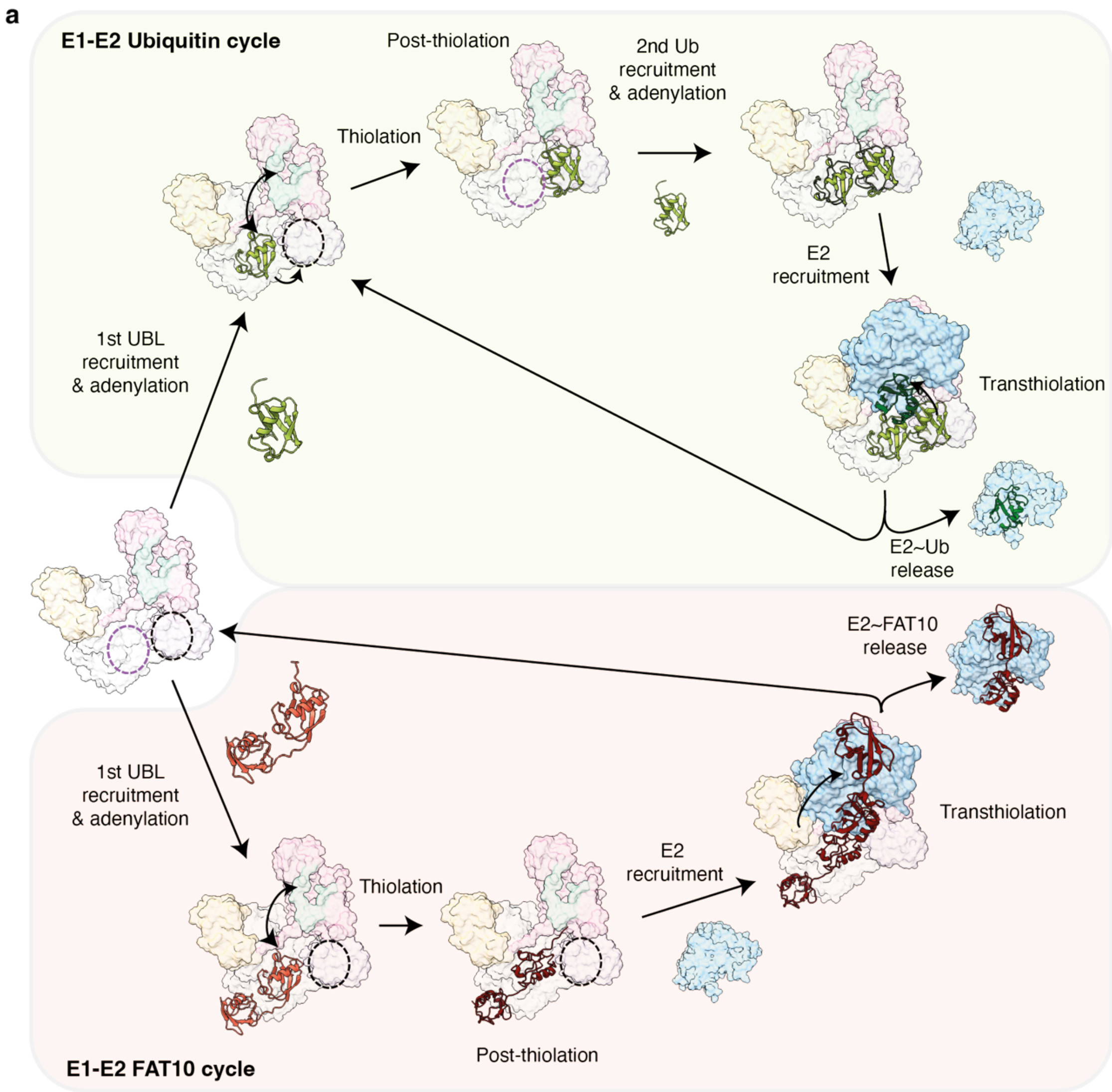

Supplementary Figure 10

UBA6 employs distinct mechanisms for dealing with FAT10 versus ubiquitin

a. Post-thiolation, ubiquitin vacates the adenylation site (purple dashed circle) and occupies a new position on UBA6 supported by the FCCH domain (black dashed circle). A second ubiquitin moiety is then activated and poised for thiolation once the first ubiquitin moiety has been transferred to an E2 and E2~Ub has been released. In contrast, FAT10 continues to occupy the adenylation site post-thiolation preventing another FAT10 from being activated until FAT10 has been transferred to the E2 and E2~FAT10 is released.
