# Supplementary material for: Structural determinants for FAT10 activation and transfer from UBA6 to E2 enzymes
Source: Nat Commun. 2026 Aug 13;17:9751. doi: 10.1038/s41467-026-76603-3 (PMC13572539; doi:10.1038/s41467-026-76603-3)
Supplement: Supplementary file 3 [file 41467_2026_76603_MOESM3_ESM.pdf]

Peer Review File

Structural determinants for FAT10 activation and transfer from UBA6 to E2 enzymes

Corresponding Author: Dr Paul Elliott

This file contains all reviewer reports in order by version, followed by all author rebuttals in order by version.

Version 0:

Reviewer comments:

Reviewer #1

(Remarks to the Author)
In the article “Structural determinants for FAT10 activation and transfer from UBA6 to E2 enzymes” the authors provide interesting new structural insights into how the bi-specific E1 activating enzyme UBA6 and the ubiquitin-like modifier FAT10 interact with each other in order to transfer FAT10 onto specific E2 conjugating enzymes. The authors use cryo-EM to investigate all structural stages of FAT10 activation by UBA6 as well as its transfer to E2 enzymes and confirm UBA6-mediated E2~FAT10 and E2~ubiquitin thioester loading by additional biochemical assays. They provide an interesting explanation for the necessity of the flexible linker between FAT10`s UBL1 and UBL2. As additional interesting and new finding they show that UBA6 acts as a “single passenger” vehicle for FAT10. Their data provide evidence that upon transfer of the FAT10 C-terminus to the UBA6`s active-site cysteine the FAT10 UBL domain stays bound to the adenylation domain, preventing additional loading of a second FAT10 or ubiquitin molecule. They conclude that the full process of FAT10 activation, thiolation and trans-thiolation has to be finished, before a new FAT10 or ubiquitin molecule can be activated by UBA6, thereby outcompeting ubiquitin for being activated by UBA6 in the presence of FAT10. Moreover, they show that the UBL2 domain of FAT10 plays an additional important role during the transfer of FAT10 onto an E2 conjugating enzyme, especially onto UBE2z. In addition, the authors identify several new FAT10-reactive E2 conjugating enzymes and further extend the list of recently identified FAT10-specific E2 enzymes.
Taken together, the authors present a very important piece of data which provides many interesting new information, contributing to a better understanding of the whole mechanism of FAT10 and ubiquitin activation by the bi-specific E1 UBA6 and the transfer onto E2 enzymes.

Specific points
1) In Figure 3a, the authors measure E2~FAT10 loading under non-reducing conditions using FAT10-Cy3. One additional, longer (15 min Fluorescence) exposed gel is shown in Extended Figure 5a with much better signals as compared to the blot shown in Fig. 3a. I would propose to exchange these two blots, since some of the signals for E2~FAT10 are hard to see, even under greater enlargement of the blot.
2) In Figure 6c, the fluorescence signal especially for the UBE2O~FAT10 conjugate is very weak. To strengthen the data, the authors could add a label, such as e.g. an arrow head, to mark the size of UBE2O~FAT10, or - if available - to exchange the blot.
3) UBE2Z/USE1 was shown to undergo a rapid auto-FAT10ylation upon UBA6-mediated transfer of FAT10, however still stays competent for further FAT10 and ubiquitin thioester loading and transfer (Aichem et al., FEBS J. 2014 Apr;281(7):1848-59. doi: 10.1111/febs.12745, and Aichem et al., Nat Commun. 2010 May 4;1:13. doi: 10.1038/ncomms1012.) Since this auto-FAT10ylation was shown under in cellulo as well as under in vitro conditions, it would be interesting to know, if the authors observed this auto-modification in their studies, as well?
4) In line 407 and 434 of the discussion, the authors describe to have identified novel E2 conjugating enzymes for FAT10. However, several of these E2 enzymes, such as UBE2a, UBE2D1-4 , UBE2O, UBE2G2 have only recently been published in a study by Schnell et al. (Life Sci Alliance. 2023 Aug 21;6(11):e202301985. doi: 10.26508/lsa.202301985) as E2 enzymes, capable of being loaded with FAT10 under in cellulo and/or in vitro conditions. The authors mention this publication by the Aichem group already in line 188 of the results section, however they should include the citation of Schnell and colleagues in the discussion, as well.
5) In lines 150- 157 the authors provide an interesting new explanation for the function of the FAT10`s flexible linker. In support of this interesting finding, they might discuss that a deletion of the complete linker, as earlier shown by Aichem et al. (Nature Commun 2018, DOI: 10.1038/s41467-018-05776-3), completely abolished FAT10 activation and loading onto

UBE2Z, as well as FAT10 conjugation.
6) In Fig. 3, the authors investigate the transthiolation reaction of FAT10 onto UBE2z by using the FAT10-DhA probe to trap the ternary intermediate state of UBA6-FAT10-UBE2Z: (line 216ff) "For trapping ubiquitin, we used UBA6 with catalytic Cys625 mutated to Ser to aid stability of the conjugate without impairing UBA6~Ub loading. However, we did not detect robust loading of FAT10Dha onto UBA6C625S, thus UBA6WT was used for trapping FAT10 transfer". Do the authors have an explanation where this difference between ubiquitin and FAT10 comes from? Did they test the efficiency of the Dha reaction after generating the probes, e.g. by Mass spec analysis? Is the generation of the Ub-Dha probe maybe more efficient than that of FAT10-Dha?

Minor points
1) Source data for Figure 3a are missing.
2) For a better understanding of the biochemical E2~FAT10 / ubiquitin gels as shown in Figure 3a, the authors should mention at least once in the Figure legend, that they have used FAT10-Cy3.
3) Is there a specific reason why the authors used Cy3 for labeling of FAT10 while using Cy5 for labeling of ubiquitin, and why they did not use the same labeling for both modifiers?
4) The authors should mention that UBE2z is also alternatively named USE1 (UBA6-specific E2 enzyme 1), since this name is used in many other publications.
5) In Figure 6e: it is difficult to distinguish between the different lines. Maybe it would be better to use different colors for ubiquitin and FAT10, or to separate ubiquitin and FAT10 data into two graphs?

Reviewer #2

(Remarks to the Author)
Ellison et al. describe a series of structures derived by cryo-EM of the bispecific E1 enzyme in complex with ubiquitin or the ubiquitin-like modifier FAT10 at different stages of the catalatic cycle and also in complex with the E2 enzyme UBE2Z. These structural data are accompanied by detailed and insightful biochemical studies. This is an exciting manuscript which significantly enhances our understanding of how UBA6 operates. Among the many novel insights I am especially intrigued by the fact that activation of FAT10 is different than for ubiquitin and other Ubls in that only a single FAT10 is able to bind to UBA6 at any given time. I have no major points of criticism but just a few minor remarks which might further improve this beautiful study.

Specific remarks:
1. Line 30: At that stage the reader does not yet know what "UBL2" is. Please rephrase.
2. Although the terms "thiolation" and "transthiolation" are widely used in the field I would suggest to replace them with "thioesterication" and "transthioesterification". Besides being chemically more accurate it also distinguishes the E1 catalyzed reactions from reactions catalyzed by thioredoxin family member where actual "thiolation" and "transthiolation" reactions occur.
3. Line 53: The authors might want to indicate that "UBL1" is the N-terminal domain and "UBL2" is the C-terminal domain of FAT10.
4.Line 150: Given the accuracy of the structures being compared I would replace "minimal differences" with "essentially unchanged" or similar.
5. Table 1: I always wondered whether the resolution of cryo-EM structures should be given with tow digits after the decimal point, however, this is accepted practice in the field. However, to give the map resolution with three digits after the decimal point as done for 3 out of the 5 structures might be a little too much. Along the same line, it is probably be sufficient to give the B-factors with a precision of one digit after the decimal point. The authors should also indicate which program was used to calculate the sharpening B-factor.
6. Fig. 2: The maps show in panels (a), (d) and (e) are not in line with the reported resolution for these structures. In particular, I cannot reconcile the local resolutions shown in Extended Fig. 2 with the map quality for the UFD, FAT10, FCCH and parts of the SCCH.
7. Extended Figure 5: The authors should define "UBC" in the legend as they did in the main part of the manuscript.
8. Extended Figure 8: It would be easier for the reader if the same color was used for the different E2s.
9. Extended Figure 9: I would conclude that the differences in the UBE2D family are concentrated predominantly in the second SCCH contact area.
10. Line 961: Please indicate which ion exchange chromatography flavor was used for which E2.
11. Finally, please ensure that the details of all figures are legible. At the current resolution this is often not the case, especially when the manuscript is printed. Maybe this will require especially the Extended Figures 2, 6 and 7 to be subdivided.

Reviewer #3

(Remarks to the Author)
This manuscript by Ellison et al. investigates the structural basis by which the E1 enzyme UBA6 functions with both ubiquitin and FAT10. Using a competition assay, the authors show that UBA6 preferentially engages FAT10 over ubiquitin. They then determine structures of UBA6 in complex with FAT10, capturing both the pre-thioester and post-thioester states. These structures suggest that, unlike ubiquitin, FAT10 cannot support double loading onto UBA6. The authors further examine modifier transfer to E2 enzymes, identify UBE2Z as the most efficient cognate E2, and use it for subsequent structural studies. In this context, they present an EM structure of UBA6 in complex with UBE2Z and ubiquitin/FAT10. Finally, they explore the contributions of UBL1 and UBL2 to transfer, as well as the role of InsP6 in mediating interaction with UBE2Z.

The study improves our understanding of how UBA6 functions as a dual E1 for both ubiquitin and FAT10. The structural work is interesting, and the manuscript addresses an important question in the field. However, several issues should be addressed before the manuscript is suitable for publication in Nature Communications.
First, in Fig. 1D, there appears to be detectable binding of UBA6 to ubiquitin, albeit only at higher concentrations. Therefore, it would be more accurate to describe this interaction as weak binding rather than no binding. In addition, although the authors show that UBA6 has higher affinity for FAT10 than for ubiquitin, the basis for this preference remains unclear. The FAT10-UB chimeric constructs generated by the authors(used in fig 5D-F) could be used more effectively to dissect the contribution of each UBL domain to UBA6 binding. Such experiments would help clarify whether the reduced binding observed for ubiquitin is simply due to its single-domain architecture. These fluorescence polarization experiments appear feasible and would substantially strengthen this part of the study.
Second, in Fig. 3A, the authors screen several E2 enzymes for charging by UBA6 and conclude that UBE2Z is the most efficiently charged. However, from the gel it is not fully clear whether the observed band corresponds exclusively to a thioester-linked species rather than, for example, an isopeptide-linked form. Performing the experiment under reducing conditions would help clarify this point.
Third, in Fig. 4H, the authors analyze mutations in different regions of UBE2Z and their effects on activity. As the authors note, it is unexpected that mutations in the UBL2-binding site do not affect ubiquitin binding. This point deserves further exploration. In particular, it would be useful to test additional substitutions at S215 beyond glutamine in order to better define the role of this serine residue in binding. In addition, S208 is not discussed clearly in the text or figure legend and should be addressed.
Fourth, the proposed role of InsP6 in FAT10 transfer to UBE2Z requires stronger experimental support. A more direct test could be performed using recombinant UBA6 produced in E. coli, which should lack endogenous InsP6. The authors could then compare transfer activity in the absence and presence of added InsP6 using wild-type proteins. In parallel, testing the relevant mutant, such as R183, would help determine whether the effect is mediated through UBE2Z binding to InsP6. Such experiments would also help decouple the role of InsP6 in E1 loading versus E2 transfer, especially since the authors suggest these steps may be affected in opposite ways.
Fifth, the authors propose that productive binding of UBE2Z to the UBA6-FAT10 complex is critical for efficient transfer. Based on the presented structures, it would strengthen the mechanistic model if the authors could engineer mutations into other, less efficient E2 enzymes to make them more UBE2Z-like and test whether these substitutions improve FAT10 transfer. This would provide stronger functional validation of the proposed structural mechanism.

Finally The conclusion that the FAT10 tail is responsible for E2 selectivity (line 333) is not entirely clear to the reviewer. Figure 5d shows that even when the C-terminal tail residues of FAT10 are retained, the fused UBL loses E2 selectivity. In contrast, Figure 5f shows that the fused UBL retains E2 selectivity even in the absence of the FAT10 C-terminal tail residues. Together, these results suggest that E2 selectivity is determined primarily by the UBL2 domain rather than by the C-terminal tail residues.

Minor points:
1. In the text, the authors refer to Extended Fig. 2e, but this panel is not presented.
2. In line 270, it should be Asp97 rather than Glu97, since in Fig. 4e it is labeled as D97.
3. Extended Fig. 10 is not clear, particularly the transthiolation of FAT10 onto UBE2Z.

Reviewer #4

(Remarks to the Author)
I co-reviewed this manuscript with one of the reviewers who provided the listed reports. This is part of the Nature Communications initiative to facilitate training in peer review and to provide appropriate recognition for Early Career Researchers who co-review manuscripts.

Version 1:

Reviewer comments:

Reviewer #3

(Remarks to the Author)
The authors have addressed all of my concerns, and I support publication in Nature Communications.

Reviewer #4

(Remarks to the Author)
I co-reviewed this manuscript with one of the reviewers who provided the listed reports. This is part of the Nature Communications initiative to facilitate training in peer review and to provide appropriate recognition for Early Career Researchers who co-review manuscripts.

Open Access This Peer Review File is licensed under a Creative Commons Attribution 4.0 International License, which permits use, sharing, adaptation, distribution and reproduction in any medium or format, as long as you give appropriate credit to the original author(s) and the source, provide a link to the Creative Commons license, and indicate if changes were made.
In cases where reviewers are anonymous, credit should be given to 'Anonymous Referee' and the source.
The images or other third party material in this Peer Review File are included in the article's Creative Commons license, unless indicated otherwise in a credit line to the material. If material is not included in the article's Creative Commons license and your intended use is not permitted by statutory regulation or exceeds the permitted use, you will need to obtain permission directly from the copyright holder.
To view a copy of this license, visit https://creativecommons.org/licenses/by/4.0/

REVIEWER COMMENTS

We thank the reviewers for their time and constructive critique of our manuscript. We are delighted that they share our enthusiasm for our mechanistic insights into FAT10 activation and transfer onto specific E2s. We found all comments very insightful and have incorporated all suggestions to strengthen our manuscript. Upon the suggestion of reviewer #3, we have expanded our insights into the role of inositol hexaphosphate ($InsP_6$) in transfer of FAT10 onto different E2s. Using UBA6 purified from *E. coli* which lacks $InsP_6$, we observed exogenous addition of $InsP_6$ enhances FAT10 transfer to UBE2Z, UBE2D2 and UBE2J2 supporting our original data mutating residues within the E2 that co-ordinate $InsP_6$. Interestingly, whilst most FAT10-competent E2s displayed a positive requirement for $InsP_6$, we observed $InsP_6$ limits FAT10 transfer onto UBE2S. Upon modelling UBE2S interacting with UBA6 SCCH domain we observed the presence of two negatively charged residues that would form repulsive interactions with $InsP_6$. Mutation of these residues to remove the negative charges (E131A and E132A) dramatically improved FAT10 transfer, Our data showing $InsP_6$ modulates FAT10 transfer points towards a model in which variation of $InsP_6$ levels allows FAT10 conjugation to be fine-tuned in different cellular scenarios.

Please see our point-by-point response to each reviewer's comments below.

Reviewer #1 (Remarks to the Author):

In the article "Structural determinants for FAT10 activation and transfer from UBA6 to E2 enzymes" the authors provide interesting new structural insights into how the bi-specific E1 activating enzyme UBA6 and the ubiquitin-like modifier FAT10 interact with each other in order to transfer FAT10 onto specific E2 conjugating enzymes. The authors use cryo-EM to investigate all structural stages of FAT10 activation by UBA6 as well as its transfer to E2 enzymes and confirm UBA6-mediated E2~FAT10 and E2~ubiquitin thioester loading by additional biochemical assays. They provide an interesting explanation for the necessity of the flexible linker between FAT10`s UBL1 and UBL2. As additional interesting and new finding they show that UBA6 acts as a "single passenger" vehicle for FAT10. Their data provide evidence that upon transfer of the FAT10 C-terminus to the UBA6`s active-site cysteine the FAT10 UBL domain stays bound to the adenylation domain, preventing additional loading of a second FAT10 or ubiquitin molecule. They conclude that the full process of FAT10 activation, thiolation and trans-thiolation has to be finished, before a new FAT10 or ubiquitin molecule can be activated by UBA6, thereby outcompeting ubiquitin for being activated by UBA6 in the presence of FAT10. Moreover, they show that the UBL2 domain of FAT10 plays an additional important role during the transfer of FAT10 onto an E2 conjugating enzyme, especially onto UBE2z. In addition, the authors identify several new FAT10-reactive E2 conjugating enzymes and further extend the list of recently identified FAT10-specific E2 enzymes.
Taken together, the authors present a very important piece of data which provides many interesting new information, contributing to a better understanding of the whole mechanism of FAT10 and ubiquitin activation by the bi-specific E1 UBA6 and the transfer onto E2 enzymes.

We thank the reviewer for their positive assessment of our work.

Specific points
1) In Figure 3a, the authors measure E2~FAT10 loading under non-reducing conditions using FAT10-Cy3. One additional, longer (15 min Fluorescence) exposed gel is shown in Extended Figure 5a with much better signals as compared to the blot shown in Fig. 3a. I would propose to exchange these two blots, since some of the signals for E2~FAT10 are hard to see, even under greater enlargement of the blot.

We agree with the reviewer that swapping the two gels around makes it clearer for the reader. We have now done this.

2) In Figure 6c, the fluorescence signal especially for the UBE2O~FAT10 conjugate is very weak. To strengthen the data, the authors could add a label, such as e.g. an arrow head, to mark the size of UBE2O~FAT10, or - if available - to exchange the blot.

We have now annotated the gel to highlight E2~FAT10 conjugates.

3) UBE2Z/USE1 was shown to undergo a rapid auto-FAT10ylation upon UBA6-mediated transfer of FAT10, however still stays competent for further FAT10 and ubiquitin thioester loading and transfer (Aichem et al., FEBS J. 2014 Apr;281(7):1848-59. doi: 10.1111/febs.12745, and Aichem et al., Nat Commun. 2010 May 4;1:13. doi: 10.1038/ncomms1012.) Since this auto-FAT10ylation was shown under in cellulo as well as under in vitro conditions, it would be interesting to know, if the authors observed this auto-modification in their studies, as well?

We agree that there is evidence within the literature to show that UBE2Z can undergo FAT10ylation. In response to this point, and a suggestion made by reviewer 3, we treated samples after transthiolation with DTT that readily reduces the E2~FAT10 thioester linkage while preserving oxyester or isopeptide linkages. We observe a small level of FAT10 remaining on UBE2Z (new **Extended Figure 5c**) consistent with the literature. We have now mentioned this in the text.

4) In line 407 and 434 of the discussion, the authors describe to have identified novel E2 conjugating enzymes for FAT10. However, several of these E2 enzymes, such as UBE2a, UBE2D1-4 , UBE2O, UBE2G2 have only recently been published in a study by Schnell et al. (Life Sci Alliance. 2023 Aug 21;6(11):e202301985. doi: 10.26508/lsa.202301985) as E2 enzymes, capable of being loaded with FAT10 under in cellulo and/or in vitro conditions. The authors mention this publication by the Aichem group already in line 188 of the results section, however they should include the citation of Schnell and colleagues in the discussion, as well.

We have now incorporated this reference into our Discussion.

5) In lines 150- 157 the authors provide an interesting new explanation for the function of the FAT10`s flexible linker. In support of this interesting finding, they might discuss that a deletion of the complete linker, as earlier shown by Aichem et al. (Nature Commun 2018, DOI: 10.1038/s41467-018-05776-3), completely abolished FAT10 activation and loading onto UBE2Z, as well as FAT10 conjugation.

Thank you, we now have refined the description of this paper and how our study mechanistically explains the requirement of the linker.

6) In Fig. 3, the authors investigate the transthiolation reaction of FAT10 onto UBE2z by using the FAT10-DhA probe to trap the ternary intermediate state of UBA6-FAT10-UBE2Z: (line 216ff) “For trapping ubiquitin, we used UBA6 with catalytic Cys625 mutated to Ser to aid stability of the conjugate without impairing UBA6~Ub loading. However, we did not detect robust loading of FAT10Dha onto UBA6C625S, thus UBA6WT was used for trapping FAT10 transfer”. Do the authors have an explanation where this difference between ubiquitin and FAT10 comes from? Did they test the efficiency of the Dha reaction after generating the

probes, e.g. by Mass spec analysis? Is the generation of the Ub-Dha probe maybe more efficient than that of FAT10-Dha?

We thank the reviewer for their astute observation. Yes, we used intact mass spectrometry to confirm conversion of both Ub G76C and FAT10 G165C into their respective dehydroalanine species. Ub G76C was fully converted to Ub-Dha. While no unconverted FAT10 G165C remained, there was a significant portion of intermediate formed (**Rebuttal Figure 1**), which we think likely reflects its slow reactivity with UBA6.

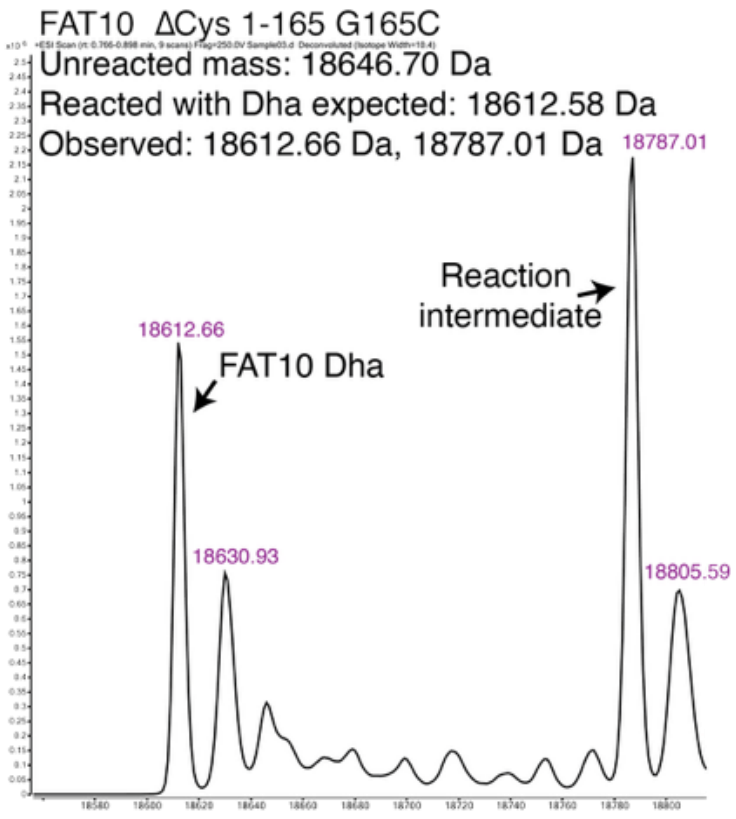

Rebuttal Figure 1.

Minor points

1) Source data for Figure 3a are missing.

We apologise for this omission, source data is now updated.

2) For a better understanding of the biochemical E2~FAT10 / ubiquitin gels as shown in Figure 3a, the authors should mention at least once in the Figure legend, that they have used FAT10-Cy3.

We have now included this in the legend of Figure 3a for clarity.

3) Is there a specific reason why the authors used Cy3 for labeling of FAT10 while using Cy5 for labeling of ubiquitin, and why they did not use the same labeling for both modifiers?

We thank the reviewer for asking this. The reason we used different fluorophores was to enable clear distinction between UBA6~Ub and UBA6~FAT10 in a competition assay. Cy3 and Cy5 emission detected on our imaging device (iBright, Thermofisher) are non-overlapping signals.

4) The authors should mention that UBE2z is also alternatively named USE1 (UBA6-specific E2 enzyme 1), since this name is used in many other publications.

We thank the reviewer for making this important point, we now include this early on in the manuscript.

5) In Figure 6e: it is difficult to distinguish between the different lines. Maybe it would be better to use different colors for ubiquitin and FAT10, or to separate ubiquitin and FAT10 data into two graphs?

We agree that the graph is somewhat busy. We have now split this into two (**Figure 6e, f**).

Reviewer #2 (Remarks to the Author):

Ellison et al. describe a series of structures derived by cryo-EM of the bispecific E1 enzyme in complex with ubiquitin or the ubiquitin-like modifier FAT10 at different stages of the catalatic cycle and also in complex with the E2 enzyme UBE2Z. These structural data are accompanied by detailed and insightful biochemical studies. This is an exciting manuscript which significantly enhances our understanding of how UBA6 operates. Among the many novel insights I am especially intrigued by the fact that activation of FAT10 is different than for ubiquitin and other Ubls in that only a single FAT10 is able to bind to UBA6 at any given time. I have no major points of criticism but just a few minor remarks which might further improve this beautiful study.

We thank the reviewer for their positive evaluation of our work.

Specific remarks:
1. Line 30: At that stage the reader does not yet know what "UBL2" is. Please rephrase.

Thank you for this suggestion. We have now adjusted the text accordingly.

2. Although the terms "thiolation" and "transthiolation" are widely used in the field I would suggest to replace them with "thioesterication" and "transthioesterification". Besides being chemically more accurate it also distinguishes the E1 catalyzed reactions from reactions catalyzed by thioredoxin family member where actual "thiolation" and "transthiolation" reactions occur.

We thank the reviewer for raising this important point. We agree that we have followed the description widely used in the literature to describe these reactions and we agree that transthioesterification is the more correct chemical description of what occurs between handover from E1 to E2. To avoid confusion, we now include both terminologies at the beginning of the manuscript.

3. Line 53: The authors might want to indicate that "UBL1" is the N-terminal domain and "UBL2" is the C-terminal domain of FAT10.

Following the reviewer's suggestion we have made this clarification.

4.Line 150: Given the accuracy of the structures being compared I would replace "minimal differences" with "essentially unchanged" or similar.

We have now adjusted this phrasing.

5. Table 1: I always wondered whether the resolution of cryo-EM structures should be given with tow digits after the decimal point, however, this is accepted practice in the field. However, to give the map resolution with three digits after the decimal point as done for 3 out of the 5 structures might be a little too much. Along the same line, it is probably be sufficient to give the B-factors with a precision of one digit after the decimal point. The authors should also indicate which program was used to calculate the sharpening B-factor.

We agree with the reviewer that the precision of our structures does preclude us from reporting resolutions and B-factors to such a number of significant figures. We have adjusted the data table

accordingly. We used PHENIX v1.21.2 for refinement of all models and we have now added this into **Table 1** to save the reader needing to refer back to the Materials & Methods.

6. Fig. 2: The maps show in panels (a), (d) and (e) are not in line with the reported resolution for these structures. In particular, I cannot reconcile the local resolutions shown in Extended Fig. 2 with the map quality for the UFD, FAT10, FCCH and parts of the SCCH.

We thank the reviewer for pointing this out. The local resolutions shown in **Extended Figure 2b** range from 2.5 Å – 4.5 Å, whereas those in **Extended Figure 2c** range from 3.5 Å -6.5 Å, which from the colour key understandably gives the impression that they are of the same local resolution. The local resolution in **Figures 2a,b** and **2d,e** is still reflective of these differences, but we agree that without careful studying of the figure, it could be confusing. We now report the map resolution in **Figure 2** so that the reader can appreciate the lower local resolution for the UBA6~FAT10 thioester linked structure.

7. Extended Figure 5: The authors should define "UBC" in the legend as they did in the main part of the manuscript.

Thank you, we have now defined it.

8. Extended Figure 8: It would be easier for the reader if the same color was used for the different E2s.

We opted to use different shades of blue for each E2 to avoid giving the incorrect impression that these are the same E2 in complex with different E1s.

9. Extended Figure 9: I would conclude that the differences in the UBE2D family are concentrated predominantly in the second SCCH contact area.

We agree that there are more variations within the SCCH interacting region compared to UFD-interacting region across the UBE2D family. However, in our UBE2D family chimeras we exchange the UFD domain interacting regions between D2 and D4 whilst retaining the differences in SCCH interacting residues. This UFD-only swap dramatically affects FAT10 transfer tracking with UBE2D2's UFD-interacting residues being the driver. We have now included a line indicating our chimera substitutions in **Extended Figure 9a** to make this clearer.

10. Line 961: Please indicate which ion exchange chromatography flavor was used for which E2.

We thank the reviewer for suggesting this. We have now extended our description of ion exchange chromatography used for the UBA6-competent E2s in addition to referencing our work detailing purification of all UBA1/UBA6 Ub-conjugating E2 enzymes (ref 21 in the main text, PMID 41350950).

11. Finally, please ensure that the details of all figures are legible. At the current resolution this is often not the case, especially when the manuscript is printed. Maybe this will require especially the Extended Figures 2, 6 and 7 to be subdivided.

We apologise for the small font size used in some of the extended figures. We are limited by the number of Extended Figures and worry that moving some to supplemental may be more inconvenient for readers to refer to. However, we have taken on board the reviewer's comment and now make sure the details of these figures are more legible to the reader.

Reviewer #3 (Remarks to the Author):

This manuscript by Ellison et al. investigates the structural basis by which the E1 enzyme UBA6 functions with both ubiquitin and FAT10. Using a competition assay, the authors show that UBA6 preferentially engages FAT10 over ubiquitin. They then determine structures of UBA6 in complex with FAT10, capturing both the pre-thioester and post-thioester states. These structures suggest that, unlike ubiquitin, FAT10 cannot support double loading onto UBA6. The authors further examine modifier transfer to E2 enzymes, identify UBE2Z as the most efficient cognate E2, and use it for subsequent structural studies. In this context, they present an EM structure of UBA6 in complex with UBE2Z and ubiquitin/FAT10. Finally, they explore the contributions of UBL1 and UBL2 to transfer, as well as the role of InsP6 in mediating interaction with UBE2Z.
The study improves our understanding of how UBA6 functions as a dual E1 for both ubiquitin and FAT10. The structural work is interesting, and the manuscript addresses an important question in the field. However, several issues should be addressed before the manuscript is suitable for publication in Nature Communications.

We thank the reviewer for taking the time to review our manuscript and for offering helpful suggestions to further strengthen our study.

First, in Fig. 1D, there appears to be detectable binding of UBA6 to ubiquitin, albeit only at higher concentrations. Therefore, it would be more accurate to describe this interaction as weak binding rather than no binding.

We agree with the reviewer and have altered the text and figure accordingly.

In addition, although the authors show that UBA6 has higher affinity for FAT10 than for ubiquitin, the basis for this preference remains unclear. The FAT10-UB chimeric constructs generated by the authors(used in fig 5D-F) could be used more effectively to dissect the contribution of each UBL domain to UBA6 binding. Such experiments would help clarify whether the reduced binding observed for ubiquitin is simply due to its single-domain architecture. These fluorescence polarization experiments appear feasible and would substantially strengthen this part of the study.

We thank the reviewer for this helpful suggestion. Following this we purified, and Cy3-labelled, individual N-terminal and C-terminal UBL domains of FAT10 and measured binding to UBA6 using the same fluorescence polarisation assay as described in **Figure 1d**. Here we observed stronger binding for UBL1 over UBL2 (**Figure 2g**) suggesting UBL1 is the main driver of FAT10 recruitment. This is in line with work from the Schindelin Lab who demonstrated that UBL1-UBA6 interaction is required for FAT10 loading (PMID 35970836). To address whether the observed weaker Ub affinity is due to a single domain architecture, we tested our UBL1-Ub hybrid and observed increased binding supporting UBL1's anchoring capability (**Rebuttal Figure 2**). Interestingly, although Ub binds tighter than UBL2, the measured affinity of UBL1-Ub is weaker than full-length FAT10 (1.9 μM versus 660 nM) likely reflecting suboptimal positioning of Ub when artificially anchored.

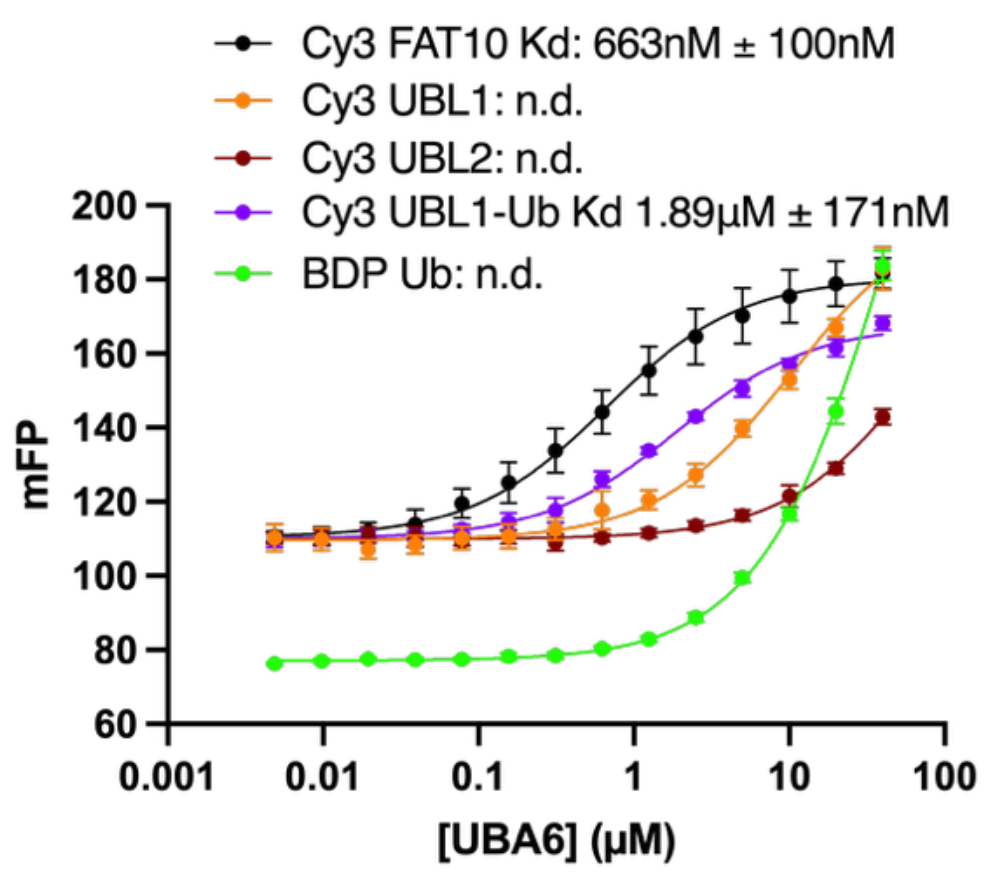

***Rebuttal Figure 2**: Fluorescence polarisation measuring binding of UBA6 to Cy3-labelled FAT10 variants (Full-length, UBL1, UBL2 or UBL1-Ub hybrid) or BDP-labelled ubiquitin. Graph represents mean ± S.D. of three independent experiments each performed in technical replicate.*

Second, in Fig. 3A, the authors screen several E2 enzymes for charging by UBA6 and conclude that UBE2Z is the most efficiently charged. However, from the gel it is not fully clear whether the observed band corresponds exclusively to a thioester-linked species rather than, for example, an isopeptide-linked form. Performing the experiment under reducing conditions would help clarify this point.

We thank the reviewer for suggesting this assay. This also relates to reviewer 1, who asked whether UBE2Z can undergo auto-FAT10ylation in vitro as demonstrated in cellulo in the literature. We have performed this experiment with both Ub and FAT10 and observed that most species collapse in the presence of DTT, indicative of thioester-linked species (**Extended Figure 5c**). As mentioned in response to reviewer 1, a minor level of UBE2Z conjugated to FAT10 remained in the presence of DTT indicative of minor discharge onto a lysine (or possibly threonine/serine) in vitro in 15 minutes.

Third, in Fig. 4H, the authors analyze mutations in different regions of UBE2Z and their effects on activity. As the authors note, it is unexpected that mutations in the UBL2-binding site do not affect ubiquitin binding. This point deserves further exploration. In particular, it would be useful to test additional substitutions at S215 beyond glutamine in order to better define the role of this serine residue in binding. In addition, S208 is not discussed clearly in the text or figure legend and should be addressed.

We thank the reviewer for this suggestion. Mutating Ser215 to Gln decreases FAT10 loading onto UBE2Z without affecting ubiquitin, thus contributing to our toolbox of mutations that will be useful for future functional studies where we can switch off FAT10 activity yet retain ubiquitin activity. We feel that mutating Ser215 to other residues would bring little additional insights to this observation. However, following the reviewer's suggestion, we have now incorporated a revised figure (**Figure 4d**) visualising the interactions from UBE2Z Ser215 and Ser208 to Ub and FAT10 UBL2 and we have included a discussion of our S208Q mutation.

Fourth, the proposed role of InsP6 in FAT10 transfer to UBE2Z requires stronger experimental support. A more direct test could be performed using recombinant UBA6 produced in E. coli, which should lack endogenous InsP6. The authors could then compare transfer activity in the absence and presence of added InsP6 using wild-type proteins. In parallel, testing the relevant mutant, such as R183, would help determine whether the effect is mediated through UBE2Z binding to InsP6. Such experiments would also help decouple the role of InsP6 in E1 loading versus E2 transfer, especially since the authors suggest these steps may be affected in opposite ways.

We thank the reviewer for this suggestion. We have now performed these additional experiments in which we prepared UBA6 from *E. coli* that lacks endogenous $InsP_6$ and tested transfer to the best FAT10-functioning E2s in the presence of exogenously added $InsP_6$. Although FAT10 loads faster onto UBA6 in the absence of $InsP_6$ (**Figure 6f**, and PMID 35986001), we observe $InsP_6$ aids transfer of FAT10 from UBA6 to UBE2Z, UBE2J2 and UBE2D2 (**Figure 7h,i**). Our results further track with the mutants we introduced into UBE2Z and UBE2J2 to repel or prevent interaction with $InsP_6$ (**old Fig. 6g-j, now Fig. 7b-e**) as addition of $InsP_6$ no longer affects FAT10 transfer (**Fig. 7h,i**). Interestingly, we observed an opposite effect on FAT10 transfer to UBE2S whereby FAT10 transfer was promoted in the absence of $InsP_6$ (**Figure 7h,i**). Modelling of UBE2S binding to UBA6 SCCH revealed two negatively charged residues that may repel $InsP_6$ (**Figure 7f**) and mutation of these residues to alanine enabled FAT10 transfer onto UBE2S without noticeably altering ubiquitin transfer (**Figure 7g**). These insights enable us to refine our model and the complex role of $InsP_6$ which for most E2s acts as a molecular glue to facilitate FAT10 transfer. Future studies should investigate how $InsP_6$ levels within cells can vary, leading to further regulation of FAT10 transfer.

Fifth, the authors propose that productive binding of UBE2Z to the UBA6-FAT10 complex is critical for efficient transfer. Based on the presented structures, it would strengthen the mechanistic model if the authors could engineer mutations into other, less efficient E2 enzymes to make them more UBE2Z-like and test whether these substitutions improve FAT10 transfer. This would provide stronger functional validation of the proposed structural mechanism.

We thank the reviewers for this suggestion and we agree it would be fantastic to enable gain-of-function FAT10 activity for other UBA6-compatible E2s. We made several attempts to convert BIRC6 or other E2s into a FAT10 conjugating E2 focussing on introducing mutations in the predicted UBL2-interacting regions which were unfortunately unsuccessful. However, building on our finding of the importance of a compatible interaction between and E2 and $InsP_6$, we did successfully boost FAT10 transfer to UBE2S by removing a repulsive interaction (**Figure 7f,g**). We are sure the reviewers appreciate it is easier to break activity rather than create new activities and this is particularly challenging when there are two opportunities for promoting FAT10 compatibility - compatible interface with UBL2 and favourable interaction with UBA6. As a case in point for the latter, our recent study probing E1-E2 specificities (PMID 41350950) revealed subtle differences between E2s (aspartic acid versus glutamic acid) promoted a favourable E1 interaction and this was only revealed through structures of E1-E2 complexes. We predict that additional UBA6-Ub-E2 and UBA6-FAT10-E2 structures will reveal features bespoke to each E2 that enable or prevent FAT10 functionality.

Finally The conclusion that the FAT10 tail is responsible for E2 selectivity (line 333) is not entirely clear to the reviewer. Figure 5d shows that even when the C-terminal tail residues of FAT10 are retained, the fused UBL loses E2 selectivity. In contrast, Figure 5f shows that the fused UBL retains E2 selectivity even in the absence of the FAT10 C-terminal tail residues. Together, these results suggest that E2 selectivity is determined primarily by the UBL2 domain rather than by the C-terminal tail residues.

Our conclusions are the same as the reviewers' that E2 selectivity is determined by interacting with the UBL2 interface not solely the FAT10 tail residues (lines 336-339 in version submitted for review) and so we apologise for any confusion. We have reworded the text to avoid ambiguity.

Minor points:

1. In the text, the authors refer to Extended Fig. 2e, but this panel is not presented.

We thank the reviewer for spotting this, we have now corrected this mistake.

2. In line 270, it should be Asp97 rather than Glu97, since in Fig. 4e it is labeled as D97.

We thank the reviewer for spotting this, we have now corrected this mistake.

3. Extended Fig. 10 is not clear, particularly the transthiolation of FAT10 onto UBE2Z.

We have adjusted the schematic accordingly and have split the reaction into ubiquitin- and FAT10-directed pathways for clarity.

Reviewer #4 (Remarks to the Author):

I co-reviewed this manuscript with one of the reviewers who provided the listed reports. This is part of the Nature Communications initiative to facilitate training in peer review and to provide appropriate recognition for Early Career Researchers who co-review manuscripts.

We thank the reviewer for their time assessing our manuscript.
