# Supplementary material for: Structural determinants for FAT10 activation and transfer from UBA6 to E2 enzymes
Source: Nat Commun. 2026 Aug 13;17:9751. doi: 10.1038/s41467-026-76603-3 (PMC13572539; doi:10.1038/s41467-026-76603-3)

Source data - Dashed black box = image in main figure. Dashed blue box = area quantified.

Figure 1b

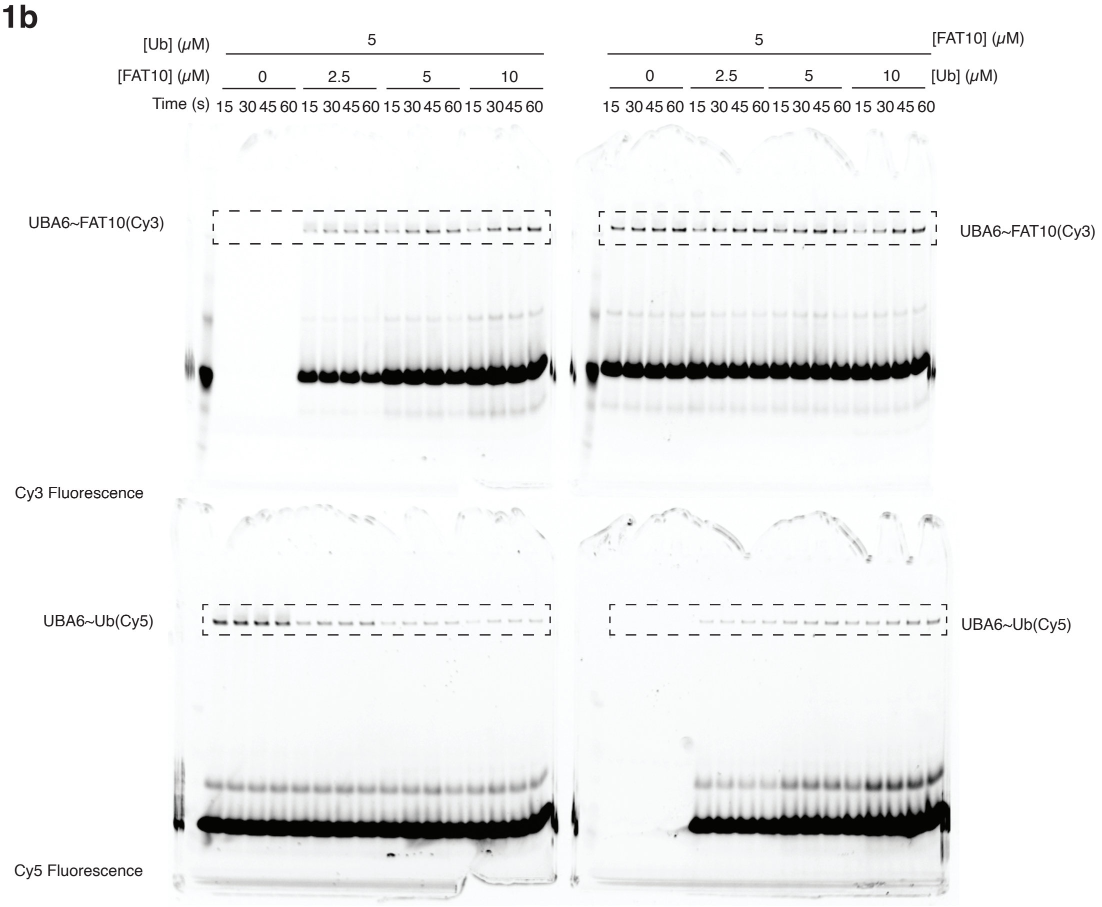

Figure 1c and Supplementary Figure 4c

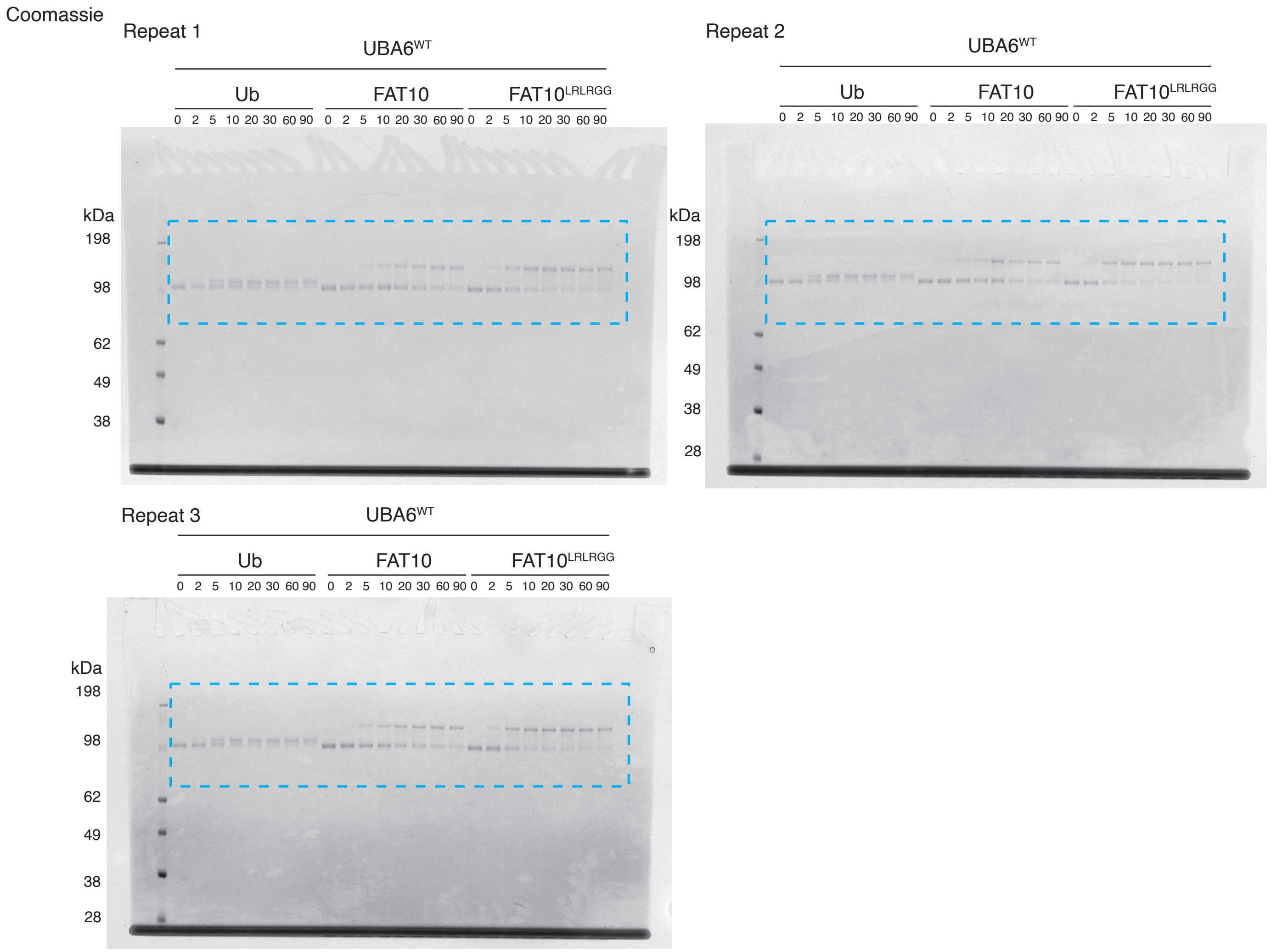

Figure 3a

Cy3 Fluorescence

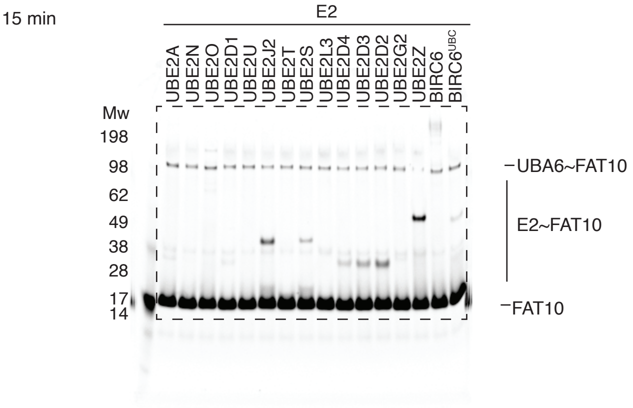

Figure 3b

Coomassie

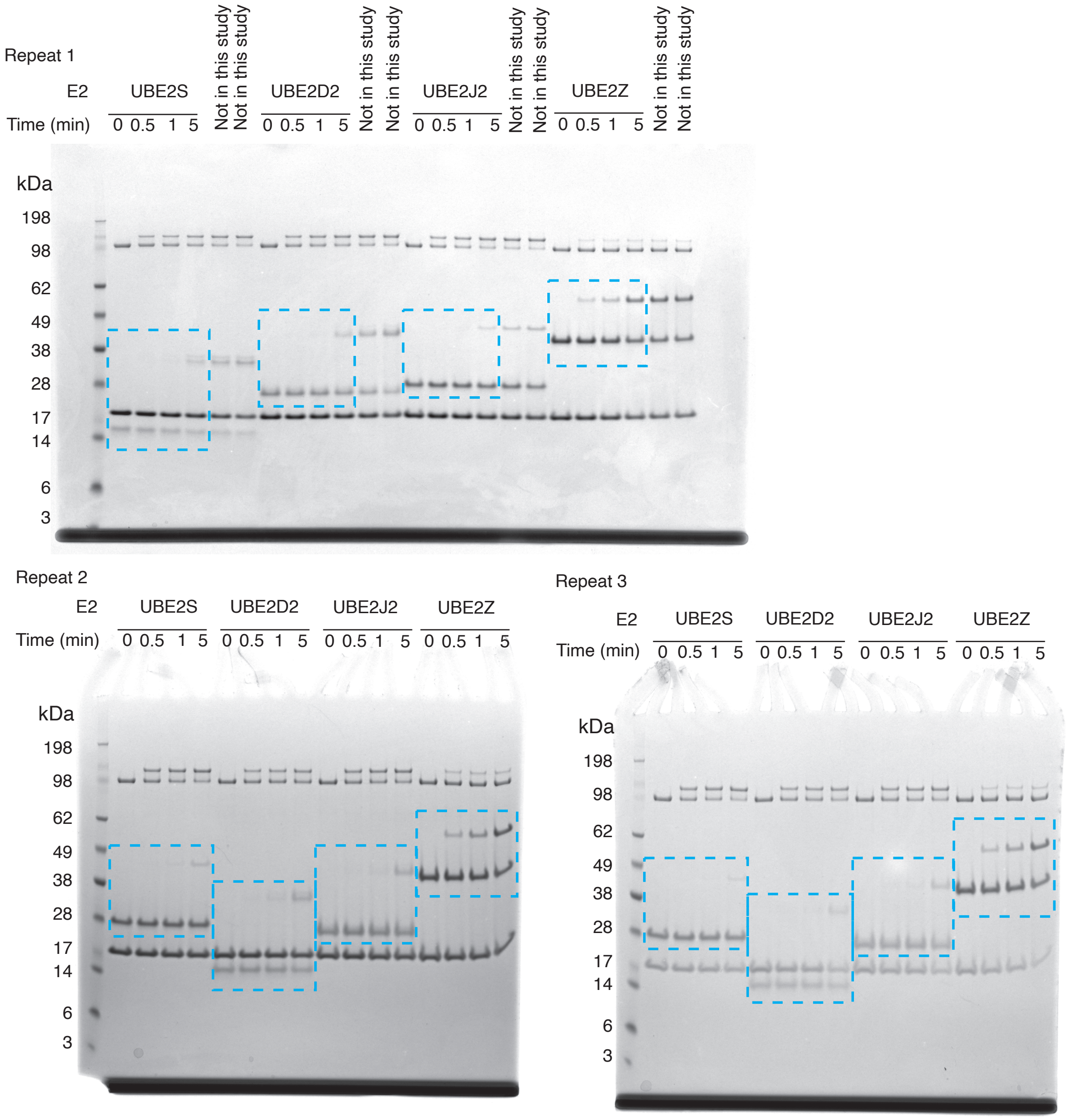

Figure 4g and 7c

Coomassie

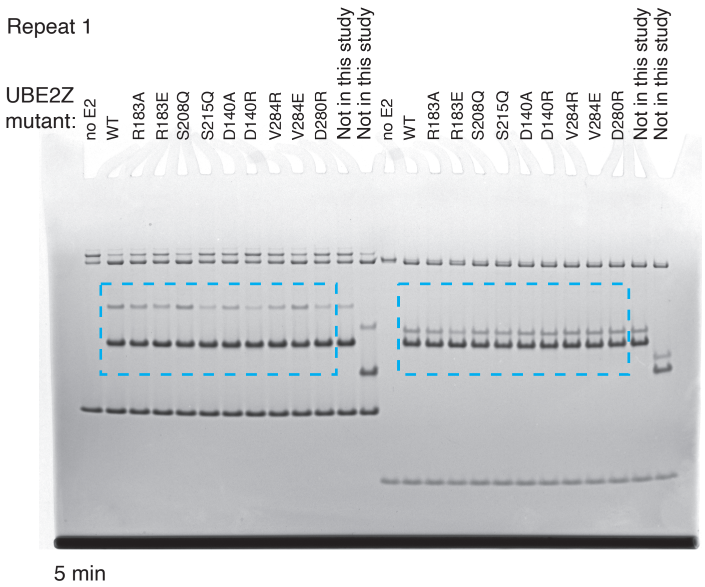

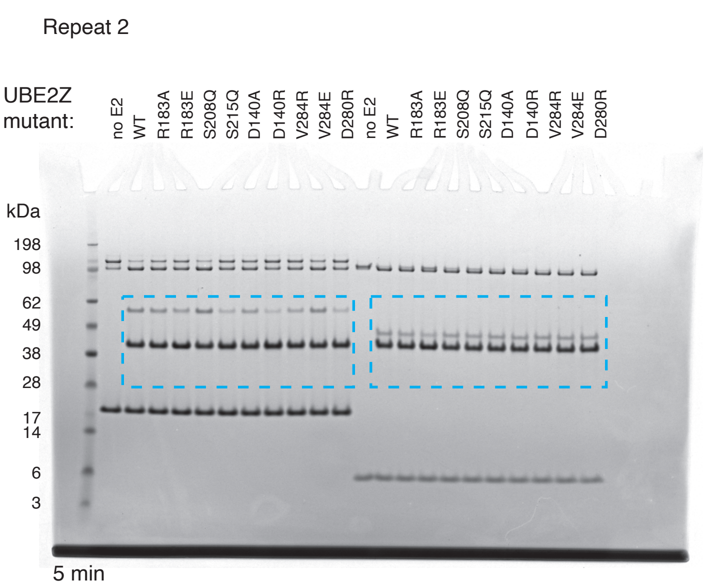

Figure 5d

Cy3 Fluorescence

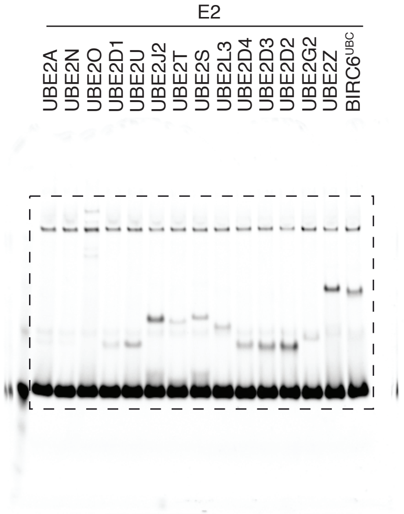

Figure 5e

Cy3 Fluorescence

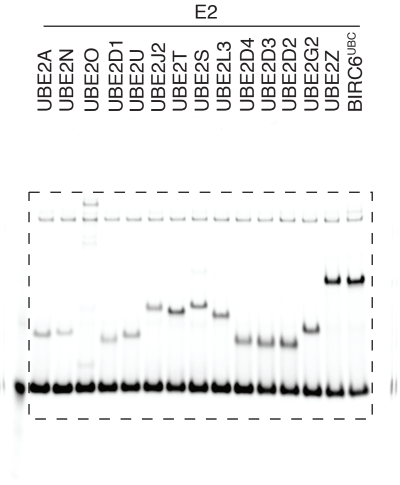

Figure 5f

Cy3 Fluorescence

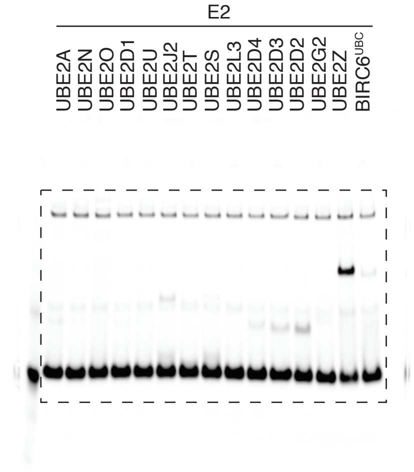

Figure 6b

Coomassie

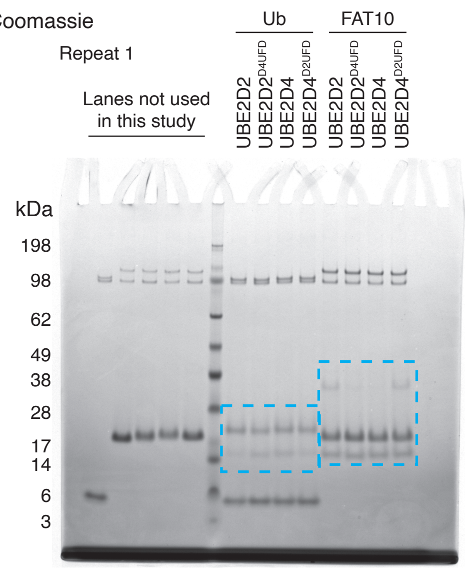

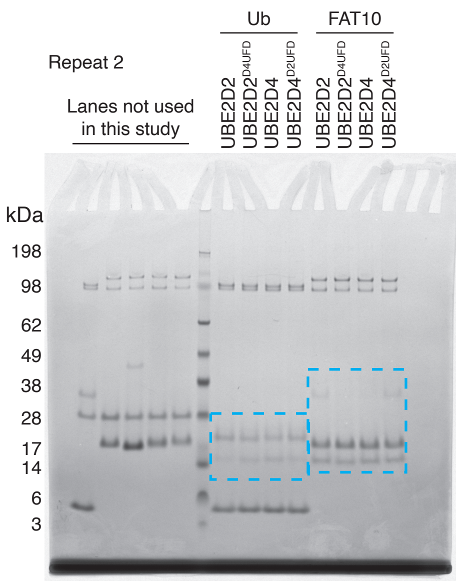

Figure 6c

Cy3 Fluorescence

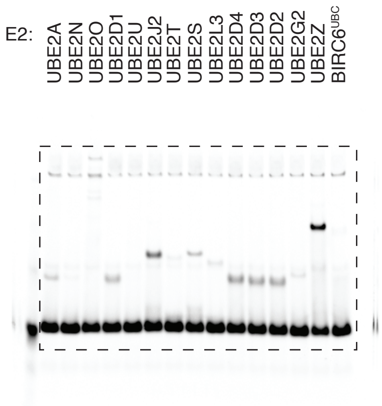

Figure 6e

Coomassie

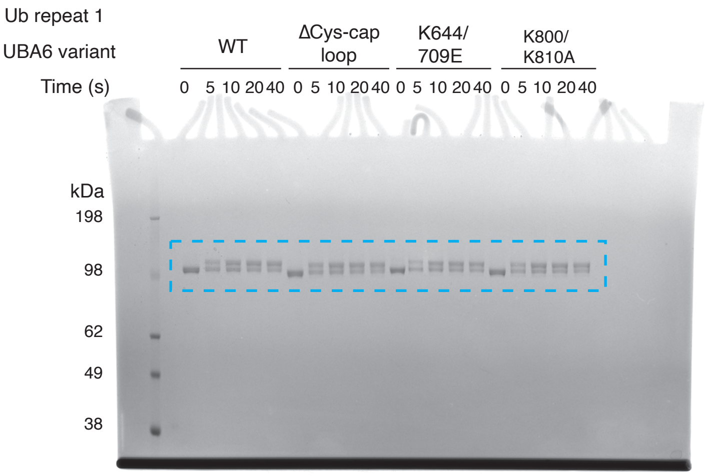

MOPS running buffer to aid separation of UBA6~Ub from UBA6

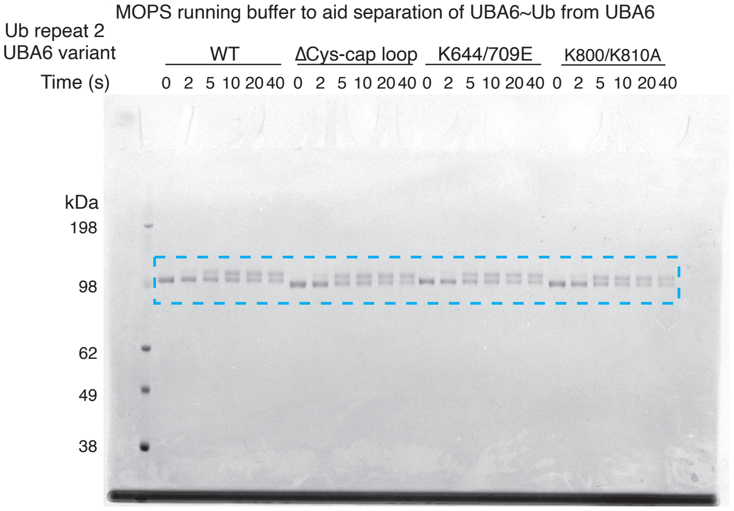

MOPS running buffer to aid separation of UBA6~Ub from UBA6

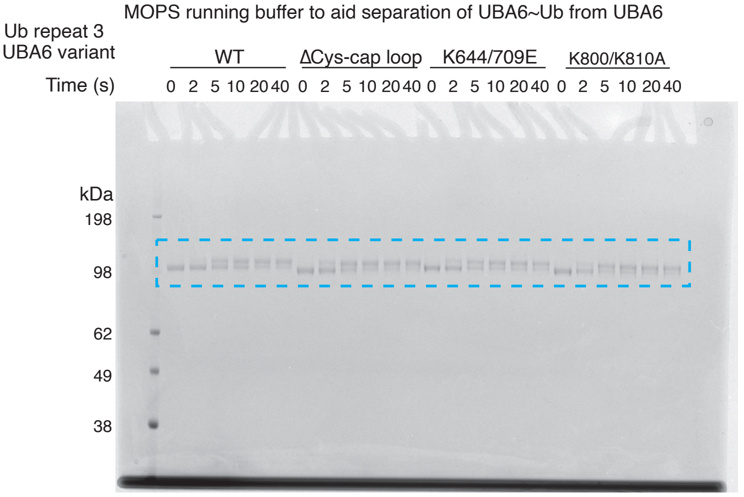

MOPS running buffer to aid separation of UBA6~Ub from UBA6

Figure 6f

Coomassie

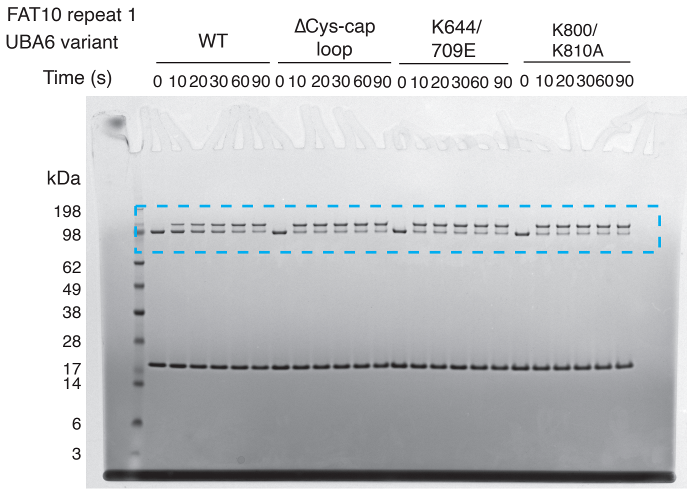

MES running buffer

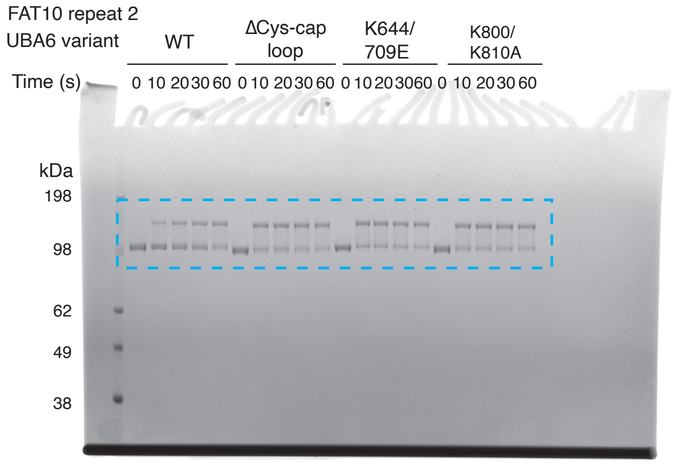

MOPS running buffer to aid separation of UBA6~FAT10 from UBA6

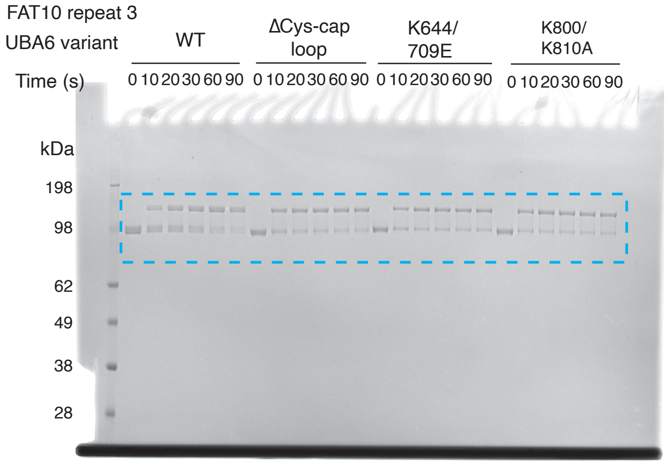

MOPS running buffer to aid separation of UBA6~FAT10 from UBA6

Figure 7c - For 5 min time point, please see Figure 4g

Coomassie

Repeat 1

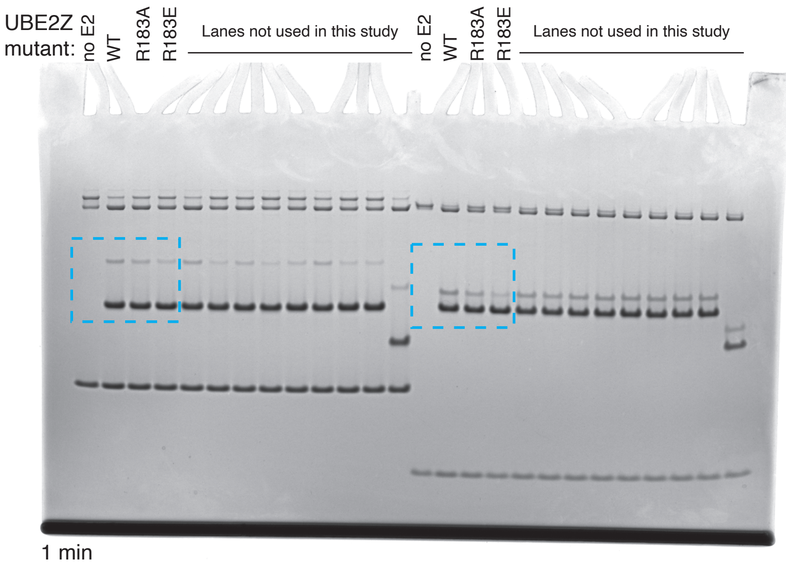

Repeat 2

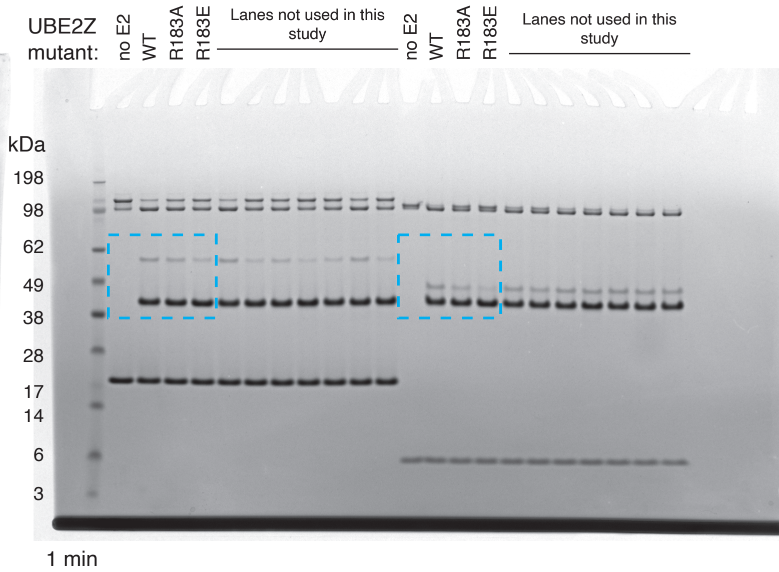

Figure 7e

Coomassie

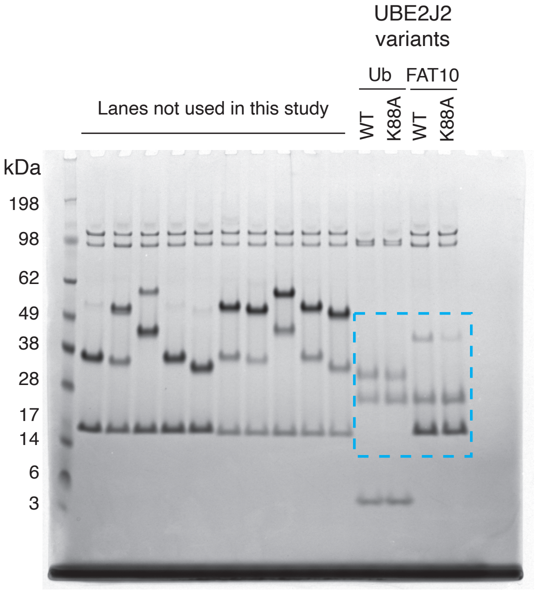

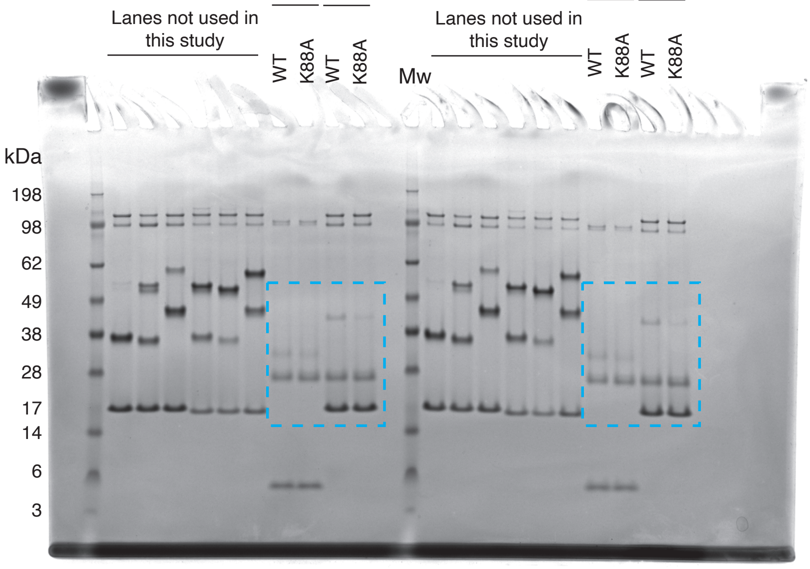

Figure 7g

Coomassie

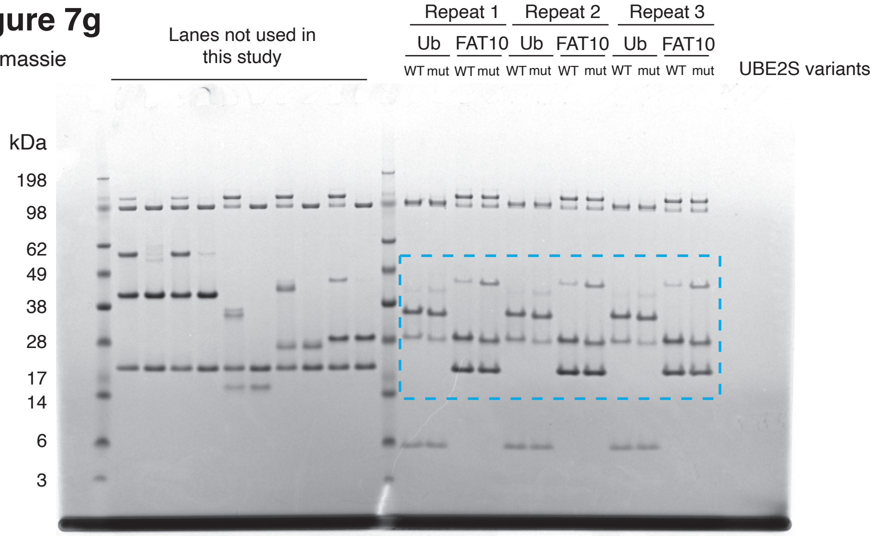

Figure 7h and i

Cy3 Fluorescence

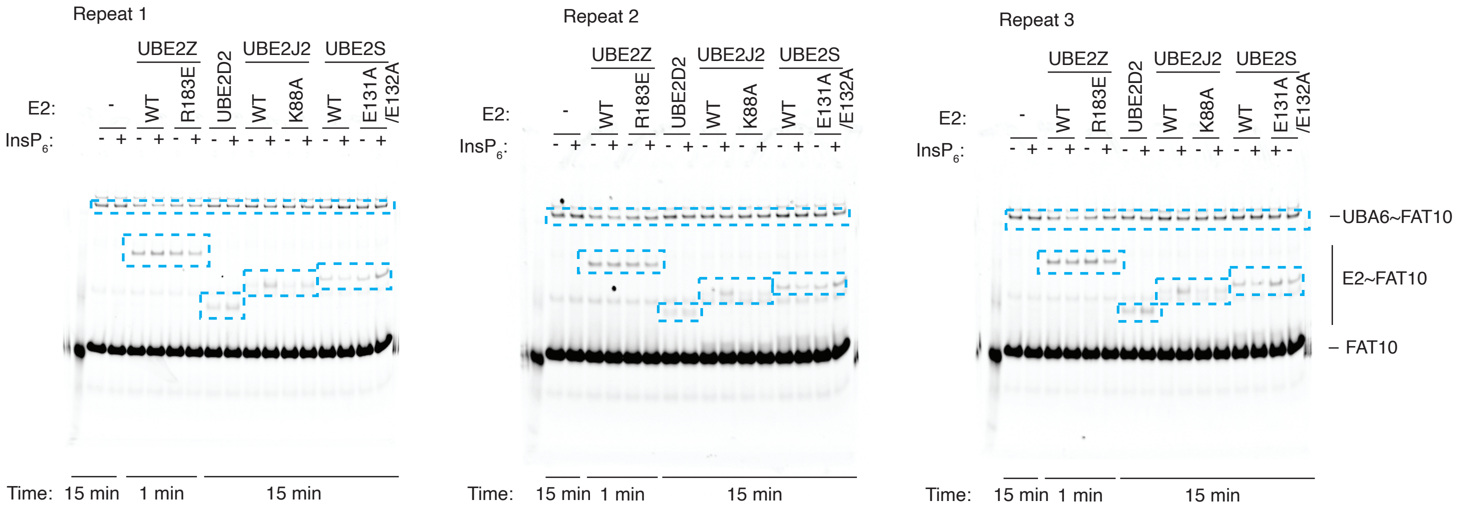

Supplementary Figure 2a

Coomassie

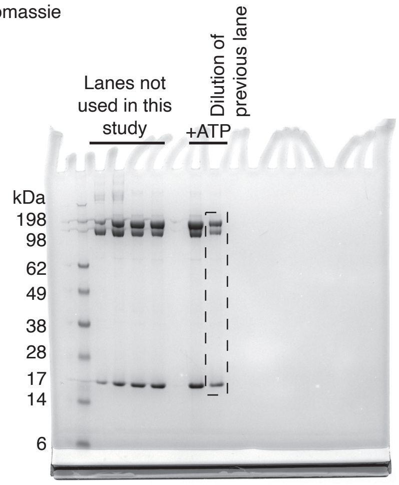

Supplementary Figure 4c - see Figure 1c

Supplementary Figure 5a

Cy3 Fluorescence

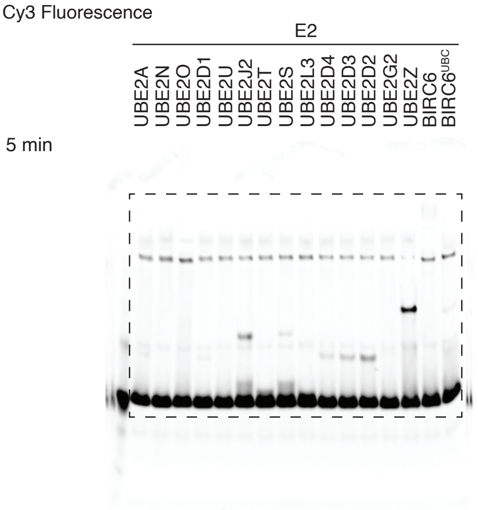

Supplementary Figure 5b

FITC Fluorescence

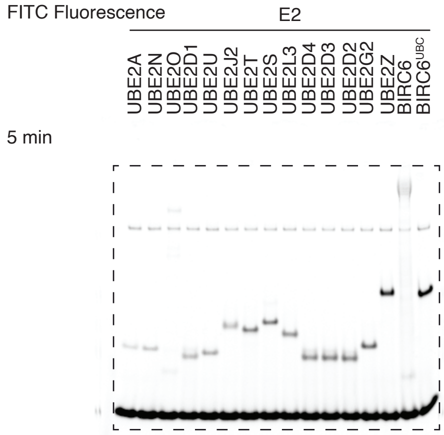

Supplementary Figure 5c

Cy3 Fluorescence

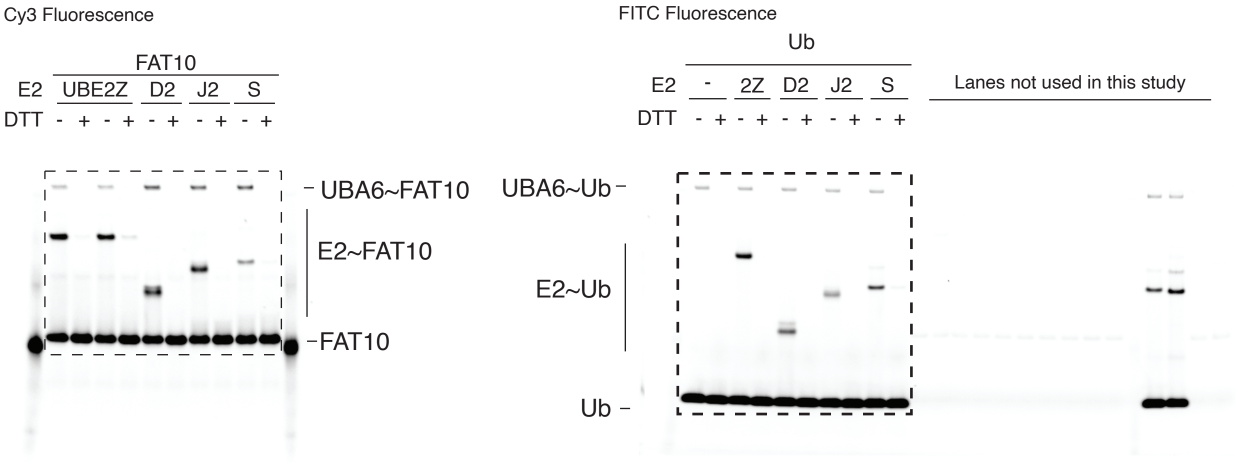

Supplementary Figure 6a

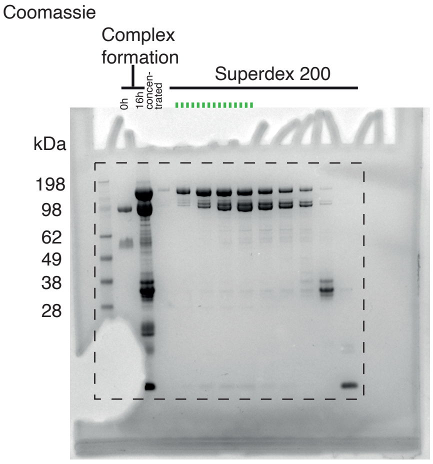

Supplementary Figure 7a

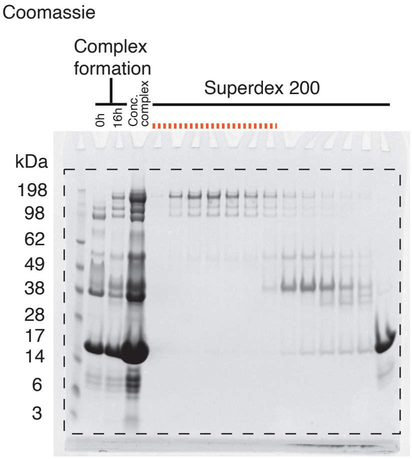

Supplement: Supplementary file 4 [file 41467_2026_76603_MOESM4_ESM.zip › Ellison_CJ-E1-E2_FAT10_mechanism_Figures_SOURCE_IMAGES.pdf]
